# Genome-scale transcriptional study of hybrid effects and regulatory divergence in an F_1_ hybrid *Ruellia* (Wild Petunias: Acanthaceae) and its parents

**DOI:** 10.1186/s12870-016-0962-6

**Published:** 2017-01-17

**Authors:** Yongbin Zhuang, Erin A. Tripp

**Affiliations:** 1 Department of Ecology and Evolutionary Biology, University of Colorado, UCB 334, Boulder, CO 80309 USA; 2 Museum of Natural History, University of Colorado, UCB 350, Boulder, CO 80309 USA

**Keywords:** Anthocyanin, Complementation, Transgressive, *Ruellia*, Hybrid effects, RNA-Seq, Flower color, MYB transcript factors

## Abstract

**Background:**

New combinations of divergent genomes can give rise to novel genetic functions in resulting hybrid progeny. Such functions may yield opportunities for ecological divergence, contributing ultimately to reproductive isolation and evolutionary longevity of nascent hybrid lineages. In plants, the degree to which transgressive genotypes contribute to floral novelty remains a question of key interest. Here, we generated an F_1_ hybrid plant between the red-flowered *Ruellia elegans* and yellow flowered *R. speciosa*. RNA-seq technology was used to explore differential gene expression between the hybrid and its two parents, with emphasis on genetic elements involved in the production of floral anthocyanin pigments.

**Results:**

The hybrid was purple flowered and produced novel floral delphinidin pigments not manufactured by either parent. We found that nearly a fifth of all 86,475 unigenes expressed were unique to the hybrid. The majority of hybrid unigenes (80.97%) showed a pattern of complete dominance to one parent or the other although this ratio was uneven, suggesting asymmetrical influence of parental genomes on the progeny transcriptome. However, 8.87% of all transcripts within the hybrid were expressed at significantly higher or lower mean levels than observed for either parent. A total of 28 unigenes coding putatively for eight core enzymes in the anthocyanin pathway were recovered, along with three candidate MYBs involved in anthocyanin regulation.

**Conclusion:**

Our results suggest that models of gene evolution that explain phenotypic novelty and hybrid establishment in plants may need to include transgressive effects. Additionally, our results lend insight into the potential for floral novelty that derives from unions of divergent genomes. These findings serve as a starting point to further investigate molecular mechanisms involved in flower color transitions in *Ruellia*.

**Electronic supplementary material:**

The online version of this article (doi:10.1186/s12870-016-0962-6) contains supplementary material, which is available to authorized users.

## Background

Because new combinations of divergent genomes can yield novel genetic materials for natural selection, hybridization has been described as an evolutionary stimulus [1, 2]. In land plants, hybridization is rampant and has long been appreciated as an important contributor to the full panoply of speciation mechanisms [3–5]. Up to a quarter of all plants form hybrids with at least one other species, and although many such events result in genomic discordance and hybrid failure, new combinations of divergent parental genomes can alternatively provide a source of genetic and phenotypic novelty [5, 6]. Such novelties yield opportunities for ecological divergence and may contribute to reproductive isolation [5, 7–9].

Molecular processes that emerge from unions of divergent genomes remain incompletely understood yet are critical to reconstructing key events that characterize the evolution of novelty in hybrid systems [10]. Recent studies have, in particular, reinvigorated interest in the role that transgressive effects have in the origin and persistence of new hybrid lineages [5, 11, 12]. Transgressive effects are particularly germane to speciation research because they can provide an immediate path to niche separation between hybrid offspring and parents [13, 14]. Transgressive variation arises during recombination and describes values of a given trait (e.g., phenotype, gene expression) that fall outside the range of values of either parent. In plants, transgressive floral traits that derive from new combinations of divergent parental genomes are exceptionally relevant because floral trait shifts can directly impact reproductive isolation [15].

Flower color is one of the most important and compelling traits in plants for pollinator attraction, and changes in flower color are generally adaptive (reviewed in Wessinger & Rausher 2012) [16]. Flower color is often determined by production of anthocyanin pigments, their associations with metal ions, and the pH of vacuoles in which they are stored [17, 18]. In addition to coloring flowers and fruits, products of the Anthocyanin Biosynthesis Pathway (ABP) accumulate in vegetative portions of plants where they function in UV sunscreening [19]. Because of widespread metabolic significance to numerous organisms, the ABP has been characterized genetically by extensive study of structural and regulatory elements, changes to which can and do impact evolutionary trajectories [20–27]. This rich body of research establishes the ABP as an excellent model pathway in which to explore the impacts of hybridization on floral novelty and transgressive functions. Such processes have been enlightened by study in several model plants e.g., Louisiana Irises [28] and *Ophrys* [29], but remain unexplored in most non-model systems (but see McCarthy et al. 2015) [30].

In present work, we constructed an artificial F_1_ hybrid between the red-flowered *Ruellia elegans* Poir. and yellow-flowered *Ruellia speciosa* Lindau and then generated corolla (i.e., petal) and leaf transcriptome data for the two parents plus the hybrid. These two species were selected for the present study first because we were particularly interested in transgressive effects that arise from the union of divergent (vs. closely related) genomes. *Ruellia elegans* and *R. speciosa* belong to two different lineages within the genus, whose stem groups are separated by at least 1 million years [31]. Second, these species are important from both economic and scientific perspectives: whereas *Ruellia elegans* is widely cultivated in the horticultural industry, a complete draft of the nuclear genome of *R. speciosa* was recently completed, represented only the third family of Asterids with a reference genome sequence [32]. We used these data to (1) quantify transgressive elements in transcriptomes of hybrid progeny, then (2) to assess the overall potential of floral novelty that derives from unions of divergent genomes.

## Results & Discussion

### Phenotypic comparison of the hybrid and its parents

**Fig. 1:**
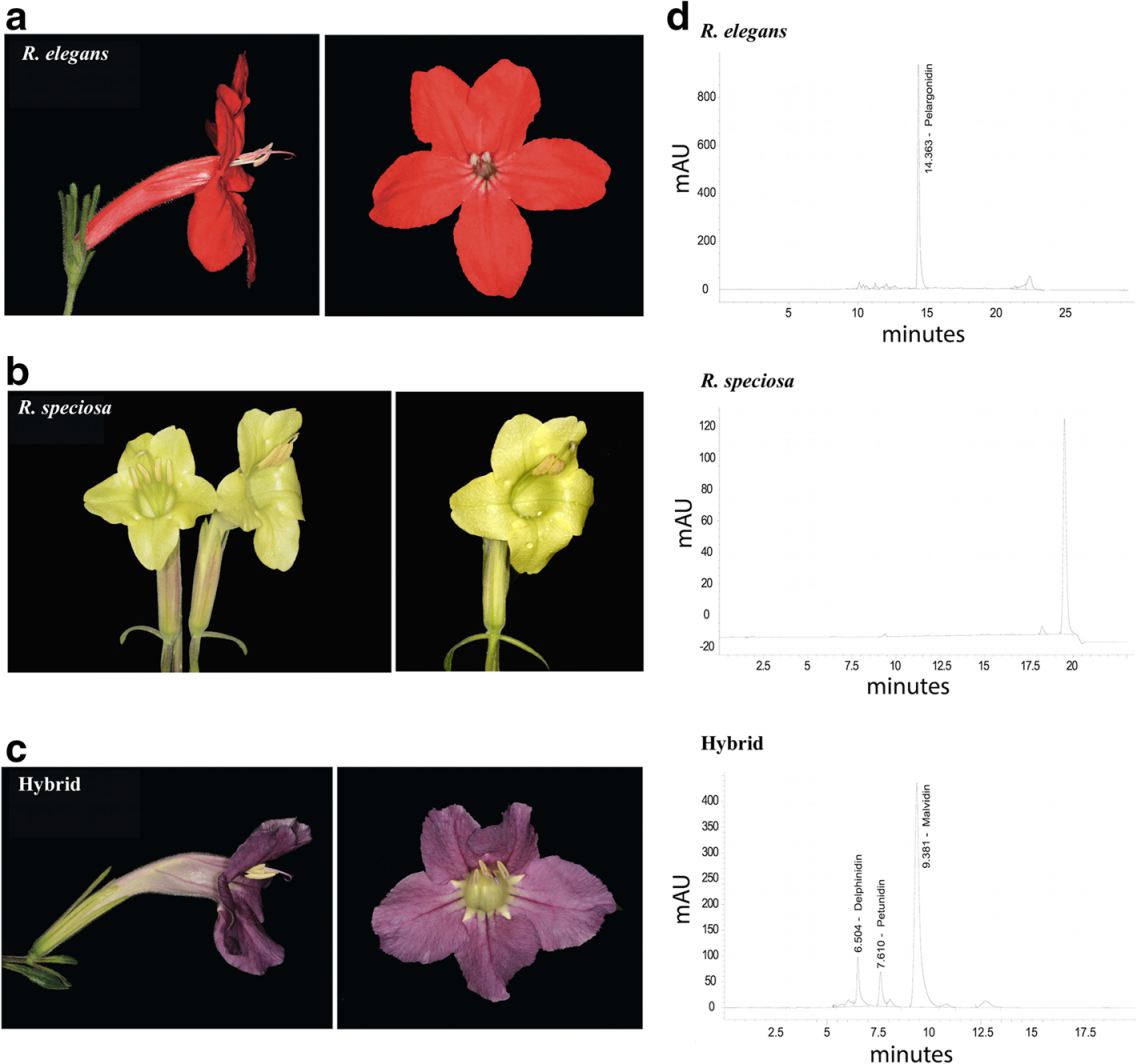
Flower morphologies and HPLC anthocyanin traces of three samples used in transcriptomic analysis. **a** *Ruellia elegans* (*R. elegans*). **b** *Ruellia speciosa* (*R. speciosa*). **c** F_1_ hybrid *Ruellia elegans* x *R. speciosa* (hybrid). **d** Results of HPLC analysis of corollas. In *R. elegans*, only pelargonidin was detected. No anthocyanins were detected in *R. speciosa*. Delphinidin and its derivatives malvidin and petunidin were detected in the hybrid

Morphologically, F_1_ plants of *Ruellia elegans* x *R. speciosa* we generated are an admixture between the two parents but resemble the paternal species to a greater degree than the maternal species (Fig. 1 and Additional file 1: Figure S1). With the yellow-flowered *Ruellia speciosa* paternal plant, hybrid plants share strongly odoriferous vegetative parts, prominent raised lenticels, conspicuously petiolate leaves (these to ~20 mm long), and a woody habit (vs. non-odoriferous, inconspicuous lenticels, sessile leaves, and herbaceous). With the red-flowered *Ruellia elegans* maternal parent, hybrid plants share flowers in dichasia, long-pedunculate inflorescences, and flowers with white nectar guides (vs. solitary flowers, short peduncles, and flowers lacking nectar guides in *R. speciosa*). However, flowers of the hybrid plant are purple in color, in contrast to either parent (Fig. 1). HPLC analysis confirmed that this purple pigment derives from activation of a branch of the ABP not activated in either parent: whereas *R. elegans* manufactures floral pelargonidins and *R. speciosa* does not manufacture any floral anthocyanins, the hybrid manufactures floral delphinidins (Fig. 1).

### Generation of Illumina PE RNA-Seq libraries and de novo assembly

**Fig. 2:**
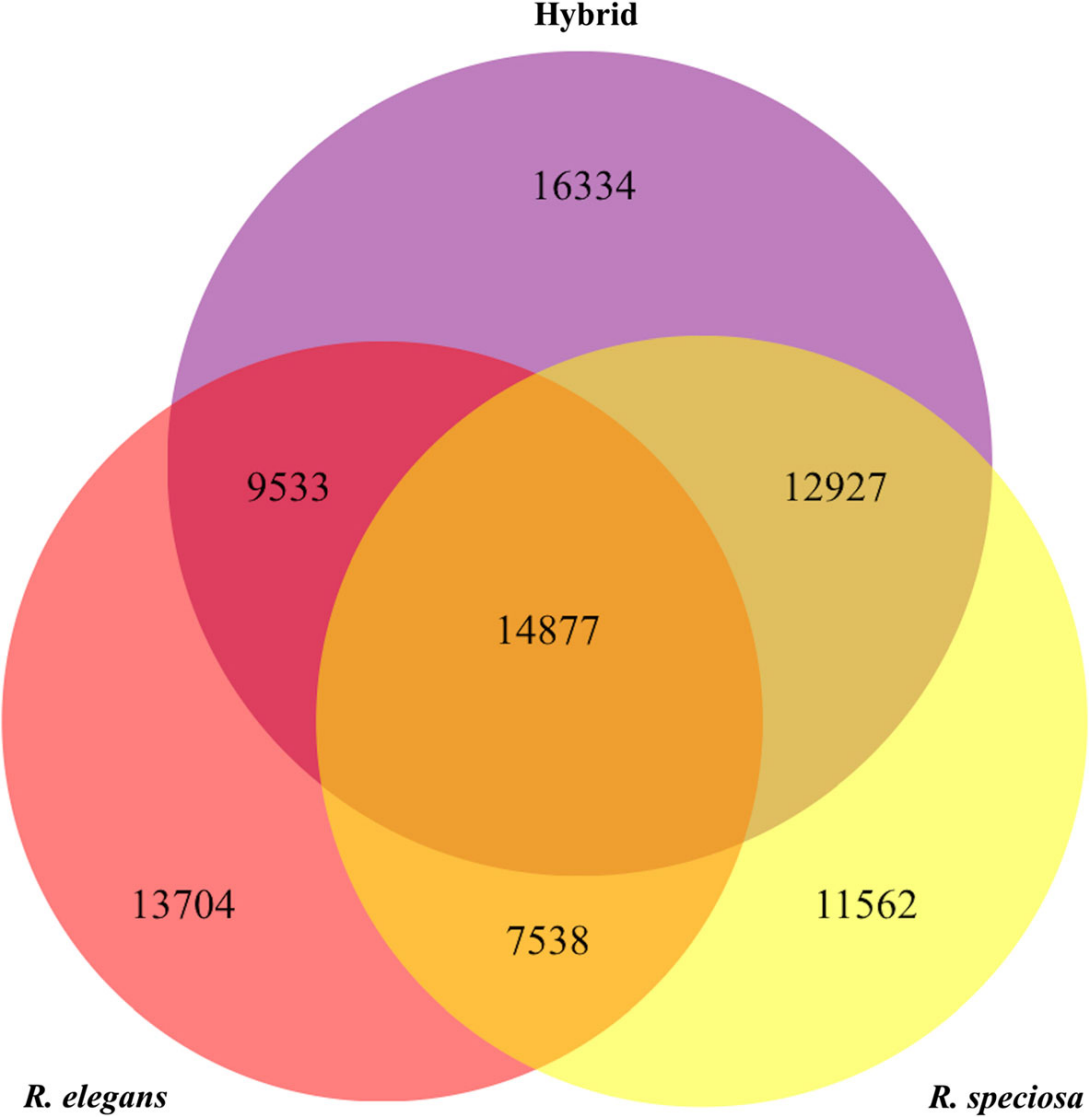
Venn diagram showing the total number of unigenes from each of three assemblies (*R. elegans*, *R. speciosa*, and the hybrid) and the numbers of unigenes shared between each pair of assemblies as well as all three assemblies

In non-model plants without closely related reference genomes, the read generation per sample for *de novo* transcriptome analysis ranges from <100 Mb to ~7 Gb, with 2–5 Gb representing the most common sequencing depths [33]. Because *de novo* assembly requires greater sequencing depth compared to reference-based assembly, we built and sequenced tissue-specific libraries with sequence depths ranging from 2.5Gb to 7.6Gb for DEG (Differential Gene Expression) analysis. We then combined all reads from a given species for an average depth of 16.8Gb, corresponding to 67,231,562 paired-end reads (Table 1). Overall GC and Q20 percentages (sequencing error rate <1%) obtained from the *Ruellia elegans*, *R. speciosa*, and hybrid libraries were 45.01%, 44.39%, and 45.18% and 93.29%, 92.91%, and 95.90%, respectively. Thus, our transcriptome data were of sufficient quantity and quality to ensure accurate sequence assembly and coverage. In total, 44,330, 45,509, and 52,463 unigenes were assembled for *R. elegans, R. speciosa*, and the hybrid with N50 sizes of 1,771, 1,636, and 1,729 bp, (Table 1). Ortholog analysis demonstrated that at least one gene orthologous to genes in the other two species could be found for 74.59%, 69.08%, and 68.87% of total assembled unigenes for *R. elegans, R. speciosa*, and the hybrid (Fig. 2). The Trinotate annotation pipeline yielded high percentages of unigenes for *R. elegans, R. speciosa,* and the hybrid (84.14%, 81.20%, and 82.32) that had at least one hit to reference databases, which may reflect the relatively stringent criteria we used for unigene selection.

**Table 1:** Summary of sequencing and de novo transcriptome assemblies

	*R. elegans*	*R. speciosa*	Hybrid
Reads statistics			
Corolla.rep1	10,014,677	14,539,782	17,375,901
Corolla.rep2	10,864,678	19,562,006	12,844,342
Leaf.rep1	30,358,373	22,548,145	12,321,208
Leaf.rep2	15,355,909	20,003,457	15,909,209
Total	66,593,637	76,653,390	58,450,660
Overall GC (%)	45.01%	44.39%	45.18%
Overall Q20 percentage (%)	93.29%	92.91%	95.90%
Assembled unigene statistics			
Number of unigenes	44,330	45,509	52,463
Total assembly length (bp)	43,094,363	40,225,850	46,796,264
Average length (bp)	972	883	892
N50 (bp)	1,771	1,636	1,729
GC (%)	42.32%	42.51%	42.48%
Percentage of annotated unigenes	84.14%	81.20%	82.32%

### Differential expression gene (DEG) analysis and inheritance classifications

We examined variation among hybrid and parental transcriptomes through a DEGs approach for all possible pairwise comparisons in a tissue-specific manner. Pooled transcripts from leaf and corolla tissues indicated that a comparable number of transcripts were differentially expressed between any given pair of taxa (Table 2). However, on the whole, greater numbers of DEGs were identified in corollas compared to leaves, which may relate to genetic architecture and/or pathway complexity underlying development of the two tissues. There are more unigenes highly expressed in the hybrid than in either parents (Table 2). Overall numbers of DEGs between *R. elegans* and the hybrid were higher than DEGs between *R. speciosa* and the hybrid, suggesting an asymmetrical influence of parental genomes on progeny transcriptomes that has been reported previously [34]. Consistent with the above was a greater morphological similarity of the hybrid to *R. speciosa* than to *R. elegans* (see also Methods).

**Table 2:** Summary of DEGs identified in two parents (*R. elegans*, *R. speciosa*) and an artificial F_1_ hybrid

Tissue	*R. speciosa* vs. *R. elegans*		*R. speciosa* vs. hybrid		*R. elegans* vs. hybrid	
	Higher	Lower	Higher	Lower	Higher	Lower
Leaf	672	314	492	643	819	914
	68.15%	31.85%	43.35%	56.65%	47.26%	52.74%
Corolla	2187	1989	1382	1683	2349	2514
	52.37%	47.63%	45.09%	54.91%	48.30%	51.70%
Overall	2388	1872	1871	2319	2519	2966
	56.06%	43.94%	44.65%	55.34%	45.93%	54.07%

**Fig. 3:**
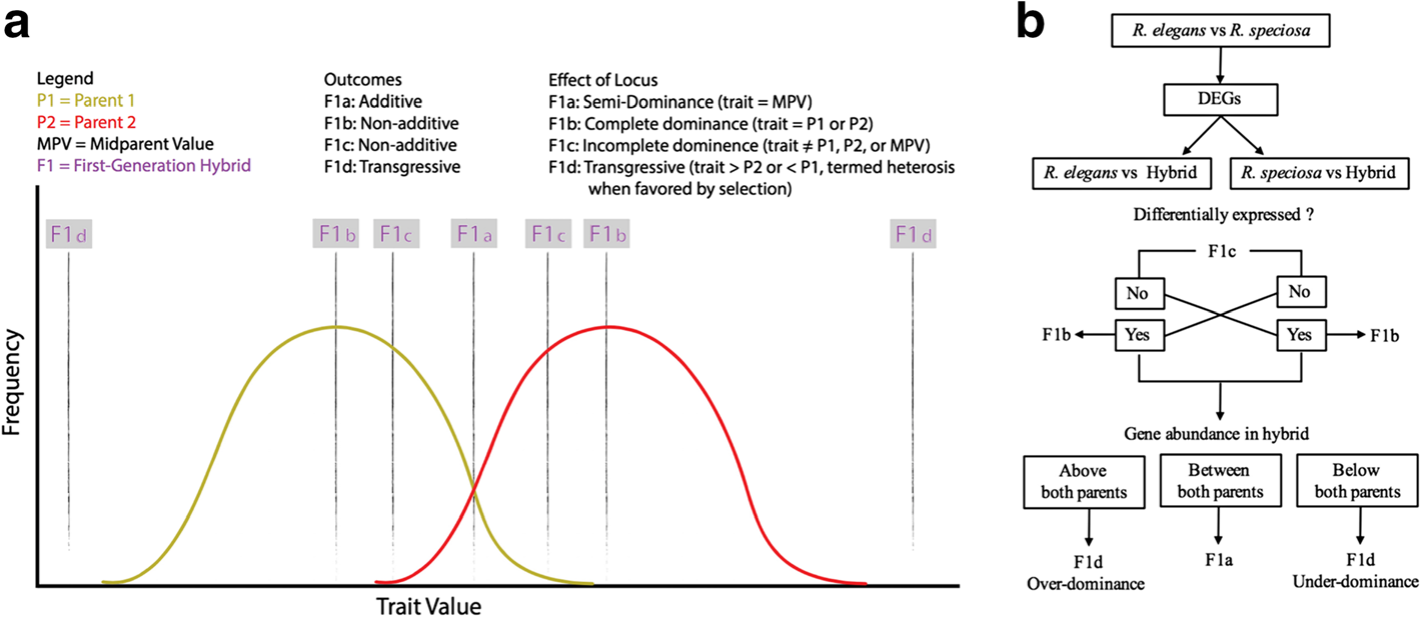
**a** Models of genetic heritability deriving from a simple cross. **b** Workflow illustrating strategy used to determine model of inheritability of DEGs between *R. speciosa* and *R. elegans* in the hybrid. DEG, differential expression gene

Inheritance classifications (Fig. 3; see Methods for explanation) based on unigene abundance in the hybrid compared to its parents yielded marked contrasts among four delimited categories: semi-dominance, complete dominance, incomplete dominance, and transgressive expression (Table 3). Summing data from leaf and corolla transcriptomes (tissue-specific analyses generated similar models of inheritance between leaf and corolla data; Table 3), only 148 unigenes (3.47%) of 4,260 total unigenes identified showed an additive effect (trait = mid-parent value [MPV]) in the hybrid. Instead, the majority of unigenes (3,442; 80.79%) showed a pattern of complete dominance of one parent or the other: 1,849 unigenes (43.40%) exhibited *R. speciosa* dominance and 1,593 (37.39%) exhibited *R. elegans* dominance; the greater overall impact of the *R. speciosa* genome is again consistent with morphological results (Fig. 1 and Additional file 1: Figure S1). A total of 378 unigenes (8.87%) showed a pattern of transgressive expression in the hybrid, among which 288 (76.19%) had an over-dominant effect and 90 (23.81%) had an under-dominant effect. Although transcript accumulation patterns in the hybrid suggest non-additive patterns were the primary mode of inheritance, detailed study of remaining unigenes combined with floral pigment and morphological data indicate that rarer transgressive elements can substantially impact plant phenotype.

**Table 3:** Descriptive statistics of inheritance patterns in F_1_ hybrid

Effect	F1a^a^	F1b^b^		F1c^c^	F1d^d^	
		*R. speciosa*	*R. elegans*		Over-dominance	Under-dominance
Leaf	28	421	337	102	43	55
	2.84%	42.70%	34.18%	10.34%	4.36%	5.58%
Corolla	121	1901	1433	308	312	101
	2.90%	45.52%	34.32%	7.38%	7.47%	2.42%
Overall	148	1849	1593	292	288	90
	3.47%	43.40%	37.39%	6.85%	6.76%	2.11%

a: Semi-Dominance (trait = MPV)

b: Complete dominance (trait = P1 or P2)

c: Incomplete dominance (trait ≠ P1, P2, or MPV)

d: Transgressive (trait > P2 or < P1, termed heterosis when favored by selection)

### Gene enrichment analysis

**Fig. 4:**
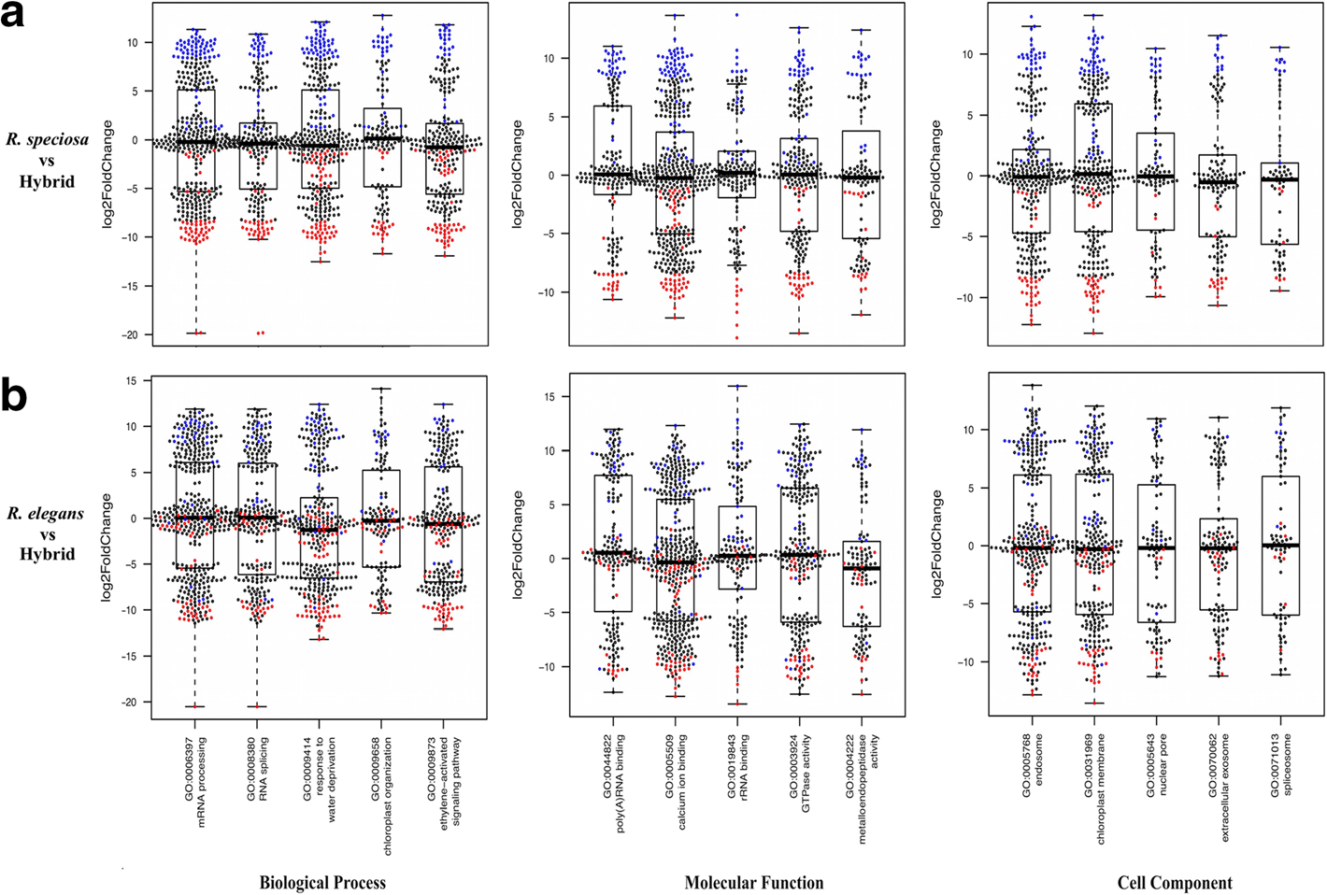
Box plots of the top five most significant GO terms in gene enrichment analyses. Individual gene fold-changes shown by dots. **a** Using the hybrid as a reference, DEGs with higher expression levels were colored *blue* and DEGs with lower expression levels were colored *red*. **b** Shared DEGs between two comparisons were colored as in 4A regardless of their expression levels. Thus, *blue* dots above zero in 4B means that DEGs had lower expression levels in the hybrid compared to both parents while *blue* dots below zero in 4B stands for DEGs expression levels was between two parents

Gene Ontology enrichment analysis is one means of exploring sets of genes that are over-represented or under-represented in a given sample. We conducted GO analyses on *R. elegans* vs. hybrid and *R. speciosa* vs. hybrid (two comparisons) to facilitate further interpretation of specific functional relevance of the DEGs, thus enabling discovery of general classes of regulatory pathways affected by the union of divergent genomes. Using the hybrid as a reference, both comparisons yielded the same top five GO term hits within a given category (i.e., Biological Process, Molecular Function, or Cell Component; Fig. 4). We found that unigenes more highly expressed in the hybrid compared to one parent tended to also be more highly expressed compared to another parent. This pattern also emerged with respect to genes that were under expressed in the hybrid (Fig. 4). Our results corroborate those from prior studies that have revealed aberrant mRNA abundances in interspecific hybrids-either lower or higher than in parental species [35–37]. Under BP, GO:0006397 (mRNA processing) and GO:0008380 (RNA splicing) were significantly differentially expressed between hybrid and either parent. Similarly, GO:0044822 (polyA RNA binding) and GO:0019843 (rRNA binding) in the MF category and GO:0071013 (catalytic step 2 spliceosome) in the CC category were significantly differentially expressed between the hybrid and its parents. Genes under these GO terms function primarily in mRNA stability, processing, splicing and degradation. Other significant GO terms were involved in plant hormone signaling pathways, protein processing, and chloroplast organization. An overall greater number of genes were transcriptionally activated in the hybrid compared to either parent and several of these were hybrid specific (Table 1; Fig. 2). This may in part be explained by responses to genetic and epigenetic instabilities in resultant homoploid or allopolyploid hybrids, a phenomenon known as genome shock [38, 39]. For example, alterations to DNA replication and perturbation of chromatin structures may induce the release of transposons and aberrant RNA transcripts, and DEGs enriched in pathways that maintain the stability of novel transcripts and degrade aberrant transcripts may be necessary for hybrid function [40, 41].

### Candidate structural genes involved in anthocyanin biosynthesis

**Fig. 5:**
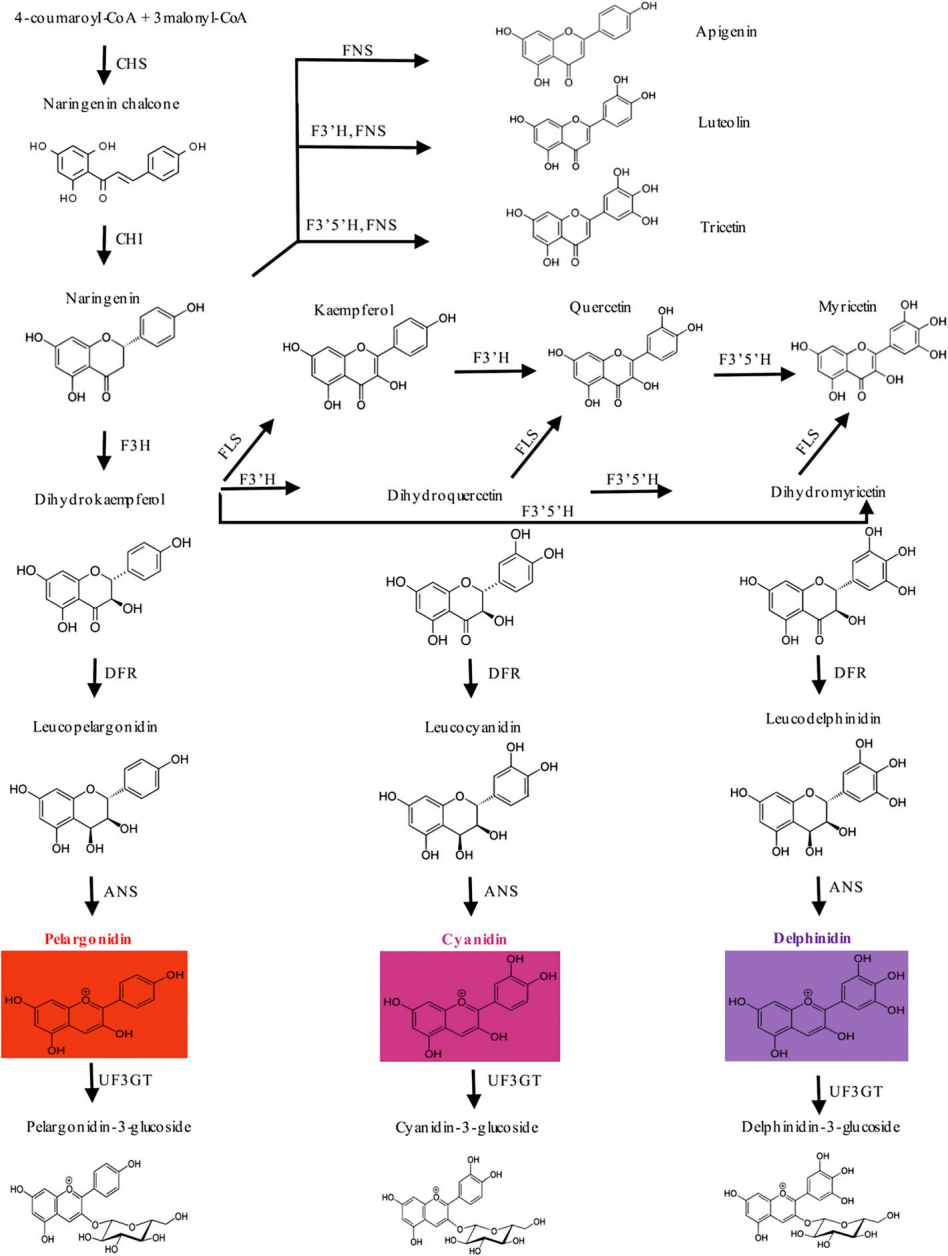
A general schematic diagram of flavonoid biosynthetic, with emphasis on flavones (apigenin, luteolin, tricetin), flavonols (kaemperol, quercetin, myrcetin), and anthocyanins (pelargonidin, cyanidin, delphinidin; not shown are peonidin, malvidin, and petunidin, but peonidin is a derivative of the cyanindin pathway and malvidin and petunidin are derivatives of the delphinidin pathway). Enzymes that catalyze reactions in the Anthocyanin Biosynthetic Pathway include: ANS, anthocyanidin synthase; CHI, chalcone isomerase; CHS, chalcone synthase; DFR, dihydroflavonol 4-reductase; F3H, flavanone 3-hydroxylase; F3'H, flavonoid 3' -hydroxylase; F3'5' H, flavonoid 3',5'-hydroxylase; FLS, flavonol synthase; FNS, flavone synthase; UF3GT, UDP glucose flavonoid 3-glucosyltransferase

**Fig. 6:**
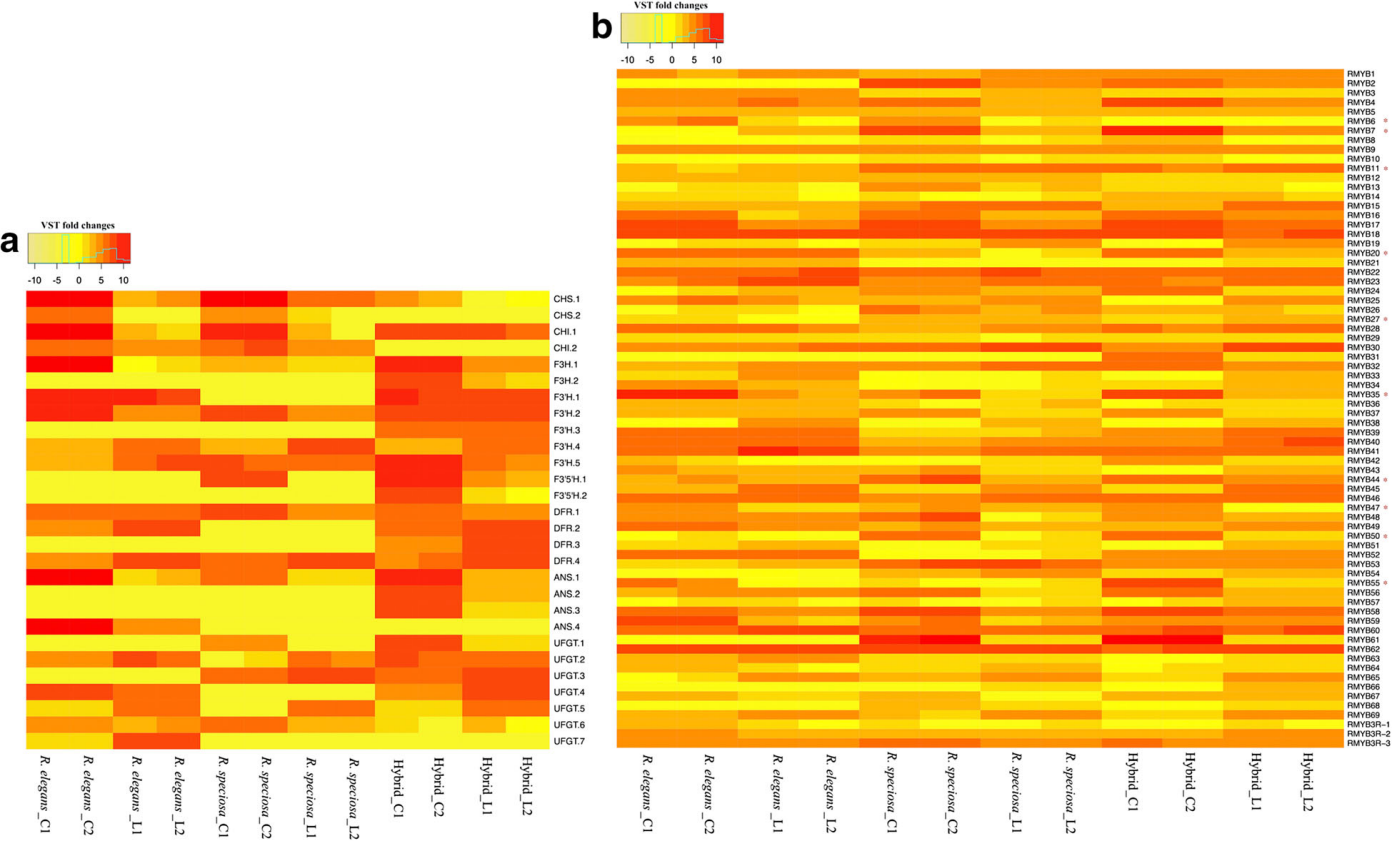
Heat map showing expression patterns of candidate anthocyanin loci in *Ruellia*. **a** 28 putative structural genes and (**b**) 72 MYB type transcription factors. Warmer colors (red) indicate higher expression. Two biological replicates shown as C1, C2 for corolla tissue and L1, L2 for leaf tissue. VST, variance stabilizing transformation

Anthocyanin biosynthesis, a primary branch of the larger flavonoid pathway, is one of the most extensively studied pathways in plants and is highly conserved in angiosperms (Fig. 5) [42]. We recovered a total of 28 structural genes predicted to be functional in anthocyanin biosynthesis (Fig. 6), including two chalcone synthase (CHS), two chalcone isomerase (CHI), two flavanone 3-hydroxylase (F3H), five flavonoid 3'-hydroxylase (F3'H), two flavonoid 3',5'-hydroxylase (F3’5’H), four dihydroflavonol 4-reductase (DFR), four anthocyanidin synthase (ANS) and seven UDP-glucose flavonoid glucosyl transferase (UFGT). Aspects of the above ratios corroborate prior studies that have found comparatively high copy numbers for DFR (*Lotus japonicus* [43]; cherries [44]; red leaf lettuce [45]), UFGT (columbines [46]; cherries [44]; *Stellera chamaejasme* [47]), F3H (peonies [48]), and ANS (*Zoysia* [49]). Similarly, our finding of a low copy number for F3'5'H corroborates data from the above studies (one prominent exception is grapevines, which have been found to have exceptionally high copy variants of this enzyme [50]). Duplications in genes involved in secondary metabolism or responses to environmental stimuli, such as in the ABP, are commonly maintained evolutionarily and have high intraspecific variation in expression patterns [51].

As shown in Fig. 1, in contrast to either parent, flowers of the hybrid plant were purple, resulting from the production of delphinidins—a pathway not activated in either parent (Fig. 5). F3'5'H is the key enzyme that acts to convert DHK or DHQ into dihydromyricetin (DHM), which is a precursor of delphinidins (Fig. 5). In this study, only two copies of F3'5'H were recovered. Both copies were highly expressed in the corollas of the purple-flowered hybrid, and F3'5'H.1 was additionally highly expressed in the corollas of *R. speciosa* even though the latter is yellow-flowered. In the hybrid, F3'5'H.1 showed an ~3.3-fold increase over expression in *R. speciosa*, thus displaying an overdominant effect (Figs. 3 and 6). Amino acid sequence alignment indicated that F3’5’H.1 in the hybrid was identical to F3'5'H.1 in *R. speciosa* but that F3’5’H.2 contains an indel and a premature coding sequence, one or both of which may render it non-functional (Additional file 2: Figure S2). Thus, it is likely that the hybrid inherited its functional copy of F3'5'H.1 from the *R. speciosa* parent, which accumulates anthocyanins only in vegetative and not floral tissue. As a result of hybridization and likely through some complementation effects derived from the *R. elegans* genome (this or these precursors not present in *R. speciosa*), the hybrid possesses and expresses all the necessary genes for floral delphinidin production. In *R. speciosa*, only single copy of two other key enzymes, F3H and ANS, were identified and expressed at extremely low levels compared to expression in *R. elegans* and the hybrid. The continued expression of F3'5'H in corollas of *R. speciosa* may reflect an evolutionary vestige from a prior time period in which the delphinidin pathway as a whole was functional in ancestors, and subsequently only portions of this pathway have degenerated, i.e., there has since been insufficient time to accumulate mutations in F3'5'H specifically and/or its regulators (see phylogenetic history documenting the sister group relationship of the delphinidin-producing *Ruellia hirsuto-glandulosa* to the clade containing *Ruellia speciosa* and other yellow-flowered species [52]). We caution, however, that the transcriptome data presented here serve only as a first step towards understanding the evolution and expression of ABP loci in *Ruellia*. Genetic differences responsible for differences in flower color in this system await future analyses that specifically investigate molecular mechanisms and functional verification of candidate loci.

### Characterization of MYB domain containing proteins

**Fig. 7:**
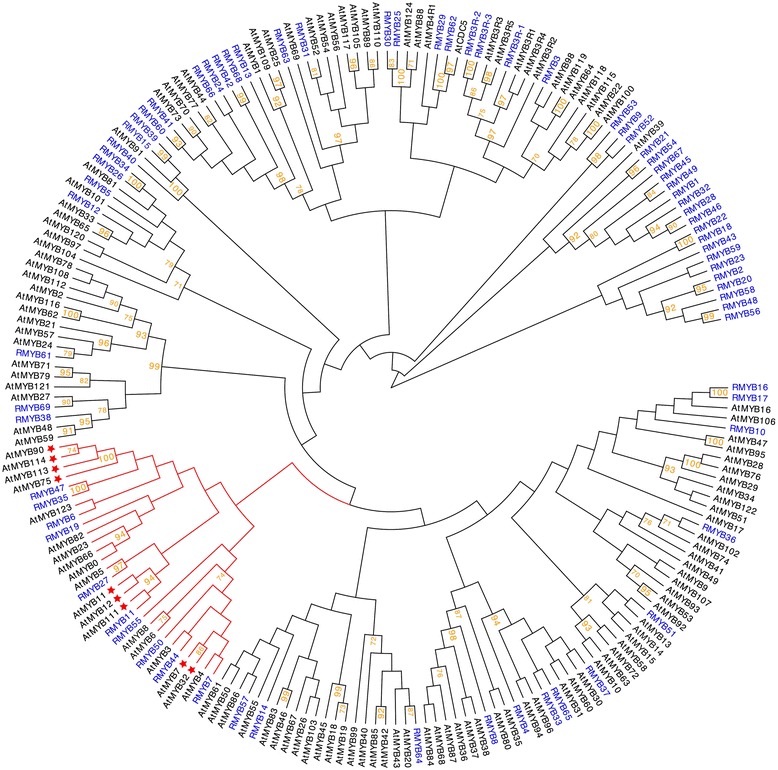
Phylogenetic analysis of *Ruellia* (*blue*) and *Arabidopsis thaliana* (*black*) MYBs. The clade marked by *red branches* contains MYBs that have undergone functional validation for flavonoid biosynthesis in *Arabidopsis* (specific, validated MYBs marked with *red stars*). Bootstrap support for branches with ≥ 70% support labeled

The MYB family of proteins is large, functionally diverse, and represented in all eukaryotes [53]. Most MYB proteins function as transcription factors and are involved in controlling processes that range from development to differentiation, metabolism, responses to biotic and abiotic stresses, and defense [54]. R2R3-MYB proteins (2R-MYBs) represent a major transcription factor family in higher plants and function in a variety of plant-specific processes including anthocyanin biosynthesis [53, 54]. In present work, we recovered a total of 219 MYB DNA binding domain-containing proteins. Of these 219 MYBs, 69 were identified as typical R2R3-type MYBs, three were R3-type MYBs, and the rest were classified as MYB-like genes and were here omitted prior to phylogenetic analysis (Additional file 3: Table S1). *Arabidopsis thaliana* transcription factors AtMYB7, AtMYB11, AtMYB12, AtMYB75, AtMYB90, AtMYB113 and AtMYB114 have been demonstrated to be functional in flavonoid biosynthesis, which is the broader pathway that includes anthocyanins [55–58]. Phylogenetic analysis identified ten *Ruellia* MYBs belongings to the same clade as putatively functional flavonoid MYBs in *Arabidopsis* (Fig. 7). Gene expression profiling showed RMYB7, RMYB35, RMYB50 and RMYB55 to be expressed in a corolla specific pattern and we postulate these as candidate regulators in flower color determination (Fig. 6b). Inheritance classification of RMYB7 and RMYB55 suggests these loci were over-dominant (transgressive expression). Ongoing research (Y. Zhuang & E. Tripp, in prep.) aims to further elucidate the role of these and other regulatory candidates in anthocyanin biosynthesis in *Ruellia*, thus expanding study of transgressive effects in flower color evolution.

## Conclusion

Numerous researchers have documented evolutionary novelty that arises from interaction of foreign genomes [59]. Understanding patterns of differentially expressed genes contributes new insights into genomic mechanisms and rearrangements that yield evolutionary novelty. In particular, analysis of specific changes to expression patterns of candidate loci involved in anthocyanin biosynthesis sheds new light on how hybridization may contribute to flower color evolution, potentially through genetic complementation. In this study, we found 16,334 unigenes (of 86,474 total; 18.89%) that were expressed only in the *Ruellia elegans* x *Ruellia speciosa* hybrid. HPLC analyses of floral anthocyanins indicated that hybrid plants manufactured pigments derived from a branch of the ABP (delphinidins and derivatives) not activated in either parent. The hybrid additionally expressed novel corolla MYBs, a family of transcription factors crucial to anthocyanin production. Our results corroborate prior findings wherein new combinations of divergent genomes within hybrid progeny can give rise to marked transcriptomic changes, several among these transgressive [34, 60]. These data add to a growing literature documenting transgressive gene expression during hybrid lineage formation. The fact that some of these elements underlie the expression of a novel floral phenotype suggests the potential of such effects to contribute to ecological divergence and/or evolutionary novelty [28–30]. Thus, models of gene evolution to explain the establishment of hybrid lineages should include transgressive effects.

Finally, our results serve as a starting point to investigate specific molecular mechanisms that explain flower color transitions in *Ruellia* (Wild Petunias, ~350 species; Acanthaceae family). Namely, these data establish genomic resources for this large lineage of flowering plants in which numerous evolutionary transitions in flower color have occurred, some of which do not adhere to common evolutionary trajectories typical of most other flowering plants [61] and have not yet been investigated from a molecular or functional perspective.

## Methods

### Plant Materials and greenhouse protocols

For this study, *Ruellia elegans* was acquired from the living collections at Royal Botanic Garden, Kew (vouchered in 2016 in the University of Colorado Greenhouses, *E. Tripp* et al. *4594* [COLO]) and *R. speciosa* was acquired from the only known living population of this species (vouchered in 2006, *E. Tripp & S. Acosta 175* [DUKE, MEXU]). The two parental species were grown in the University of Colorado Greenhouses under controlled conditions. We attempted a minimum of 10 artificial, bidirectional crosses between the species, but only the *Ruellia elegans* (maternal) x *Ruellia speciosa* (paternal) cross yielded viable seed (full crossing data unpublished, ms in preparation by E. Tripp, H. Stone, & K. Dexter). Viable seeds of F_1_ progeny were sown and raised under similarly controlled conditions. The resultant *Ruellia elegans* x *R. speciosa* hybrid was vouchered in 2016 in the University of Colorado Greenhouses (*E. Tripp* et al. *5794* [COLO]).

### Anthocyanidin identification and quantification

To detect major anthocyanidins derived from anthocyanins, HPLC analysis was conducted following Harborne [62] with minor modifications. We placed 25 mg of silica-dried leaf or corolla tissue from *R. elegans*, R. speciosa, and hybrid into 2 ml screw-top tubes filled with 1.5 ml 2 N HCl, then vortexed to ensure full envelopment of tissues in acid solution. To cleave sugars from anthocyanin molecules, samples were placed in a 103 °C heat block for 90 min (90 mins yielded a more complete reaction than standard 60 min heat baths). Samples were removed from dry baths and cooled to room temperature. The liquid fractions were transferred to new tubes and centrifuged for 5 mins at 10,000 rpm to obtain supernatants. Retained supernatants were washed twice with 400 uL of ethyl acetate, vortexed, then centrifuged for 1 min at 10,000 rpm to restore phase separation. After removal of the ethyl acetate layer (this retained for additional non-anthocyanin flavonoid HPLC analyses), the pigmented bottom layer was washed twice with 200 uL of isoamyl alcohol to remove remaining HCl. Extracts were injected into an Agilent 1260 Infinity system (Thermo Scientific). Delphinidin chloride, cyanidin chloride, peonidin chloride, malvidin chloride, and petunidin chloride were used as standards. Pigments were separated using a reverse phase Eclipse ZOBRAX XDB-C18 Rapid Resolution Threaded Column (4.6 × 150 mm, 5 μm; Agilent Technologies) following a linear gradient in the mobile phase: Solvent 1 (2% TFA in H_2_0): 85%, 87.5%, 90%, 95% between 0, 6, 10, and 15 mins; Solvent 2 (0.1% TFA in 1-propanol): 16%, 12.5%, 10%, 5% between 0, 6, 10, and 15 mins. Separated pigments were detected using a UV–vis Diode Array Detector coupled to the HPLC and set to 540 nm.

### cDNA library construction and sequencing

Fresh leaf material from mature leaves and corolla tissue from mature buds were removed from *R. elegans*, R. speciosa, and hybrid in duplicate (i.e., two libraries from similar developmental stages were prepared from each species). Samples were placed immediately into liquid N_2_ and total RNA was extracted using a MasterPure™ RNA Purification Kit (Epicentre). The extracted total RNA was treated with DNaseI and further purified to remove DNaseI, salts and other organics according to the manufacturer’s protocols. RNA integrity was determined on an Agilent 2100 Bioanalyzer. ScriptSeq Complete Kit-Low Input (BL1224, Illumina) was used to prepare RNA-seq libraries from purified RNA following the manufacturer's instructions. The final libraries were quantified using a Qubit (Invitrogen) and quality checked on a Bioanalyzer. Libraries were sent to the Genomics and Microarray Core, University of Colorado–Anschutz Medical Campus then sequenced on an Illumina HiSeq2500 using 2x125 bp paired-end (PE) chemistry. Sequences are on deposit at NCBI: http://www.ncbi.nlm.nih.gov/bioproject/PRJNA323650. SRA accession: SRP075855.

### De novo assembly and gene annotation

Raw reads were filtered to remove low quality bases using Trimmomatic [63] and parameters described in the manual, namely ‘LEADING:3 TRAILING:3 SLIDINGWINDOW:4:15 MINLEN:36’. QC statistics were calculated using iTools (github.com/BGI-shenzhen/Reseqtools/blob/master). Tissue-specific reads for a given species that passed quality filtration were combined and assembled *de novo* using Trinity software release v2.2.0 [64] with a minimum contig length cutoff of 200 bp. Bowtie2 release v2.2.5 [65] and eXpress [66] were used to map combined quality trimmed reads back to initial assembled transcripts and to estimate transcript abundance. Trinity groups all possible transcript isoforms into clusters based on their contents using a unique gene identifier. First, to identify unigenes from each gene cluster, we conducted ortholog searches using Proteinortho [67] among our raw assemblies of *R. speciosa*, the hybrid, and *R. elegans*. Second, we retained transcripts if FPKM values were >2 or if orthologs could be found in either of the two parental species. For gene clusters without identifiable orthologs, the longest transcript (thus accounting for alternative splicing) was selected as a representative unigene. Whole transcriptome functional annotation and analysis was conducted using the Trinotate pipeline release version 3.0.0 (http://trinotate.github.io), with a blast e-value threshold of 1 × 10^− 5^. To assign function annotations to the assembly, this pipeline utilizes NCBI-BLAST [68] to search for homologies between the assembly and SwissProt or custom provided databases and query-curated annotation databases (EMBL Uniprot eggNOG/GO Pathways databases). Additionally, it makes use of other well-referenced tools for functional annotation such as HMMER/PFAM (protein domain identification), signalP/tmHMM (protein signal prediction), and RNAMMER (rRNA annotation). Finally, in addition to Trinotate and SwissProt, all green plant entries integrated into UniProtKB (Taxonomy: Viridiplantae) were retrieved to serve as a custom database for blast searches.

### Differential expression gene (DEG) analysis

The R packages edgeR [69] and DESeq2 [70] were used for differential gene expression analysis. To generate count tables for each unigene, reads from each tissue-specific library were mapped back to their corresponding species-specific assembly. eXpress was used to measure gene abundance with the parameter ‘no-bias-correct’ in effect. The ‘est_counts’ column of tissue-specific eXpress output was extracted and combined for DEG detection between tissues. A universal identifier was assigned to each ortholog group and then gene counts for each species-specific ortholog within a group was assigned to that group. For species-specific singletons, gene expression was arbitrarily set to 0 for other two species. False discovery rate (FDR) of padj < 0.001, and a log2 fold change > 1 were set as thresholds for significantly different expressions. All identified DEGs by both edgeR and DESeq2 shared between *R. elegans* and *R. speciosa* were further selected for studying their model of inheritability based on their expression abundance in the hybrid compared to its two parents as shown in Fig. 3b.

### Inheritance classifications

DEGs were classified in one of four ways following the schematic in Fig. 3b: (1) F1a (additive; hybrid value = MPV; allelic effect is semi-dominance), F1b (non-additive; hybrid value = P1 or P2; allelic effect is complete dominance), F1c (non-additive; hybrid value ≠ P1, P2, or MPV), and F1d (transgressive; hybrid value > P2 or < P1, termed heterosis when favored by selection), where P1 refers to trait values of parent 1 and P2 refers to trait values of parent 2. This corresponds roughly to prior definitions of transgressive expression in studies comparing transcriptomes from F_1_ hybrids to parents [9].

### Gene-set/pathway analysis

We used the R package GAGE [71] for gene enrichment analyses. A custom database was built based on Gene Ontology (GO) annotation obtained from the Trinotate output. Functions described by GO terms were classified along three aspects: Molecular Function (MF), Cellular Component (CC), and Biological Process [72]. For each category (analyses conducted separately), tissue-specific reads from the same species were pooled together and two sets of comparisons were conducted: *R. speciosa* vs. hybrid and *R. elegans* vs. hybrid (hybrid used as the reference in both). For each category, expression levels of unigenes of the top five shared pathways were plotted using the R package Beeswarm (https://github.com/aroneklund/beeswarm). Expression patterns of DEGs within a pathway were further examined by comparing expression patterns of two parental species to those of the hybrid. To visualize the expression pattern of identified DEGs between two parents, upregulated DEGs were colored blue and downregulated DEGs were colored red for DEGs between *R. speciosa* and the hybrid, while for the comparison between *R. elegans* and the hybrid, DEGs were colored as they were in the comparison between *R. speciosa* and the hybrid regardless their expression levels.

### Analysis of anthocyanin structural elements

We characterized copy number and expression levels of the eight core structural genes of the ABP: chalcone synthase (CHS), chalcone isomerase (CHI), flavanone 3-hydroxylase (F3H), flavonoid 3'-hydroxylase (F3'H), flavonoid 3'5'-hydroxylase (F3'5'H) dihydroflavonol 4-reductase (DFR), anythocyanidin synthase (ANS), and UDP-glucose flavonoid glucosyl transferase (UFGT). In this study, the term 'copy' is used to refer to different paralogous of a given locus, and CD-HIT [73] was used to identify allelic sequence and retrieve non-redundant copies (at a 99% similarity cutoff). These downstream enzymes constitute the three primary branches of the ABP whose products differ in the degree of hydroxylation and give rise to, from shorter to longer wavelength reflections, pelargonidins (red to orange in color), cyanidins (light to dark pink), and delphinidins (purple to blue).

### Characterization MYB type transcription factors

All genes contain Myb DNA binding domain according to Trinotate annotation were further analyzed using “CD-Search Tool” at http://www.ncbi.nlm.nih.gov/Structure/bwrpsb/bwrpsb.cgi [74] and an in-house PERL script to count the number of Myb domains present and remove any gene contain ARID, Response_reg and SNF2_N domains. Genes with at least two Mybs domains were subjected for downstream analysis. *Arabidopsis* MYB3R and R2R3 type MYB genes were downloaded from TAIR database (https://www.arabidopsis.org/browse/genefamily/MYB.jsp) [75] and serve as reference sequences for function annotation. Sequence alignment was conducted using the ClustalW module available within MEGA6 [76]. Resulting alignments were truncated to exclude regions of extremely high sequence divergence (namely: autapomorphic divergence), which can interfere with phylogenetic signal owing to phenomena such as Long Branch Attraction. Phylogenetic histories were inferred under Maximum Likelihood using MEGA6 [76] and the most appropriate model of amino acid substitution based on the Akaike Information criterion (AIC) implemented in ProtTest v2.4 [77]. Branch support was assessed using 100 ML bootstrap replicate.

## Additional files

Additional file 1: Figure S1. Leaf phenotypes of *Ruellia elegans* maternal parent (top), *R. speciosa* paternal parent (middle), and F_1_ hybrid (bottom). Hybrid plants share more features in common with the paternal parent. (JPG 13817 kb)
Additional file 2: Figure S2. Sequence alignment of two copies of F3’5’H identified in *R. speciosa* and the hybrid. The F3’5’H protein sequence of *Penstemon spectabilis*, which is known to be functional [27], was used as a reference to determine protein integrity. (JPG 1103 kb)
Additional file 3: Table S1. The sequences of all identified RMYB genes in *R. speciosa*. (PDF 295 kb)

## Abbreviations

ABP: Anthocyanin Biosynthesis Pathway
AIC: Akaike Information Criterion
ANS: Anthocyanidin synthase
BP: Biological Process
CC: Cell Component
CHI: Chalcone isomerase
CHS: Chalcone synthase
DEG: Differential Gene Expression
DFR: Dihydroflavonol 4-reductase
DHM: Dihydromyricetin
F3’5’H: Flavonoid 3',5'-hydroxylase
F3H: Flavanone 3-hydroxylase
F3'H: Flavonoid 3'-hydroxylase
FDR: False Discovery Rate
FPKM: Fragments Per Kilobase of transcript per Million mapped reads
GO: Gene Ontology
HPLC: High Performance Liquid Chromatography
MF: Molecular Function
ML: Maximum Likelihood
MPV: Mid-parent value
UFGT: UDP-glucose Flavonoid Glucosyl Transferase

## References

[1] Anderson E, Stebbins Jr G. Hybridization as an evolutionary stimulus. Evolution. 1954;8:378–388.

[2] Arnold ML (2016). Anderson's and Stebbins' Prophecy Comes True: Genetic Exchange in Fluctuating Environments. Syst Botany.

[3] Grant V (1981). Plant speciation.

[4] Hegarty MJ, Hiscock SJ (2005). Hybrid speciation in plants: new insights from molecular studies. New Phytol.

[5] Mallet J (2007). Hybrid speciation. Nature.

[6] Mallet J (2005). Hybridization as an invasion of the genome. Trends Ecol Evolut.

[7] Rieseberg LH, Widmer A, Arntz AM, Burke B (2003). The genetic architecture necessary for transgressive segregation is common in both natural and domesticated populations. Phil Trans R Soc B.

[8] Parsons KJ, Son YH, Albertson RC (2011). Hybridization promotes evolvability in African cichlids: connections between transgressive segregation and phenotypic integration. J Evol Biol.

[9] Rowe HC, Rieseberg LH (2013). Genome-scale transcriptional analyses of first-generation interspecific sunflower hybrids reveals broad regulatory compatibility. BMC Genomics.

[10] Bell GD, Kane NC, Rieseberg LH, Adams KL (2013). RNA-seq analysis of allele-specific expression, hybrid effects, and regulatory divergence in hybrids compared with their parents from natural populations. Genome Biol Evol.

[11] Lexer C, Lai Z, Rieseberg LH (2004). Candidate gene polymorphisms associated with salt tolerance in wild sunflower hybrids: implications for the origin of Helianthus paradoxus, a diploid hybrid species. New Phytol.

[12] Dittrich-Reed DR, Fitzpatrick BM (2013). Transgressive hybrids as hopeful monsters. J Evol Biol.

[13] Lewontin R, Birch L. Hybridization as a source of variation for adaptation to new environments. Evolution.1966;20:315–336. 10.1111/j.1558-5646.1966.tb03369.x 28562982

[14] Rieseberg LH, Archer MA, Wayne RK (1999). Transgressive segregation, adaptation and speciation. Heredity.

[15] Schemske DW, Bradshaw H (1999). Pollinator preference and the evolution of floral traits in monkeyflowers (*Mimulus*). Proc Natl Acad Sci.

[16] Wessinger CA, Rausher MD (2012). Lessons from flower colour evolution on targets of selection. J Exp Bot.

[17] Grotewold E (2006). The genetics and biochemistry of floral pigments. Annu Rev Plant Biol.

[18] Yoshida K, Mori M, Kondo T (2009). Blue flower color development by anthocyanins: from chemical structure to cell physiology. Nat Prod Rep.

[19] Steyn W, Wand S, Holcroft D, Jacobs G (2002). Anthocyanins in vegetative tissues: a proposed unified function in photoprotection. New Phytol.

[20] Zufall RA, Rausher MD (2004). Genetic changes associated with floral adaptation restrict future evolutionary potential. Nature.

[21] Whittall JB, Voelckel C, Kliebenstein DJ, Hodges SA (2006). Convergence, constraint and the role of gene expression during adaptive radiation: floral anthocyanins in Aquilegia. Mol Ecol.

[22] Rausher MD (2008). Evolutionary transitions in floral color. Int J Plant Sci.

[23] Toleno DM, Durbin ML, Lundy KE, Clegg MT (2010). Extensive evolutionary rate variation in floral color determining genes in the genus Ipomoea. Plant Spec Biol.

[24] Streisfeld MA, Rausher MD (2011). Population genetics, pleiotropy, and the preferential fixation of mutations during adaptive evolution. Evolution.

[25] Smith SD, Rausher MD (2011). Gene loss and parallel evolution contribute to species difference in flower color. Mol Biol Evol.

[26] Sobel JM, Streisfeld MA (2013). Flower color as a model system for studies of plant evo-devo. Front Plant Sci.

[27] Wessinger CA, Rausher MD (2015). Ecological transition predictably associated with gene degeneration. Mol Biol Evol.

[28] Brothers AN, Barb JG, Ballerini ES, Drury DW, Knapp SJ, Arnold ML. Genetic architecture of floral traits in *Iris hexagona* and *Iris fulva*. J Hered. 2013;104:853–861. 10.1093/jhered/est059 24078678

[29] Vereecken NJ, Cozzolino S, Schiestl FP (2010). Hybrid floral scent novelty drives pollinator shift in sexually deceptive orchids. BMC J Evol Biol.

[30] McCarthy EW, Arnold SE, Chittka L, Le Comber SC, Verity R, Dodsworth S, Knapp S, Kelly LJ, Chase MW, Baldwin IT (2015). The effect of polyploidy and hybridization on the evolution of floral colour in *Nicotiana* (Solanaceae). Ann Bot.

[31] Tripp EA, McDade LA (2014). A rich fossil record yields calibrated phylogeny for Acanthaceae (Lamiales) and evidence for marked biases in timing and directionality of intercontinental disjunctions. Syst Biol.

[32] Zhuang Y, Tripp EA. The draft genome of *Ruellia speciosa* (Beautiful Wild Petunia: Acanthaceae). DNA Res. In press. 10.1093/dnares/dsw054 PMC5397612 28431014

[33] Chow K-S, Ghazali A-K, Hoh C-C, Mohd-Zainuddin Z (2014). RNA sequencing read depth requirement for optimal transcriptome coverage in *Hevea brasiliensis*. BMC Res Notes.

[34] Paschold A, Jia Y, Marcon C, Lund S, Larson NB, Yeh C-T, Ossowski S, Lanz C, Nettleton D, Schnable PS (2012). Complementation contributes to transcriptome complexity in maize (*Zea mays* L.) hybrids relative to their inbred parents. Genome Res.

[35] Ranz JM, Namgyal K, Gibson G, Hartl DL (2004). Anomalies in the expression profile of interspecific hybrids of *Drosophila melanogaster* and *Drosophila simulans*. Genome Res.

[36] Laurent JM, Vogel C, Kwon T, Craig SA, Boutz DR, Huse HK, Nozue K, Walia H, Whiteley M, Ronald PC (2010). Protein abundances are more conserved than mRNA abundances across diverse taxa. Proteomics.

[37] McManus CJ, May GE, Spealman P, Shteyman A (2014). Ribosome profiling reveals post-transcriptional buffering of divergent gene expression in *yeast*. Genome Res.

[38] McClintock B (1993). The significance of responses of the genome to challenge.

[39] Chen ZJ (2010). Molecular mechanisms of polyploidy and hybrid vigor. Trends Plant Sci.

[40] Wu Y, Sun Y, Shen K, Sun S, Wang J, Jiang T, Cao S, Josiah SM, Pang J, Lin X (2015). Immediate Genetic and Epigenetic Changes in F1 Hybrids Parented by Species with Divergent Genomes in the Rice Genus (*Oryza*). PLoS One.

[41] Chen ZJ (2007). Genetic and epigenetic mechanisms for gene expression and phenotypic variation in plant polyploids. Annu Rev Plant Biol.

[42] Springob K, Nakajima J-i, Yamazaki M, Saito K (2003). Recent advances in the biosynthesis and accumulation of anthocyanins. Nat Prod Rep.

[43] Shimada N, Sasaki R, Sato S, Kaneko T, Tabata S, Aoki T, Ayabe S-i (2005). A comprehensive analysis of six dihydroflavonol 4-reductases encoded by a gene cluster of the Lotus japonicus genome. J Exp Bot.

[44] Wei H, Chen X, Zong X, Shu H, Gao D, Liu Q (2015). Comparative transcriptome analysis of genes involved in anthocyanin biosynthesis in the red and yellow fruits of sweet cherry (Prunus avium L.). PLoS One.

[45] Zhang Y, Xu S, Cheng Y, Ya H, Han J. Transcriptome analysis and anthocyanin-related genes in red leaf lettuce. Genet Mol Res. 2016;15(1):gmr7023. 10.4238/gmr.15017023 26909931

[46] Hodges SA, Derieg NJ (2009). Adaptive radiations: From field to genomic studies. Proc Natl Acad Sci.

[47] Zhang Y-H, Zhang S-D, Ling L-Z (2015). De novo transcriptome analysis to identify flavonoid biosynthesis genes in *Stellera chamaejasme*. Plant Gene.

[48] Zhao D, Jiang Y, Ning C, Meng J, Lin S, Ding W, Tao J (2014). Transcriptome sequencing of a chimaera reveals coordinated expression of anthocyanin biosynthetic genes mediating yellow formation in *herbaceous peony* (*Paeonia lactiflora* Pall.). BMC Genomics.

[49] Ahn JH, Kim J-S, Kim S, Soh HY, Shin H, Jang H, Ryu JH, Kim A, Yun K-Y, Kim S (2015). De novo transcriptome analysis to identify anthocyanin biosynthesis genes responsible for tissue-specific pigmentation in *zoysiagrass* (*Zoysia japonica* Steud.). PLoS One.

[50] Falginella L, Castellarin SD, Testolin R, Gambetta GA, Morgante M, Di Gaspero G (2010). Expansion and subfunctionalisation of flavonoid 3', 5'-hydroxylases in the grapevine lineage. BMC Genomics.

[51] Casneuf T, De Bodt S, Raes J, Maere S, Van de Peer Y (2006). Nonrandom divergence of gene expression following gene and genome duplications in the flowering plant *Arabidopsis thaliana*. Genome Biol.

[52] Tripp EA (2007). Evolutionary relationships within the species-rich genus *Ruellia* (Acanthaceae). Syst Botany.

[53] Dubos C, Stracke R, Grotewold E, Weisshaar B, Martin C, Lepiniec L (2010). MYB transcription factors in *Arabidopsis*. Trends Plant Sci.

[54] Ambawat S, Sharma P, Yadav NR, Yadav RC (2013). MYB transcription factor genes as regulators for plant responses: an overview. Physiol Mol Biol Plants.

[55] Dubos C, Le Gourrierec J, Baudry A, Huep G, Lanet E, Debeaujon I, Routaboul JM, Alboresi A, Weisshaar B, Lepiniec L (2008). MYBL2 is a new regulator of flavonoid biosynthesis in *Arabidopsis thaliana*. Plant J.

[56] Gonzalez A, Mendenhall J, Huo Y, Lloyd A (2009). TTG1 complex MYBs, MYB5 and TT2, control outer seed coat differentiation. Dev Biol.

[57] Appelhagen I, Lu GH, Huep G, Schmelzer E, Weisshaar B, Sagasser M (2011). TRANSPARENT TESTA1 interacts with R2R3‐MYB factors and affects early and late steps of flavonoid biosynthesis in the endothelium of *Arabidopsis thaliana* seeds. Plant J.

[58] Pandey A, Misra P, Bhambhani S, Bhatia C, Trivedi PK (2014). Expression of *Arabidopsis* MYB transcription factor, AtMYB111, in tobacco requires light to modulate flavonol content. Sci Rep.

[59] Soltis P (2013). Hybridization, speciation and novelty. J Evol Biol.

[60] Hegarty MJ, Barker GL, Brennan AC, Edwards KJ, Abbott RJ, Hiscock SJ (2008). Changes to gene expression associated with hybrid speciation in plants: further insights from transcriptomic studies in Senecio. Phil Trans R Soc B.

[61] Tripp EA, Manos PS (2008). Is floral specialization an evolutionary dead‐end? pollination system transitions in *Ruellia* (Acanthaceae). Evolution.

[62] Harborne J, Hall E (1964). Plant polyphenols-XIII. The systematic distribution and origin of anthocyanins containing branched trisaccharides. Phytochemistry.

[63] Bolger AM, Lohse M, Usadel B. Trimmomatic: a flexible trimmer for Illumina sequence data. Bioinformatics. 2014;30:2114–20. 10.1093/bioinformatics/btu170 PMC4103590 24695404

[64] Grabherr MG, Haas BJ, Yassour M, Levin JZ, Thompson DA, Amit I, Adiconis X, Fan L, Raychowdhury R, Zeng Q (2011). Trinity: reconstructing a full-length transcriptome without a genome from RNA-Seq data. Nat Biotechnol.

[65] Langmead B, Salzberg SL (2012). Fast gapped-read alignment with Bowtie 2. Nat Methods.

[66] Roberts A, Pachter L (2013). Streaming fragment assignment for real-time analysis of sequencing experiments. Nat Methods.

[67] Lechner M, Findeiß S, Steiner L, Marz M, Stadler PF, Prohaska SJ (2011). Proteinortho: detection of (co-) orthologs in large-scale analysis. BMC Bioinforma.

[68] Camacho C, Coulouris G, Avagyan V, Ma N, Papadopoulos J, Bealer K, Madden TL (2009). BLAST+: architecture and applications. BMC Bioinforma.

[69] Robinson MD, McCarthy DJ, Smyth GK (2010). edgeR: a Bioconductor package for differential expression analysis of digital gene expression data. Bioinformatics.

[70] Love MI, Huber W, Anders S (2014). Moderated estimation of fold change and dispersion for RNA-seq data with DESeq2. Genome Biol.

[71] Luo W, Friedman MS, Shedden K, Hankenson KD, Woolf PJ (2009). GAGE: generally applicable gene set enrichment for pathway analysis. BMC Bioinforma.

[72] Ashburner M, Ball CA, Blake JA, Botstein D, Butler H, Cherry JM, Davis AP, Dolinski K, Dwight SS, Eppig JT (2000). Gene Ontology: tool for the unification of biology. Nat Genet.

[73] Li W, Godzik A (2006). Cd-hit: a fast program for clustering and comparing large sets of protein or nucleotide sequences. Bioinformatics.

[74] Marchler-Bauer A, Bryant SH (2004). CD-Search: protein domain annotations on the fly. Nucleic Acids Res.

[75] Huala E, Dickerman AW, Garcia-Hernandez M, Weems D, Reiser L, LaFond F, Hanley D, Kiphart D, Zhuang M, Huang W (2001). The *Arabidopsis* Information Resource (TAIR): a comprehensive database and web-based information retrieval, analysis, and visualization system for a model plant. Nucleic Acids Res.

[76] Tamura K, Stecher G, Peterson D, Filipski A, Kumar S (2013). MEGA6: molecular evolutionary genetics analysis version 6.0.. Mol Biol Evol.

[77] Darriba D, Taboada GL, Doallo R, Posada D (2011). ProtTest 3: fast selection of best-fit models of protein evolution. Bioinformatics.

