# Supplementary figures and images for: Genome-scale transcriptional study of hybrid effects and regulatory divergence in an F1 hybrid Ruellia (Wild Petunias: Acanthaceae) and its parents
Source: BMC Plant Biol. 2017 Jan 17;17:15. doi: 10.1186/s12870-016-0962-6 (PMC5240417; doi:10.1186/s12870-016-0962-6)

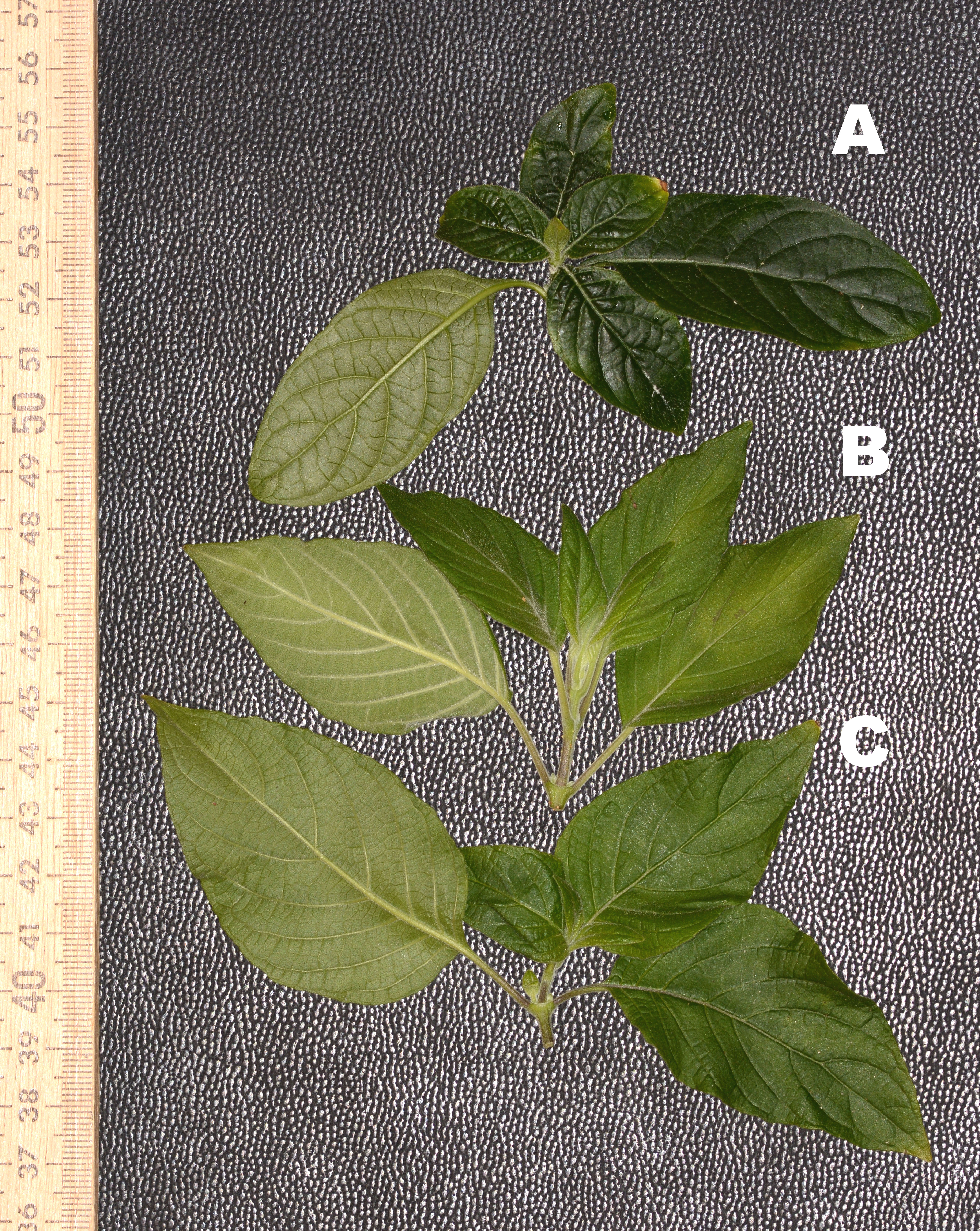

Supplement: Additional file 1: Figure S1. — Leaf phenotypes of Ruellia elegans maternal parent (top), R. speciosa paternal parent (middle), and F1 hybrid (bottom). Hybrid plants share more features in common with the paternal parent. (JPG 13817 kb) [file 12870_2016_962_MOESM1_ESM.jpg]

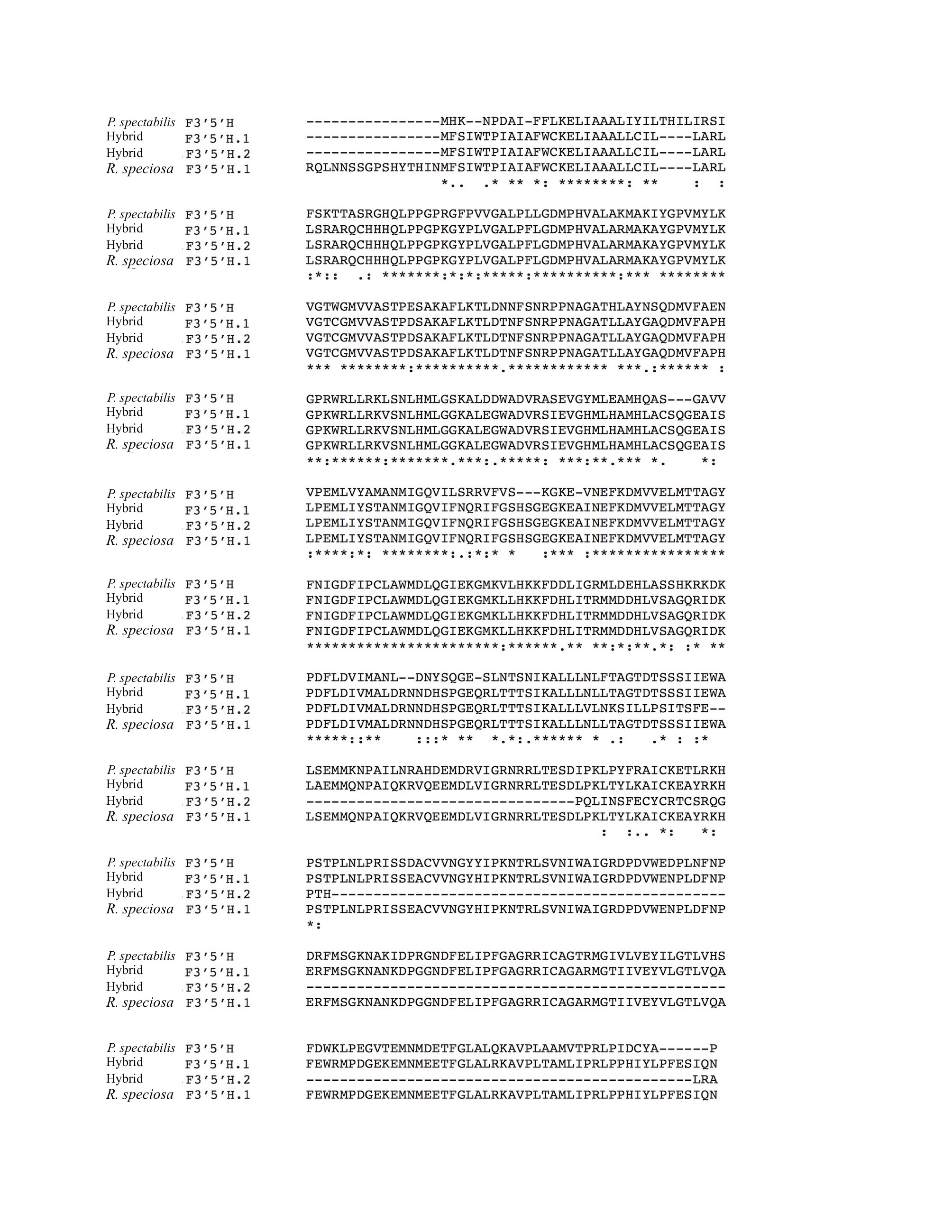

Supplement: Additional file 2: Figure S2. — Sequence alignment of two copies of F3’5’H identified in R. speciosa and the hybrid. The F3’5’H protein sequence of Penstemon spectabilis, which is known to be functional [27], was used as a reference to determine protein integrity. (JPG 1103 kb) [file 12870_2016_962_MOESM2_ESM.jpg]
