# Supplementary material for: Genome-scale transcriptional study of hybrid effects and regulatory divergence in an F1 hybrid Ruellia (Wild Petunias: Acanthaceae) and its parents
Source: BMC Plant Biol. 2017 Jan 17;17:15. doi: 10.1186/s12870-016-0962-6 (PMC5240417; doi:10.1186/s12870-016-0962-6)
Supplement: Additional file 3: Table S1. — The sequences of all identified RMYB genes in R. speciosa. (PDF 295 kb) [file 12870_2016_962_MOESM3_ESM.pdf]

Supplementary Table S1. the sequences of all identified RMYB genes.
Part I. the nucleotide sequences of RMYB gene family.

>RMYB1
GACATTCTAACATCCTCAGTTTTTAGTACCAGGCAAGTGGCTCAAGTTTTGAACAGTGTT
CAATGAATAAAAAGTTAAAAGCACACCCACCTTCACCTTGATGGTCAGCAAGAAAACAAA
AGCTATACAAAAACACGCATAAATTACATAATTTATGTATTATTTACAGCTATAATGTAA
TCTATCTATCTAACGAATAATGAGTTTTCTTCAGCCCCATTCTATCAGATCAGGATCACA
ACCACTAGCATAATCAAACACAATTCTACTGATTCACAAGCTATCAGTTGTTACGTGTAA
TTCAAAAACATCAAATTATCTTGGCATGGGAAAGCTACAACTTTCAAAACCCCCACGGCC
GTCATGACTAGGAAGGTTGGTTGGAGCAGGCATCGGCAAGCTATCCATGGCAGTTGTGAT
ATCATGAATGCTGGACCTCTTTCGCTCTTTCTTCCCTGAGGTGTGACGGAGAAAATATTT
TTGAGCATGGCTAGCCACTTGTGTTGGAGTTCTTGTGACCACTACATTCCTTGAAATGCT
TCTCCAATCACCTTTGCCATATCTATCCAGTCCAATTAAAAACAACCTGTGCTCATCCTC
CGTCCAAGGTGTCCCCTTTTTCCTCTCAGCATCGCGGCTCCGACTGCCGGCGTTGGGTGA
TCCGAATGAAATCTGACCCGAACTACTGGAACCTCGTTCGCACTCAATTGATGTCGAACC
ATCCGAATAACTCGGCAGCTCAACCCGGCCCGAATCAATCTCGGTTATATCGTGAAGAAG
GGCTTCATAATGCGCTCTGACATCATCCGGCGATTTGCCAGGGAGAAATCCGGCGATTCT
CTGCCAGCGGTCGTCAATCCCGTCCGGGAATTCAACCAAAGCCTTCTCGAAAATTTTGTC
CTCCTGACGGTTCCATGGTGGGGCGGGGGAATCAGGCATGTGCATCCACCGATTGCAGGC
GTAGATTTCCATTAAATTGGTGGGTTTTTGGACTTGGTTTCTTGAGGGGGTTTTGTTTGG
TGGAAGAGGTTTTGGAATTGGGGACTAGATTGTTCGAGGGAGGGTTTGTAATAATCTGTG
TGTAGTGTTGTGTAAGAACACACAAGGTTAGTGAGATGTTTAATTATGAGGGCCGAATGA
AGCACCAGCCCGTTATCCTTCCACTGCTG

>RMYB2
TCCACCACTATTTATTTTAGTTAAATTTACAGAGAGTCACCACATGTTTATCAAACTTTA
ACGATCAGGTTTGGGAACTCAACATTTATGTCCATCGGATAACTGTTGTTTAGGATTAGG
GTTTAATTTCCATTCTGTCTCAATAGTTTTTCTTATTCCCAAACATGACATAAAGTCAAT
AACCATATTTGCCACAAAAATGTAACCCAAAAATTTCGCAACACATCATATATAGTGAAT
GACATACATTCAATAATATAATAATAATAATAATCCAGTCTTTAACATTTACACTCC
ATGCATGCAATCAACAAAAATAAAATTAAAGGAAAAAAAAAAGAAAAAACAGAAGCACAA
TTAATTAATTACTATGCCCCCTTGACAATCCTTATTACCTTTTTTTTCGGATTTTAATAA
TAAAGCTCTCTATTTCACACCCTACCCCTCCCTATTATATTTATTTTATTGTGCCAAAAT
AATAAATAATATAAAATTAACCTTCGGTAAAAACGAAAAACTAACCACTAGCTTCTGTAA
CTTTTTCACTCTTTTTTGACATTTTTTTCACCGCCGCAGCCGCTGTCGTCGGACCCATCT
CCGTTATATCCGCCCACTTGCTTCCTGCAGAACAATATCACGATTCCTGCGCGGCGTTGG
CCACCGATGAGTGCTGCTTGTTCGCGAGAATTTCGAAACCCAATCCCTCGTCATCGTCGT
CATCGTCGTCCTCGTCGTTGATGTTGTGATCGTCGCTTTCGTTGTCGCGATTGTCCTGAT
CCTGCATTGCGGAATCGGGGAGTTGTTGTCTGTGTAGATTGGGAATTGGCCACTGCTGCT
CCGGCTGCGCCATTATTGGCACCATGGGGTTGTTATTGTTGACCTCCTTGGCTCTTTCTT
TGTATATGGCTTCCAGCTGGTGGAAGTAAGGGCACGTCTTGGCGTCATCCGGCCGCTTCT
TGTTGCTTTCCTTCACCTTCTTGAAGTACTTGTTGATATTCTCCCATTTCTCCTTGCAAC
GCTTCGAACTTCTGTTGTATCCAAGCTTCGCCATGGCGGACGAAATTTCCTCCCAGAGAG
GGCCTTTTGGCCCGTTATCTTGGTATTTTAAATCCATGCTTGTTCGTAGATTAATTAGCG
CTTGAACTTCGGCTTTTGGCCACCGTGATGAACTCGCGGCCGGGGAATATCCGGCCACTT
CTCCGCCGTTGTTGTCCGTTTTTGAATTGTTAAATACTTTCGCCGGTGGTGGCACTGTAA
TTGGAGCTGGCGCCGGTTGTGTCGTTGGAGCTGGCGGGGCTGGTGGAGGCTCTGGTTGCT
GCGGTGGCGGAGGAGGCGGCTCCGCCGCCGCCGCCACCGGCGGGATCTGCAAATTGTGTT
GATCAGTTACTTTTTGTAAAAATGCAATCACGGCGGCGTCTTTAGCGGCGGCGATGGATC
TCTCCTGGACTAATAGATCATGTTCGCGATTAACTCTGGCGATTTCCTGAACGCGCCAAG
CTTCTTCCCTCGCCATTCTTTCGCGCTCGCGTTTCTCCAGTGAATCCAGGAATTTCTTCT

GCAATTCCTCCTGTTTCTGAATAACGTCTCGCACCAGCCTGTGGAAACAATCCTTCCATT
TCCTCTTCCTTCCACGCCGTCTTTGGATATCCTCGTCGGACGAAGTCGACGACGACGTCG
AATCCGACATCAGCCCCGGGCGCGATGCCTGAAAGTTGAGCCCTTGCGGAGGAACCGAGC
TGTTCCTCAGATGCTGCATTAGCGGCGGCTGCGGCGGCGGCTGCGCGATTAATGGAGAAG
AATCGATAGGGATTGAGGCGGCTGCCTGGGGAGGGACCACGCTTAAAGGCGTCGGACTAA
TTGATGAAACAGTAACATGGGCTGGCATTGTCGAAAGGGAAGCTGTGGCGGCGGGGCGCG
GCGGGGTGAAAGGATTAGGCGGCGGAGCGTTCTCCAAGGCTTCCAGTTGATCGAAGAATC
GGTAGGTTTTGCCGTCGGATTTTGCGGCGCGGCCGTCTTTGGTTCGCTTGTGGTATTTGT
ACACGTTCTCGAATTTTTCCCGGCATT
>RMYB3
TGCTTGGCTATTGATGCTTTTGTTGCTGTTGCTGTTGCTGTTGCTGCTGCTGCTCCCTTT
CTTCGAGTCGAGACTCATGATGTAGTCCTGCAAAGCAGAAGTAGGTATCGGAGACTTATT
CTTGCATCTTCCATCTGTAAGTTTCCTCCTCTTTGTTGTGTTCCAGTGATTCTTGATGGT
ATTCTCAGTCCTCCCGTGAAGCCTTTTCGCAATTTCTGCCCATCTGTTCCCGAGCTCTGC
ATGAGCCTCGATGAGTTTCTTGTCTTCCTCCTCCGTCCAATCATCTCTTTTTATATCAGG
CTGCAAATGATTTCTCCATCTCTCTCGGCATTGCTTGCCCGTCCTCCCCTTCAGCTTTTG
CCCCATGAACGTCCATCTTTTTGCTCCATAAATCTTCACCAGATTGACCAACAATCTGTC
CTCTTCGAGGGTCCACAATCCTTTGACAGGTTTCGACACATTTGAGCCTCCGTCCGAATT
GTCAGCGTTACAACTTTTTCTTGTATCTGTGCTATTTCCTTTTAAACAGCCAAAAGCATC
AGCACTCACAAAAGAACTTTCATCTGTGTTCACATACTCAAGTCGCATAAAATCAGGGTA
GTTCAATGCTGCCTCAATTCCATTCAATGAATCTACCTCATTTGGGAAACTCAAGAACCC
ATCACCCTTGAAATTCTGCATGTTCATATCAGTGATCTGGTTCCCCTCTCCTTGGATGTG
CTGCATGACCGCGCTAGAGTAGCCATTTCCCTCATAAGGCATGTACTTGTTTAGAAAGGG
ATCAAAAGGATCAATGGGATTAGATTGCCTATCGATGATTGAACTTAAAGCCAATTCATC
AAGATGAAGGATGTCTTGATTAGATGACAAGGGATTGCTAGAGCAAATATTATGAATGGG
GAAGGATGCAGGGAGAGGGACATTTTGGTCATTTTCAAACGGTGTGTTGTAGGACTCCAT
TGGAGAGCGATAGAAGTAATGAGTGACAAGAAAGGGAATGGAGTAAATCCCTGACTTAGG
CCTTGGTTAAGTTCATATTTATTAAGAGAAAATATAAATGCACTAAATGTGTTTCGAGTA
CATCTTGGCCGTCGATTTGTGGGAGGTGATCCGAGGATCCGAAGTTCATGTTATAGGGAT
AAGATTAGGTAGGGAGACTTGAAAAAAAAAGTCGTGTTACAGAAATCAAAATTCTTTAATC
ATTACTCTTACAATTCCGTAATTTTATTTTCTTCGAGACACATGAAATCCAAGTAGTGAA
TTTTCTCAATAAGTCAATTGTTTTCTGCTTTTAAAATATGGCCACAAGAAAAGAAAAGGT
GCACTTTGTTAACATGAGTAATCCTCACTCTTTTTTAAGCCATTCTTCAACTTACTCATA
TTCGGGGGTATAACATTCTCACCCATTTGAGAACGCAATATTCTCGTTGTGCAAGAGGCC
TTGAATTTTTGCCTTGTTGCATTAATTGACTGGTATTTCCAGCATAGTGCATGTTGCGGG
TTTGCTTTGATATCATATGTATCCCGCCAAGGAGGGAGAAGTGATTATTAGCCTTTTGGG
ATTGAAAATTGGCCCCACAAATCAACACGAGTCGTTTTTTGTTCACCTATCGCACAACTA
CTTGAAGGCCAACATGATTAACCACATAGT
>RMYB4
GCCAGGCCAAGAAATTCAAATTTGAACAAAACAGGAAAAAAAAAAAAAGTGCAATTTTCTA
CAGTATAATCTCATTCTCATTCCCATCTTCCAAATTTTTGATTTGGCAAAACAAGATCAT
TCTACATAAAAAAGGAAAGGAAAAAAGAAACCACAATTTCAAGACAGAAAACCCAAATTT
TGGGGATTTAACTCACCAGCATTTTTCAAAAATTTGGATTTTCTGTCCAAAATCGTGTAC
CAAACCAAGAAAATTAAGTGCCGAGAATCGAGAATGAGCTTATACTTATACCCCCAAAAA
TTGCAAATATTTCTCCAAACCTCATACCCATATCATATAATAATTCCCAATTTCCATACC
AAAATGGAAAAAACCCCAAATTTAAAATCCTAACAATTCAGGAATCAAACAAACCCTGGA
TGCCTTGATCATCCAACAACCACTTCTCCAGGGCCAGCACGCCCTCCGGGTCGGGTTTGG
GAGACCCAGATCTTGAGAAATCATCCGACGACAGGGAAGAATACGACTCAAAGGATTCAA
ATCCAAACAATCCCTCCCCTAAATCAGCTCCACCAGCGACGACTTCTTCGGTGCTGCAGC
TGGAATAATTATAATTCGAAAACTCCTCACATTTGGGGGTTTTGTTGGCCCAATCCTTGA
GTAATCTTGCGATGTTCTCAGTGCTGGATGCGTACGACGTTGTCTGAATGGGGTTCGGGG

CGAAATTAGGGTTTGTCATGACATCCTCATCGATGGATTCCGAGGTTGAAACTGAGAGAG
CATTGGCAAGGGCTTGTTTGGCTGTGTGGATATCCGTTTGCAGCTTTCTCTCCCACTGGC
CACGCGAAATGGGTTTGGAGGAGCCGTTCTCCGTGGGCCGGTTGCCCGACCCGGGTTGGA
TCCGGTCCAGCTTCTTCTTCAAGTGAGTGTTCCAGAAGTTTTTGATGTCGTTGTCGGTTC
TTTCTGGGAGGTAAGAAGCTATGGCAGCCCATTTGTTGCCTAGCAGGGCTTGAAGCTGGA
TAATCATTTTCTCTTCATGATCAGTAAAGGTCCCTCTTTTGATTCCAGGCCTGAGGTAAT
TAGTCCATCTCAGTCTGCAACTTTTGCTGCATCTTTTCAACCCTGTATTGGGAGGAACAG
ACCTCCAATTTCCAGGACCATGCTCCTGAACATAGGACACCAACATGATGTCTTCTTCAG
GAGTCCAAGGGCCTTTCTTCACCCCCAGTTTATCACAGCAAGGTGGCCTCCCCATCAGAA
TTCAATTCAATTTCTTTCAATTTGGGGAAATTAGGGTTTCCTTGATTTCTCACTCTTGGC
TGTATTTTTTTTATTTTTTCAAAAAACAGGAGTTCTTGGGAAGGGATGAGGATGAGACAA
TGGTCGTTTGGGATCTGGATATATATATATATATATTGTGGGGGGGAGGGGCTGACCTAA
TTAATTATTTAATTAACTAGTGAAGGAGGTGAGGAGTAGAGAGAGGAGTCAAAATGCAAT
AAATTATGAATGAGAGTTGATGTTATTTCCTTTCCATTCCAGACTCCATGTCCCAATGGA
TGAGGATTGAGGAAGAAGGATTCTTGCTTGCTTTATGGTAGCTATCTCTCTCAGTCTCTC
AGGGCTACAAAAGTCAGCTCAGATCTCAGGACACATACAGCAGTGGAGTGTGTGTTGCTT
TGTTTGTCTTCG
>RMYB5
CTGGATAGAGAGGTAAGCCCTGTCGCTGTCTTCTTTTGATTCTAGTGTTCCAATAGTTCT
TGATTTCATTGTCCGTACGTCCAGGTAACTCAGCAGCCATTCGAGCCCATTTATTTCCTA
GCGTGGCGTGAAGTTCAATTATACGGCGCTCCTCCTCAGGAGTGAAGGCGCCTTTCTTAA
GATCTGGTCTAAGGTGATTTGCCCACCTCAAACGGCAACTCTTTCCACATCGAGCAAGCC
CGGAGTGTTTTTGAACTGCATTCCAGTTGCCCTCCCCGTGCTTGTTAACATATTCAATTA
AGATTGCATCTTCTGCTGAAGTCCACGGGCCTTTCTTAAGTGGACCATTTGGGCCCGTTG
TTGTTGTTCCTCCACTGTTGGCATCATCAACAGAAGGTGAATCGACACCGCTGCCTGCCA
TTCTTTCATCACTTTCGCTTGTTATACTCATCTCCTTTATCGTGAATCTTCCCCTGAAAC
CTAACAAATCTGCCCCTCAACAAAAAGCAAAATAAAAAAATCGTTTGAACAAAAATTGGA
AAAAGAAAATAATGCTAATTCTCTCAGGGGAGAATCAGACAATGGAAATTTTAGAAACCA
TGGGCAAATTTTCTGTGGGCCAAAATTACACTCGCGAACACGACAAGAAACAGCAACACC
AGCCGAAATTTATCGAAGGCCACACAAGAAAAAATCTGGAACCAAAAAATGGATTCAAAA
CACTGAAAAACAGAAT
>RMYB6
GGCCGCGCCGAGGAGCGATCCTTGGCCGCCCCGGCCACAGTGGCCGCCCGCTTTCTGGGT
GGGAGTGGGGTGGGTGGGTATGTGCACAATGATCATTTTTAAGAATACGCATATAAATTG
AGGGTTAGGTACAGTACTTTAGATTTTCCAAACTATAGAAGCTGCAAATATTCATCATAG
ACTTTTTCCAGCATTTCTACATCGTCGGAGATGGATTCTGAGTGGGTGAGCACCGGAATC
AAAGCACATTCCCCGCCGTCGAGAAAATGGGCTTCTTCACCGTCACCATTGTACACTCCA
AAGGTAGCATCCTCTATTTCAAGCAGTGCCGGCCACTCCCTCCGTACATCCTCCGCTGCC
TTGTCACTGCCACAAGACGTGCCACTGGCCAAGCTGTTGTAGCTGCCGGTACAGGAGAGT
TCAGAGCTGACTCTTATCGGCTTCGGAAAATACACTCGAGTCTTGTTTGGAGTATCATTC
TCCTTTTCTTTCCTGGCAGTCAATGCATCATTGTTGTGGCCTCCGGGGTCCAGTATCTTC
TTTTGCTTCTGCTTCCGATCTTGGTTTTGTTTTTGTTCTATTGATTCGAGTTTGGACAGC
TCCCTGGGGGGAATGGCGAGGGTTATCGCAGCGGCACCTTTCTTGTGGAGGTGCGTGTTC
CAGTAGTTCTTGATCTCATTATCTGTTCTACCCGGCAACCTGCCGGCGATTAGGGACCAT
CGATTTCCGAGGAGGCGGTGCAACCGTAGGATGAGATCTTCTTCATCTTCGGAGATGTTT
CCTCTTATGATTCCCGGACGGAGATAATTCATCCACCTCAATCTGCAGCTCCTCCCACAC
CTCAATAGTCCGGCTTTATTGGGGAGGGATCTCCATTGGCCCTCACCATGCTGTTGAATA
AAATTGGTGAGCAAAGAGTCTTCCATGGCAGACCATGCGCCTCTGTTCAATCCCACTTTG
GAGCAGCAAGGAGCCCTTCCCATTTCTAGATTTCTAAGGAATTAGAGATGGGAGTGAAGG
CACTGCATACAGCCTC
>RMYB7

ACTAATTTTGTTAATGTTAATGTTAATTTTAATTATTATTATTATTTTAAGATCAAATTA
TTAAAAATAATATAGTATTATAATTATTAAACCAGCTAATATGCATGTAAATGGAAAACA
AAAAATGAAGTACGTCCTAAATGAACAGAAATGTAATGTCACACTCCCATAGACAATGTA
CAGTGTACAGTCATAATATTAGTAATCTATAATTTGATATATCCACATATACTCTTTAAA
ATAAAATAAAATAAAATAATTCCTCATCAGTTAATAAAGTAACAATATTCAGGAATATCA
CAACGAACGCTACCACTACCATTCTCGTAAAATAATTTTATATATATAAAATATTGTTAT
TAATTATACATCATAAATTATTTATCTCTTTTTTCTTTTTTTTTTTAAAAAAAAAGGATC
TTGATCCCTCATCCCAGAATCCTCTTCGACATCATCACTTCATCTCCAAGCTTCTGTAAT
CCAACAGCCCAGTTTTCAACCCCATAAAATCATACCCTGAATTGCCCCCACGGCCGCCGC
CGCCGTCTCCGCCGCCACATCTGCAATCCTTGCTGTTCCGAATTCCCAGCCTGCAGGCGA
AGCACGAATTCGCACTGTCAGTGTCGGATCCTCCAGGCGTGGCGAATCCGTCGGCGTCCT
GGCAAGGCAAGCTGATTCTGAGCTCCAGGTTCAAATCAGGGCACCGCTGATCGGTAGCTC
CCCGTCGATCCTCATCTTTTCTGATCACAATGATTTTCTGATCTTCCGATTCTCTCGAAC
ATCCACCGGAGAACGAAATAGTCGTCGTCGTTGTTGTTTTTAGGGTTTCCCTGTCCGCCG
CCGCCTCATTCATCGGCCGGTGAGTCGTCGGATCGATTCCCCGGCTCAACAATTTCCTCC
TGATGTGCGTGTTCCAATAGTTCTTGATTTCATTATCCGTCCTCCCCGGCAGCCTCCCGG
CAATCAATGACCACTTGTTTCCAAGGAGGCTGTGGAGTTTGATAATGACCTCATCTTCCT
CCTCCGTGAAGTTGCCGCGTTTGAGGTCCGGGCGGAGGTAATTGATCCAGCGGAGGCGGC
AGCTCTTCCCGCAGCGGAGGAGGCCGGCGGCTTTGGGCAGGGAGCGCCAGCAGCCTTCGC
CGTGGGCGCGTATATAAGCGACGAGACGGTCGTCTTCTTCCTTGGTCCAAGCCCCTTTGT
TTGTATGTGCCTTTTCACAGCAAGGAGACCTTCCCATTATACCCTTCTTTCTTCTTCTTC
TTCTTCACTGTAATACTACTCTAATGTAATTATAATTCCTGTCAAGACTACACCTTTACA
CACTCTTTATGTGTATATGTATACATACATGTACATACAAGGATTTGACTGAGAGGAGGG
TGTGAGTTGGTAAGAGAGACGGAAATTTGGATCAGGATCTGATAAAGGGCCGCA
>RMYB8
GTTTTGATCATCATGTTCGTCCCATTCATCAAGAACGAACTCCCATTGTCGACACCAACT
CCGTTATTACAGCTAATGAGCTCCTTAATTTCCTCGACACTATAATTCACTTGGCAGCCA
CTGCTACTGATCAAGAGCTTGTGATCTTCAGCACCATCCCCTTTAAAATTTCCAAAATTG
CAACCCTGGAAACACGATTTTTCGGATGAACTGCAGCTGGGCTGATCCCCTCCATAGATG
AGAGAATTACAATTACAATTCGACGACGAATTGGAAATTGGGTGGAAGCTGGGAGACATG
GCCTCAAGCTCGACCCCAGTGGACAAAGTCCTAATCTGGGAACATGGGTAAAAATTACTA
TAACCACATTCATAGGATGATGGCATTTGGGGATTATTATTATTACATGGCAATAATGGA
CAAGCAGCTGCAGCAGCCTGGTGAGTATTCACCATTCCCATCAGTTTCTTCTTCAGCTTT
GTGTTCCAGTAATTCTTGATATCATTGTCCGTCCTGCCTGGTAATTGTGCTGCTATTATT
GACCATCTGCTTCCAATTCTGGCAAATAAGCTACAAATTACTCTGTCTTCATCATCAGAG
AAATCTCCATGCTTGATGTCTGGCCTGAGGTAATTAAGCCATCTCAGCCTGCAGCTCTTC
CCACATCTCTTCAAGCCAGCTTTTTGGGGGAAAGTGATCCAATTGCCACCAGTACCATTT
TTGTGTATGTAATCTTTCATTTTGGCATCTTCTTCAGGGGACCAGGGGCCTTTCTTGACA
TTGGCCTTGTCACAGCATGGAGCTCTTCCCATTTTGTGGAGAATTTGGTGAAGAGGCCAG
GGGGACTTCCAGTAATTTTGTCTTTTTATAGGGCTTAGGTTAGCTTCCTTGGCTTCGGAG
TTCGGACAAGATGGTATGGTACTCTTGGGCAGACACACGCACATG
>RMYB9
TTTTGGGATTCTCATTTTGAGTTGATGACTCAAGTAGATCACTCAAGTTGGAGTTGGAGT
TGGAGTCGGAGTTTGAGTTTGAGTTTGAGGGGGTTTTCTCCACCGTGCTGCTGCTTGTAT
TCCTGTAGCTCTCTTTCATCAGTGCAACTTTCTTCTTACACTGGCTCACAGTTTTTCCTG
GAACTGCAGCTGCAACTCGTTCCCAGCGTTGAGGAGTTTCCTTTGGAAAGGTTTTCAGAG
CTTGGACTAAACCTTTTTCTTGAGCAACAGACCATGAGTCTTGATCTGAACACAAAGCAG
TTCCATTAACAGCATTGCTGCTGTCTGACAAATTGCCAGGAACAGAAGATTGCCCCATAG
CTGCACCGGCATTAGATAAGCCCAGCCCATCCTCTCTAGTTGAGAGAGGTGAAGCAATTG
CTTGTGCAGGTTTCCTTTTCTCAAGGAAGGAGTCAAAGGCTTTAGCAGAATCAGGCTTTT
GCAAAAGAACTGTTTTTGTAGCCTTCAAAATCTCCTCTACTGATCTCCCAGAGCCAATAT

ACTCAGATATCACTTCCCACCGCCGGGAAGTTCCTTTAGGATATTTCTGCATGCCCTTTC
TCAAAAGCTCAATTTCTTCTTTACCCCAAGGCTTTTCAGCTTTGTTAGAAACCTGTCCAT
TCATATGAATGCTGGTGGAGCCATTTTGCAGTACATTCTTCTCATCTTCCTTCTTATCCT
TTGTTTTGTGATTGGATTCAAGTGTCTCTTTGAGAAGTTCAGCTCTCTCGAGACCCTCCT
TTCTTTCCAATTTTTCACACAACTTACTAAGCTGGTCTTTATCAAGTGATGAGCACAGAC
TCTCCACATCGTCATCAGTGAGATCAAGCAAACGTTGAGACAAAATAGGCGTTGAAAGTG
TTCTTAGACGGGTCCGTTCCTTCCGGAGTAATTTTTTCTCTTTTTCCTTCAATTTCTTTT
GATTTAAAGCAGCTTCAGCTGCTCTTTTTTCCTCCTCTTCTTTCCGCTTCCTTTCCTCCT
CGGCAATCCTAGCAGTTTCTTCTTCCTGCAATCTTTTGGCCATAAGTTTAGCTTCCTTCT
TTCTTTGTTTCTCAGCTTTCTCCTCCTCTTTCCTTCTTAAAATCCTAGGATCCCTTTTAT
AGGCATTATCAACAAGAGTACGTATGCGTGCACTTTCATCTTTCCGTGCCTTCTCTGACA
GTTTTAAATTTTGCCTTTCCATCCATCTCCTGTGATCTCGACCTTCAGCTTGCTCAAGGT
CATACTCATCAGCATGTGGAAATTCTCTCCAACTTTTGAAGGAATACCAGAAATTATAAA
AGCTATCCACTTCTTTGATGGGCATTTTATCATCCCCCAAAGTAGGAATAGGCTGGGTTA
CAGACCACCGCCCATTCCTCACAAATGCAGGGCCAAATACCTTGAAGAAATCCTGTGGTG
CACAATCATAAGGAATTTCATCATCAAACTCATCCGTGGAATCATAGATTCTTCTCTTCA
AAGGATCAATCAACACTTCATATGCTTCTTGAATTGCTTTAAAATGGCCCTCAATCTCAT
CCTTCTTGGCCTGCTTAGCAGTCTCAGTTTCCTCCGCAAGCAAAAGAGCAGCCTGCTTGT
CAGGATGATGCCTCAGAGCAGCCTCTCGGTAACTCTTTCTTATCTGATCTTCTGTTGCAA
GATACCTCAGATGGCTCAATCCTAGCAGGGCATAATGGTCTTGTTGCTTCGCTTCCCCAG
ATTTTTTCTTTCCCTTACTTCCATACGAATCTGATGATTCTACATAAACATGTTCCTTCT
CATTAGGAGCACCCTTTCCATCATCATCATCGCCTGTATCTGCTTCCACACAGTGGCCTA
CCAACCTAAGAGCAGCAGCATTAAACGAATGCCCAGCAGGCTCCAGGTTTAACGCCTTGA
TCGGAAGGCTATTAGAGGATACATAGATTGGTTCACCATCCACAATTTCCTCGTGGTAAG
TAATCAGCTGTATGCTTTTCCCAGAGGCCATTGCAACTAATAAGCCAACTAGGACAGGAT
CCCAGAATCCAGCAAGCTTCCGATTAATGCGGAGAGTGGAGAATCTGATCGATATTAGTT
TGGCATGAATCAATCGCGATTCCGCCTCCGCCGACGATGATGGTGATGGTGAAGGATGGA
TTAGAGCTAGGGTTTTGGTTTAGGTTTGGCTGAACGGCTTGTGCACAGGGCGAG
>RMYB10
GTTCTTGATCTCATTGTCAGTTCGCTTTGGTAAATATGCTGCCATTGCGGACCATCTATT
GCCTAACAGAGCATGTAGTCGAATAATGGTCTTCTCTTCATGGGAACTGAACTTCCCCCT
CTTGATATCAGGTCTCAAGTAGTTTGTCCATCTCAGTCTGCAGCTCTTCCCAATCCTCTC
CAACCCTGCTTTAGCGGGGAGGGACCGCCAGCTTCCATGGCCATGTTCCTGAATATAAGC
AAGCAGCTTTTGATCTTCCTCCCTTGTCCATAGCCCTTTCCTCAACCCCACTTTTCTATC
CCTACTTGGCCTCCCCATTTTCAAAATTAGGGTTTCTTTCAATTAAGGGCACAGCTTTAG
TTTACTCCAAATTCAAATTTGTGGG
>RMYB11
TTAATTAAATAGTCTCAAATTTCTCTAGAATTGACAATAATAATTATGAATGTCATTGAA
TTTAAAATCATTTCGCCTCTATTTTGTCGAATCTAAAAAAAAAATATCTTGCAATGGGTAT
AATAACATTGGTGGGCTAGTCATGAACTGAATGTGCCTCGGGGTCGTCAAATGCAGAAGC
ATCCCAAAGCCACGGCAACGGCGCCATGTTCTGGTCGTGCAGCAATCCCAACCATCCATC
GTCGTCCAACTTCCCATGATCCCATATTTCTTCAAAGGGCGAGTTAAAGTTCCATGGAAT
TGGGGACTCAATCGACCTCAAGTTCCAAAAATCATCGTCTTCAATATTAATACTACTACT
AGGAGCCGAAGAAGAAGAATATTCATATCCGGATGAAGTGCTGCAGCAATGCTCGACGAG
ATTCTCAGTTGCATTGCCATTGCAGGCTCCTCTATTCTCATCCAAATCCCCGTTGCTCCC
CTTTATAGTTGTGTCATCCATGAAATGTATTTTTGTACCCACTGCCTCATCAATTAAACA
GGTTGCACTAGTCATCGTGTTAGTATTCGTCTTCTCTTTATCATCCAATTTTCCAGACTG
TAGTAGTGTTTGGTCGTTGTTGCCTTGTTGGCGATGATCATCGTGTGTACTGATTGGTTT
GCTCGAGGATGATGCTATTACTTGTTCCTTTAAATCAGGGTTGGTTGTCCTTTTCTTACA
TGGATCATCATCAACATCCACGTCATGGTTGACATTTGTTGCTATTACGGCGGCGGGTGA
TTTGACCATGTTATTATCAATACCATGTATCTTGAGATGCTCAAGTGATGATAGCGATTG

CCGCCGCTTGCGGTGCATCGTGTTTGGCTTTGGAGGAGGCGGTGGTGGAACAAAATGTCG
AAAATAACTGTGTATTTTTCTACTCAAATGAGAGTTCCAGTAATTCTTGATCTCGTTATC
CATTCTCCCAGGTAAATACCCCGCTATTAAAGACCACCTGTTTCCCAACGAAGCATGGAG
ATTAACGATGGCGTCTTCTTCTTGAGGGGTAAAATTGCCCCTCTTCAGATCGGATCTCAA
ATAATTTATCCATCGCAGTCTGCAACTTTTTCCGCACCGTAACAACCCTGCATTCTTGGG
GAGTGATCTCCAGGCGCCTTCCCCATTGGCCTCAATATACTTTGTAAGTATTTCATCTTC
TTCTGCAGTCCACCTCCCTCTCTTCAAACCCACTTTCTCACAGCAAGGTGTTCTCCCCAT
AAAATTATTATTTTTTATTTATATTTAATTATTATTTTAGTATTTCAACAACTCAATCAC
ATCACATATGTATAGAGATAAATCCTGATGTTAGAAACAGACACATAATTAGTTATATTG
CAAGAAATGCATAATGTACGAGGGCACACACATGTATATATATATATATACTGCACAAAA
AGAATAAAAATAAAGACGAGAGAGATGTATATGTTTTGATTATTAGTAATAAAGAAGAAA
TGAAATGTGAAATGGGTT
>RMYB12
CTGGGTATTCCAATAGTTCTTTATCTCATTGTCTGTACGTCCAGGCAAATGTGCAGCCAT
CCGAGCCCATTTATTTCCCATCCTAGAATGAAGTTCAATTATTAGCCGCTCTTCTTCTGA
GGTAAATGCTCCTTTCTTCAGGTTGGGCCTTAAATGGTTGGCCCATCGTAAACGACAGCT
TTTACCACACCGTAAAAGTCCTGTGTGTTTCTGGACAGCATTCCAATTGCCTTCTCCATG
TTTTTTCACGTATTCAACCAAGATTGCATCCTCAGCAGATGTCCATGGGCCTTTCTTTAA
GATAATTCCGCCATTTGAAGTTCCACCAAAATTATCACCATCATTACTCACAGGTGAATC
AGCAGAGAGCACTCCATCATCACTGTCATTAGCAATGCTGTGGCCCATCATTCAATATGT
GCAGTGTGTGAGCGTGTGGATATCAGAAAGAGAAGAGGCAGGAACTTATACT
>RMYB13
AGCCGCAGAGGAATACGGCTACGTAACACAAGTGTTGGGTGGAAATTTGAGGTCAAGAAA
ATATTTAGAAACTAATTAACATCCAGAATATGTGTTAAAGAAATTGCGCTTCCAAGAATA
TACCGACGTTAATGCCGTTAACCTCCCGGCCGTCGAAAATATTTTTCCATCTCCTCCCTA
ACCGTCTCCTGAATATATGTACGAAGTTCACCCGAAGTAATTTCTTTTACCTCGCTGACC
CTACCCGGTAAATCCTTACTCTTGCGTTGTTCCGTCGGAATATTCGATGAAGAATATGCC
TTTTCACTGTCAGATAAACCGGTTCCCGGCAAACGAAGAGTCAACGCCGTCACCGCACGA
TCAGCCTCTTCGCTGTTGCCGTTGGCGGCGGCGCCTGCCTCCGCCTGATTTACCGCCGCC
GTAGGTTTTACAACTGGTATCAACGGAAAAAAGAAGTTCACATAATCGCCAGGCTCGGAT
TGACTCCACATCGCCAATGATACGTCTCCGATTTCCGATTGCCGCAAAACCTGCGGCGGC
CGCTCGCCAACTCCGGCTCTGGCGGCGCTCCTCGCCTCTTCGTACTTCCGCTTCAGATTG
GAATTCCAGTAATTCTTCACGGCGTTGTCGGTGCGTCCGTTGAGCATTCTCGCGATAGTC
GCCCATTTGTTGCCTAACTCAGCGTGCGCCCTCACAATAATCTCCTCTTCCTCCGGCGTG
AACGGACGGCGCTCCACCTCCGGCGAGAGCTGGTTGCACCACCGCAACCGGCATGACTTC
CCCGATCTCCCGGTGATCGACTTGCTAATCTGAGACCAATTCCTCGCGCCGTGTTTCTTC
ACGAGTTCACGGAGCAATATGTCCTCATCCTCACTCCACGGCCCCTTGATCTTGTCGGAA
ACCTCCTCGGTCGTCGCATCCGGCTCCGCCGCCATGAATCCTGGTAAAATCCCTAACCCT
CGCCGGTCCCTAATTGGAAAAAAATATCTCTCACGATATATATATGTCTCTGTGTTTTAT
GTGGTGTTCTTAAAAGCGTTAAGGACTTAAGGTAACTGAAAGAACGGTTAAAGAGGGAGG
CC
>RMYB14
GGCCCCCTATTTACAGATTAAGAATAAAAACCTCTATAGGAAGAAAAACACACATGACTT
TGCTGCACGAAAATGAATTCATCAGACCTTGGAAGGAAGGGAAGGAAGCAAGAAAGGATC
ATCAATTCATCATTAATTCATCATATAATCCCAACTAAGAAAACTGTCCGAAAGTGGGAG
CCGGATTAATTGTATGCCAATTACCAATGCTCAAGGATCCGTCCGCTGCGAATTGTCCCT
GTGCTTTGGTTTCCCCGTACATATGATCTTGTCCAGTCGGATTGTGGATAGAATTCCCCA
TCAAAAACGGGGCTTGCAGATACTCATTCCACTTGATCATATCTTCCCCAATTTCGCCGT
GGGTTGTTTTATTATCAGGTTTTCCACTCGAGTCTGCCGGCGTCCATGGGAACCCAGCGC
CGCTTTCAAATGTACTGTAGCCGCTTTGGAGCTCAGTGCTGCTTTCGAATTTTACGGTGA
AAGGCGGTAAGCTGGGATTGTCGGCAGGAAAGTTCACAGGAGGCTTATTAATTTGGGGGA

CAATCGGGGGTGAAAGGTTATTAAAAGCGGTGAATTGGTGAGGAATGGTGGTGGTGCTGA
TATCTGGATTATTCATGGAAAGGCCGATGTTGGGGCCGTAATTGGGGTTCTGGAAGGAGA
GGAATCCGGACAGGTCGACACAGGGCTTGGCAATGTGATTACAACCTGCGGCAACGAACC
TGTTGAGGAATAATTCCTGGGTGGAGTTTTCCAGGAGCAATGGATGGTATCTTTCGACCT
GCAGGGGGACTACTGGTGATTCCAGGGCTGATCCTGATCCTGATACTGGTCCATGGACGC
TGTTGGAAATGTTGTTATCGGGGTCAACGAAGCTGAGGTCACAGCTGGAGCCTTCTGAGG
CCTTGTCATTGTTCTTGCTGTTGCCTGCTGACGCCGAGGTTTCCAACTGGTCGTTAATGC
TGTTATTAATATTCTCCTGGAGGGGTTTGTGGGTGTTGGGGTCTATCCCTTTCTGCCTCA
GCTTTTTCTTGATGGAGGAATTCCACAGGTTCTTGATTTCGTTGTCGGTTCTTCCAGGGA
GTTGGGCAGCTATTTGAGACCACCTGTTTCCAAGAACTGAGTGCAATTCAATGATCAAAT
CCTCCTCCTCCTGTGAAAATGTCCCTCTTTTCAAATCAGGCCTCAGGTAATTGATCCACC
TCAATCTGCAGC
>RMYB15
CTCCGCCTCCGATGGGTACCTAGACTAGACAATGCAGGATCGCCTTTACCGGCCATTAGG
CTCAGCTGCAGCTACCGATCTGCATCCCATCTGCTCGAGAAACTTGGACAGTCGGATGTG
CTTGGCAGCCCATTGCTCGGCCAGTTTCTGCTCTTTCGCCTCCGCTTCCCTCCTCAAGCC
TGCCAATTGTTCTCTGTACTCAGTTTCGATCCGTTCCAGTGTAGCCTTCTCCTCTTCCCT
TAAAGCTTTCACTTTTGCTTCAATGTCTTCCATTTTCTCCCTCTTACGGTTTGCTTTCTC
TGATTCCAGCTGCAGTTCGACCCTTTTCAATCTCCACGCGGCTTCTTTCTTTTGCGAGGC
AAGTGCACGGTGCCCTTCTTCCAATTCTTTACAGCACTCGACTAGTTCAGAAACGACTGT
GCTCTCCCCAACCATAGGGGCAATCCCGAGTGAATGTATATTGTTCATCATACCGTGTGT
CGCGCTATCAGCTAATCGATCAGGAGGTTGTAGCCACGGGATTGGTGGTGTAGGTGGCAC
AGTCAGGGGAGAAAGGCTTAATGTCACGGATGGGGAAGGCGGCCTGACAGTTGCGGTCGT
GCTGGAACTCGCTAGCCATGGAGGCAGCACGGAGGGCGCCGAGGGGGCAGCTTGTTCTCC
GTGGAGATATCCGCCATTGGAAGCCGGCATTGTGATCCCCGGCACGATATGCTCCCTGAC
TAGCTTCTCGGCAAACGTCTCCAAAATATGATCGTACTTTCCTTCTTCAATCGGCTCAAT
TGTCTTGTTATTCTCCTTCTGCTCGCGCTGCTGCTTTTCTTTGAACACTTCCCACCACTT
CCCGAGCCTTTTGGCGGTTCGACCAGGGACTTCAGCTGCAATTTTTTTCCATTTGTTGCC
GTGCTTGGCCTGCAGACGGATGACAAGTCGCTGCTCCTCTTCAGTTAGTGATCCTTTCTT
GATTCCGGGTTTGAGATAGTTCTTCCACCTCTCTAAACAGGATTTCGCATCCCGGTTGAG
GGGTTGGTTCATGCGCTCCGACACAAGGTGCCAATCTCTCGGTCCATACTGCTTCACATA
CGCACGCAACAAAGTGTCCTCTTCAGGTCGCCAACGCTGCCTTTCCTTCATTTGTCGACG
CGAGCTCTGCCCATTGCCTCGAACTCTTCATAGCTATCTGATAACATAGTTAGAACAACA
AAATTGCAGAACATAAACTACATTGCAAGATCAGTTATTTACAGAATCCATTGAATATTT
ATGACCATTGCAAAACAGGACATATTCCCAAAAGCATTTCAACTTGACGGAACAGGACAC
CATAAACTGATGATTAATTCTTTCATTACAGGACATTCATTCGACTGAATAAAAATACAC
ATAAAAGAAAGTAATGGAAAAGGAGTAGAGTAGAGATCTGGGAAATCTTTAAGAAAAGCT
AAATTCATATCAATTTTTCATAATTCAAGCAAACCCTAATGCTATATCAAAATAGGAATA
TGTATATATAATAAAACAATACGGTATGATTGTCACATCTCTGATGATGATGAAACCTCT
TCATTCTTTTCAATTATTTAATTATCATTCATATAAGCCAATATGTCAACAAAGTTAAGA
TTTGCATTTAATATTCTTCTCAGAAACCAAGGTTACAAATATCCACACAATAAACTAATC
ACCTCAAGGAGACAAGACAGCAGTATATTAGCTTCACATTTGTCTCCATATATGTTTGCA
ATAAATTATGCAAGAAAGAAGGGGGGATGAAACAGGGGCAGCTATCTGGAAACAAGGCAA
GAATGAAAGGGGGGTGTCACTTTATCTTCTTAATAGAAAAACCAAAAAAAAATGAACACA
AAGGCACACCAAACAAAAGCTCAAATTGATTTACCAGAGAGAGATATCCCACAAAGATAC
CATAGTCTTGCTTAAATCCAAAACATTTCACATCATTACGACCTCATTTAGTGCAAATCT
ATACAAAAAACAACAGTGTATGTAAGGAAAACAGGCTTACAGTAATGTTCAAGAAATCCT
GGTCTCTCCACAGCCCATTTCTTGATTTTCCAACAAGAGCTATCTTCACAAATTAAAAAA
AAAAAAAAAAAAAAAA
>RMYB16
TGACTTCAGTACGTCGAGACACCTCGGCGCGCCCATTGGCTTGGTCGGGCCGGCGGCCGG

CTCGACACACTGATCCTGGAAAGAAGGAGAAGCCGAACAAGATCGAAGCTTGGACTGGCG
GGCCAACCTGGCTTCGGCTTCGAGACGAGCGCTCTCCCACTGGGCCATATGGCTGAGGTT
AGCGGCATTCTTGGACTGTCCATCGCAGGACAACAGGGCATCATTTTTGGGCTTGTGGGT
CACCGGGTCGATGCCCATTTTGGCCAATCGTTTCTTAAGGTGAGTATTCCAGTAATTCTT
GATCTCGTTGTCTGTTCGTTTGGGCAAATGCGTGGCTATGGCCGACCACCTGTTTCCCAA
AAGGGCGTGAAGCTGAATGATTGTCTGCTCTTCTTGTAAGCTGAATTTGCCTCTTTTAAT
ATCAGGTCTCAGATAGTTCGTCCATCTTAATCTGCAGCTCTTCCCACATCTCTGCAGCCC
TGGGAAAAAACCCCAAACAGGAGTTTCAGACATGAAACTACACATAAAGAGAGAAAAGAA
AATCTTACCAGCTTTTGCGGGCAAAGCACGCCAGCTACCATGGCCGTGCTCCTCAATATA
TGCCAAGAGTTTGCTGTCTTCTTCGGGCGTCCATGGCCCTTTCTTCAATCCTATTTTATC
ACAGCATGGAGACCTCCCCATTGGTGCAGAAACTGAGAATCAGGCTTTAGGAGGTAGTTT
GGTTCTGCACAAGAGTATATAATTAATTTCTGTGTGAGAGAAGAAGGGAAATTAGGGTTT
GCCAATAGGTGAAGTGAAGTTAGGGAGTGTGATGATGTGGTAAATAAGGGGCGGAATGGC
CAACATGCCAAGCTAAGGGAGTGGGGAAGAAAGGGATGGAAAATTGGAAATGGAGGTAGT
AAATTAAATGCACTTTCCTTCTTTATTTTCTTACATCTATTTTC
>RMYB17
GAATCCTGTTTAATTATTCAAATTGTTGAAAAAAATTTGAAATTTCAAACATAAATAATT
AAGAGATGGAATTTAAATCATTAATTTGGACCTGGAAAGAAGGAGAAGCCGAACAAGATC
GAAGCTTGGACTGGCGGGCCAACCTGGCTTCGGCTTCGAGACGAGCGCTCTCCCACTGGG
CCATATGGCTGAGGTTAGCGGCATTCTTGGACTGTCCATCGCAGGACAACAGGGCATCAT
TTTTGGGCTTGTGGGTCACCGGGTCGATGCCCATTTTGGCCAATCGTTTCTTAAGGTGAG
TATTCCAGTAATTCTTGATCTCGTTGTCTGTTCGTTTGGGCAAATGCGTGGCTATGGCCG
ACCACCTGTTTCCCAAAAGGGCGTGAAGCTGAATGATTGTCTGCTCTTCTTGTAAGCTGA
ATTTGCCTCTTTTAATATCAGGTCTCAGATAGTTCGTCCATCTTAATCTGCAGCTCTTCC
CACATCTCTGCAGCCCAGCTTTTGCGGGCAAAGCACGCCAGCTACCATGGCCGTGCTCCT
CAATATATGCCAAGAGTTTGCTGTCTTCTTCGGGCGTCCATGGCCCTTTCTTCAATCCTA
TTTTATCACAGCATGGAGACCTCCCCATTGGTGCAGAAACTGAGAATCAGGCTTTAGGAG
GTAGTTTGGTTCTGCACAAGAGTATATAATTAATTTCTGTGTGAGAGAAGAAGGGAAATT
AGGGTTTGCCAAATGGTGAAGTGAAATTAGGGAGTGTGATGATGTGGTAAATAAGGGGAG
GAATGGCCAACAGGCCAAGCTAAGGGAGTGGGGAAGAAAGGGATGGAAAATTGGAAATGG
AGGT
>RMYB18
CAGAAGACGGCATACGAGATCACTGTGTGACTGGAGTTCAGACGTTGCTCTTCCGATCTC
CCCTTATCTTTTTTGACAGATTATTCTGCAGATTTCCATCAAATACAACAAATACAAAGC
GCCATAAGATACAACAAATACCCAGAGCTCCTGCCATAATTACATCTTCTAAGAAGAAAT
TATCTGAGCGCCAAAAAACAGATTGTTTCTGAAACTGTCAAACTGCCCCTATCTTTTGCT
GCTAAGCTGAACACAGGCAAAATAACAAAAATTAAAAAGGAAAACAAGAATAAAAAACCT
ATAATACATCGTGCTGGAGGGCTTCGGTTGAAAGCCCTCTCACCGTTCACGCTACCTGCC
TACATCCCCATTACTTTTCCATGAGCCATCCTCCTCTCTCTGCTCATTACTTGCTGGTAC
ACTAATATTTTGTGCTTTAGCATAATGCATCTTGATGGCAGCCACAGGGTCCGAAACACC
AGAGCATTGCCCTCCAACCACCATCCCACCTCTGCCAATGCCAATCCCAATCATCCTTGG
TTCGTGTTGTATTTCTTGCACTGGGTCAAACCCATTTCTCCTTTGCATCTCAGAGAATAT
GTCCTGCGGTTGCTGCTTCATCTCCTTTGTCAAAAACTGCAAATCTTGGTTTTTTAATAC
CTCATAATCTGCATTGCTGCTGCTCAAATCTATGCTGCTTTGGAAGGCAGTGACACCATT
CAAGGGAGATTCACTACTCCTAACCCCTTGTAAAGACGGTAGCTCCAGTTTACCTGAGGG
TTTGGAATGGCTGCTGAATGCAGCAGGATATTTGGCCAATACGAGAGTGGAATCAGGCAT
CGATGGGAAGCCCATCAGTGTGTTGTTCCCATCCGGAAAGCCGTAACTCCTAACAGAAAT
ATTCTCAGATCCAATAAATTTATTACCACACACAGAAGAATCGAAAGTGGATTTGTGATC
AGTGCTGCATCGGAGGCTCAGAGACTGGCAACTTGCTTTATGATGCTCCACTTTATCACT
GCAGGCTTCTTGTGCACAGGAGTTTGGTTTTTGCTGGGAGGATATCAAAACTTTTCCAAA
CAATTTCACATCACCATTCCTAGCAGGCTTATCCAAATTAGACGGAGGTCTAGAGTTATC

ATAGCTGCTCTTTCCGTGAGATTGAAATGGCGTCTGGAATGATGCTGCTTCACTCAGATG
TCTGGTACACTTTTGAAGGGAGAAATCAGGATGTTGATTTGAATGCAAGCTTAGATCCCT
CTCGGGGAGATGTTGAACTGCATGCCTCTTGCAATTCACATCGCCATTGATCTCGTTCAC
TGACGGCCCGGAAACAGGGTAACCCCTGAGAATCTGGGGAGAATCTACAGCTTCTGACAG
CAAGTAACCTGAAAGATTCTGCTGATGGTCCCTTGCCCCAACTGCCTCCAGAGAATGTTT
TTCACTGATCCTGCTCGCACCAACATATGCTAGAGGATTACAATTTGAGTGTATTTGATA
CTTAATGGGAACAGAGAACATCATAGATGATGACTCTATTGACACCATGTGACCATTCTC
GGGGGATGAGATGATGCAAGATTTATCAGCATGAAAGTTATCGAGGTTTTGGCCACCAGA
TTCAACTTGCATAGAAGAAAATGCACCAACAGGTGAAACATTCCCAGCCAATTGCAGATC
AGATTTCACCTCACTGATTGCACCGCTTATAGCAGTGATTTCACTAGAATTGAGATCTCT
ATCTTCCAGTTTCTTAGCATCAAAATTATCGTCAGATAACAGGGGTCCTTTTCCTAAATT
TTCTTCACAAAATGACCCGGCAGAATCAGGTGGAACCTCCTCCTTATCTGTCTCCTTTGT
TCCAACAGCTACAACAGAGCCACTACCCTCTTCTACCAGCAGAGATTCTGTGCGGGAGGA
TACAACCACAGTTGGACCTTCCAGCTCAGATCCACATGCACCTTCACTCTTTCCTTTGCC
AGCTGCACCAGCACCAGAGTCTGCAGCTTGTTTCTTGTCCAACAGAGTATCACCAACTGA
CGAAGTTTCCAAAACAGGCTCTGCAGCCAGAGAGTGAACATCAGGCTTCAAATTGTGTGT
TGCTACTGCATCAGGCTCATGCTTTCCCTTTGTATTGGGAGGCGGAAGATCCTCTTCCAT
CTTGCATTCAGAGTCACCATTGCAAACAGCGGAGCCAGCCTGCACAACACAACCATCTTC
TACATCACTACCACCTCCATTAGCATTGTTAAAAACTGCATTGCCAGCTCCATGATGAAT
CTGATCCAGCCCAAGACACTTTCGAGCTTTGCTAAAGAATATCTTGCACTGCTCCTTGGA
TTTTGTTCTAACTATCTGTGCAATCATATCAAAATCCTTGCCATGTGATGACACAGCCTG
GACAAAAATGGATTTCTCCTCATCAGTCCAATCAGCTGAATCCATCTCCTCAAAGCTCTC
ATCCGAGCACTCATCGTCGACATTCTGAGTACAATCAGGGGTCAAAGGCCTCTTAGAGCC
AGAATTGACTCTTGGACACTTCCAATCCTGATACCCATCGGCATGGTCAAAAGAACTCGT
GATGCAAGAATTCATGGCCTCAGATGACAAGGAACCACATATTCCAGCCAACACATCAGC
AGCTACAGTTTCATTGCTGTAAATTTCCTGACTATTTGATCTCTGTAGCAAACCATTGTT
GCCCCTTGTGGCTTCCCCAAGTACATTGAGAGATGCTGCATTCACCTCTCGATTCCATCT
TTTCCCAGTGGCAACCAAGTATGTGGTAGCTTGTGACTTTCTTTGCTCCGAAAACTCTGG
CTTCTTTCTGGCTCTTGCAAAGCATTCAGATTTCTGGTTCTTGTAGTAAAACTCAATGCA
GTCAGCAATTGTTTTGTGTTCCAGGAAAGATGCAATCTTACTGAAATCTTTCCCAAAAAC
AGCAAGCTTATCAATGAAAATTTCCTTTTCCTCAGCAGTCCATGGGTTGATAATTGACCT
TTCTTTTTCAACAGCACAAGGATCTTCCACTAGACCATTGTTTGAGATAAATCTTGACAT
CTTTACATCCTTGTCCAGTATTAAGGCTGGCATCTTCAGAGTATTCCTGCAGGGTTTGAA
TGATGACTCAGAAAGTAATCCATGAATGAACTTAATTACTTCTTCGGCAGGCACTGTCCG
GTGGTTTGAAGCAAAATAAGGAATTCGAGAACGACTGGAGGAACGATTCTTCTTATGCCC
ACAATGAGTCATATCTAACATCTTGTTAGACTTTCCTCGAAGCTTCCTTACAGATATTAT
GCGACCCTCCCTCCAGAAATGCTGAAATACTCGAAACTTGAGAGTTATAACTTTCTCCTT
AAATTTCAGGGACCGCTTCCTCATTAAAAACTTCTCCTTAACTGACGAACTCCTTCTGAA
AGGGGGCATAATGGATGCAATTGATAAATCGAAGAAATAGTTTCTGGAAGGTAATAACTT
ATTCAACACCTCTAAAGCTCTACCAGCAGATTCTTTATTGGAAGCCACAATTATGTCATA
GTTAGAGTCCCCACCACAATGAACATTGCCAACATTACCCTTTTGACAACTGTCTGAAAT
AGGTTTAGCAGCATCGAGCTGACAAGAATTAATCTCATTGCTAAGACCATTCTCTAAACA
CAACATATTCAAGTTGCTGGAATTTTTCAAGTCCAAATTGACAAAACCTTCACATTCAGC
TGTCCCAGAAGCACAGACATCTTCCCCAGAGGAGGGCTCAACTATTTGATACGTGGCACA
GCCTGGACTATTTATCTCCTCAGATTTCAATGCCTCAGATTCTTCTACAGAAGCAGGCAT
ATTCTCAACATTCATGCTTCCAGTCGAAGCAACCTGAAAAGTAGGATCAACAGTAGAAGC
AGAAGCAGCAACCAGCTCCATACAAGGTTTCAAACGGCACTCTCCTTGTAGCAAGCTTGA
AGTATCTGGACAAGGACAGCAACTTCCAGGTTCAGCTATCAGTGACTTGAGTTCGGTATC
AAGTGAATCTATCTTAGATTCAGTCACCTCAAGAGCTTTTAATACGTCAACCTTCCATAC
CAAAAGCTTCTTCATTGAGGTAGTTTGTACATAATCTGTTTCCGCAGAACTCGGATCCTC
TGATTGCAGCAATTCATTTAGGAAATTACTCAACTTGGCAATAGATGCAAGGTCAATGCT

TTCTAAATTAAAAGTTGGTCCCTCGCAGTGTTTCTGGGACATAATACAGGGCAAACCAGA
TAAATTATTATTCTCTTGATCAACACTTGCTGGCCTCATGGATTCTTTCTCCTCGGTACC
TGGTGAAGAACTGAAAGCAACAGATGATGGTGTGGCAGGAGAAGCACAATCTGACATACT
TGCAATTCTAGGGCTCTTATCAGACAGGTTAACAGAAGGTGAATGGACATTTTCAGTTAT
ACAGACACTAAGCACCCGTCCATCTTTTACAACACCATCTTCAGGGCCTTCAACTTTCTT
TTTCTCATACTTCGCAAGACCCTCACCCCAGCCCAGACGTGGTTTCTTCCTGCAACAGGT
CTCATCAGATGAAGCAGGAACAGAGGACCTGACATTAGAAGTACAAGGAGACTGAACAGG
CATTGCACTCTTCTGATGTGCCTCGGCCACTACCTCAATAGAGTCCACTCCTGTACTCTT
GGTGCCACTCAAATGGCTAAAACAAGGACCCCTTGAGGGCAAACTTCCTGATCGAGCCCA
TTTCAGGGGCTTCCAATCTACTGAATCTAGGCCATTTTCTTTCTCAGATTTCTGAGCCGA
GGTAGCCCCTCCATCGGCAGTACCATCATTTTTCTCCTGCTCTTTAGTATGTGATAAAGA
TTGGTCTGATGTGGAATCAGGAAGAGGATTTGGAGAGCCATTGACCTTACTGTTGTTATG
GCATGTTTCAGAATTCTCTACAGACTTCTGGTTATTAACCTCAGTAGTGGTGAATCTCCC
AGGACCACTGGAAGTTGCAGCTGGTTCCCATGAAGTAGATCTCCAGTCTTTCTGGCTAAA
GGAACCTCTATTTTCCCTATTGGTCCTAAAATACCTTGCATCATTGCGAGACCCTAAAGG
CTGAAAGTTGTCATCTTCTAGGTGCCTGTCACCATATCTCGACCCAAAAGGCATAAATCC
ATGACCAGCATCATCTGGGTAGCCATGCCATCCACCCTGCTTACCATGACCTTCAGCAAA
AAATTGAAAATCCAAAAAGAGAGCCGAAAAACAATTAACACAAGAAACTACATGTAAAAA
AGGGCCATATCACATTAATATAATCCTCAAATAAATTATAATTTTACATTTACACACAAA
AAGGGAAAGGCCGGAAACAGATCTATACAACACCATCCACAAGAAAACCTCTGGTTTAAA
TGGTAAACATCAGATCTGCACCACATATGCCCACCAAACCCTCCACGGGATCACCTAATA
AAGAACTGAGCCCATAATCTTCCATGACTAATTTAAAGAATTCATATAAACAAACAGGTA
CCGAAAAAAGGCAGTCGACTTACAAATGACTGTGACCACAAGAAAACTCGGAACCATCAT
CATCCTAATAATCCCACCATATCCAACACTTTGATTAAACAACAATAATAATCTGAACTA
GAAGAAGCACCTAATATTTCCACAAAAGACTCCAACTGGTAAAACTCTGAAAAAATAAAA
ATATATATAGGATTCTAAAACGGTACTCACCGGAAGGGATTGGGCGAGATGAGCGAAAAT
CAGAATACCAACGCTGCTGCTGCTGATGGTTGTGGTGGTGGTGATACGGCGGCGGGTGTG
GCGGAGGCGGCGGTGGCGCGTGGGTGTGATGGTGTT
>RMYB19
GGAACTTTCCTTCCTAATCATTAAATCCAACCATAGGGATTCAAGCATTACCAAATTCAA
GTAATTTGGTGACCCATAGATTCTCATCAGAGAGATTGAAAAAGTCAATTCACATCGAAC
AAAGATTCTGACCCCTCTGTTTCATTACTACTACTAACATTTCTATTTCTATTTCCATCT
CCATTTCCGTTTTGCTTCTTCTCTTCTCCAATTTTCCCACTGCAGCCCTTTACCCCTGTG
GAAATTCCTGGTGCACCCAATGCCCCCTGCTTCAATACCTTCTTGCTCAAATGATAGTTC
CAGTAGTTCTTGATCTCATTGTCTGATCGACCAGGTAGTCTTGCTGCTATCATTGACCAC
CTGCTTCCCAGGAGTTTGTGCAGCCTGATGATCAAATCCTCTTCTTGATCAGAAATATTG
CCCCTCTTGATGTTTGGCCTCAAATAATTCATCCATCTCAGCCTACAGCTCTTTGCGCAA
CGGTTCAGCCCTGCTTTGGCAGCAATGGTTGGCCATTGTTTCGCGCCATGGGTTCCAATG
GCTTGCACCAGTTTCCGGTCCTCGTTAGCTGTCCAAGCACCCCTGTTGAGTTCCTTCCTG
CCTGCCTGCTTCTCTTTGATTTTATGGGCCATTAAGGCCAACTATTTTCGCTATATCGCT
GCTGTTAATTTCTGACAATTCAGAACTGAGGGAAGCTTTTGTGGTCAAATGCCTCTGGCT
ATTTTAGACATATGGTTGGGGATTTTTGTGGCTCAAATGCCCCAA
>RMYB20
GTTGTTTAGGATTAGGGTTTCCATTCTGTCTCAATAGTTTTTATTATTCCCAAACATGAC
ATAAAGTCAATGACCATATTTGCCACAAAAATATAACCCAAAAATTTGGCAACACATCAT
ATATAGTGAATGACATACATTCAATAATATAATAATAATAATTCCAGTCTTTAACATTTA
CACTCCATGCATGCAATCAATAAAAATAAAATTAAAGAAAAAAAAAAAGAAAAAAACAGAAG
CACAATTAATTAATTACTATACCCCCTTAACAATCCTTATTACTTTTTTTTCGGATTTTA
ATAATAAAGCTCTCTATTTCACACCCTACCCCTCCCTATTATATTTATTTTATTGTGCCA
AAATAATAAATAATATAAAATTAACCTTCGGTAAAAACAAAAAACTAACCACTAGCTTCT
GTAACTTTTTCACTCTTTTTTGAAATTTTTTTCACCGCCACCGCCGTTGTCGCCGGACCC

ATCTCCGTTATATCCGCCCACTTGCTTCCTGCAGAACAATATCACGATTCCTGCGCGGCA
TTCGCCACCGATGAGTGCTGCTTGTTCGCGATAATTTCGAAACCCAATCCCTCGTCGTCG
TCGTCATCGTCATCCTCGTCGTTGATGTTGTGATCGTCGCTTTCGTTGTCGCGATTGTCC
TGATCCTGCATTGCGGAATCCGGGAGTTGTTGTCTGTGTAGATTGGGAATTGGCCACTGC
TGCTCCGGCTGCGCCATTATTGGCACCATGGGGTTGTTATTGTTGATCTCCTTGGCTCTT
TCTTTGTATATGGCTTCCAGCTGGTGGAAGTAAGGGCACGTCTTAGCGTCCTCCGGCCGC
TTCTTGTTGCTTTCCTTCACCTTCTTGAAGTACTTGTTGATATTCTCCCATTTCTCCTTG
CAACGCTTCGAACTTCTGTTGTATCCAAGCTTCGCCATAGCGGACGAAATTTCCTCCCAG
AGAGGGCCTTTTGGCCCGTTTCTTGGTATTTTAAATCCATGCTTGTTCGTAGATTAATT
AGCGCTTGAACTTCCGCTTTTGGCCACCGTGATGAACTCGCGGCCGGGGAATATCCGGCC
ACTTCTCCGCCGTTGTTGTCCGTTTTTGAATTGTTGAATACTTTCGCCGGTGGTGGCACT
GTAATTGGAGCTGGCACCGGTTGTGTCGTTGGAGCTGGCGAGGCTGGTGGAGGCTCTGGT
TGCTGCGGTGGCGGAGGAGGAGGAGGAGGCCGCTCCTCCGCCGCCGCCACCGGCGGGATC
TGCAAATTGTGTTGATCAGTTACTTTTTGTAAAAATGCAATCACGGCGGCGTCTTTAGTG
GCGGCGATGGATCTCTCCTGGACTAATAGATCATGTTCGCGATTAACTCTGGCGATTTCC
TGAACGCGCCAAGCTTCTTCCCTCGCCATTCTTTCGCGCTCGCGTTTCTCCAGTGTATCC
AGGAATTTCTTCTGCAATTCCTCCTGTTTCTGAATAACGTCCCGCACCAGCCTGTGGAAG
CAATCCTTCCATTTCCTCTTCCTTCCACGCCGTCTTTGGATATCCTCGTCGGACGAAGTC
GACGACGACGTCGAATCCGACATCAGCCCCGGGCGCGATGCCTGAAAGTTGAGTCCTTGC
GTAGGAACCGAGCTGTTCCTCAGATGCTGCATTAGCGGCGGCGGCGGCGGCTGCGCGATT
AAAGGAGAAGAATCGATAGGGATTGAGGCAGCTGCCTGGGGAGGGACCACGCTTAAAGGC
GTCGGACTAATTGATGAAACAGTAACATGGGCTGGCATTGTCGGAAGGGAAGCTGTGGCG
GCGGGGCGCGGCGGGGTGAAGGGATTAGGCGGCGGAGCGTTCTCCAAGGCTTCCAGTTGA
TCGAAGAATCGGTAGGTTTTGCCGTCGGATTTTGCGGCGCGGCCGTCTTTGGTTCGCTTG
TGGTATTTGTACACGTTCTCGAATTTCTCCCGGCATTTCTTGGCGCTTCGATGGAATCCG
AGGTCGGCCATTTTCCTGTAATTACAGTTGCAGAAGTGATGTCGGATTCATGATTTAAAA
AAACTCGAATTCAGACAAACACATAAAATAAAAGGAAGGAATTAACTGCACTGATTAAG
>RMYB21
TAGACCGTTTCTGGTTTCTCCTAGCGGTACTCAAGCCAGTGAAGTATTTCTGAGCATGGC
TAGCCACTTGCGTATTTGTTTTAGTTGGCACACAATAGCTTGAAATGCTCTTCCAGTCGC
CTTTTCCAAACTTCTCCAGGCCGCGTAAAAAAGATCTATGCTCCTCTTCTGTCCAACGAA
ATCTCTTCTGCTCATGAATTCCCAGTGGTTTCTCTTTCTTAGCTTTATTCTTACTAAAAT
GATGAGGCAATGGTACCTTTCGAGACTCGACTATTAAGGCTTCATAGTGAAGTTTTAACT
CTTCAATAGATTTTCCAGGAACTGCCAACACAACCTTATCCCATAGATCAGGATCACCAC
GGAAGAATGCCAAAGCATTGTCAAATGCCTTATCCTCTTCCTCAGTCCAACTCGAACCAC
CATGTTCTTCATCCTTCCAATGTATCCGCTGTTTCCCACCAATTCTCAATGGTATCTCTT
TCTTTTCGCAAACCATAATATTGCTTGGATAATGAGGCAAAGGCACCTTACCTGACCCAA
TTGCCTCCACATCATATCTTAAGGCTTTATAGTGATGTTTTACATCTACAATTGTCTTTC
CAGGAACTGCAACTGGGAACTGCTTCCATGGATCAGCATCATTGTGGCATAGTGCCACAG
CATTCTCAAATGCCTTATCCCTTCCCCAAGTCCGAGATGAACCACTGTAAGCGTTCTTAA
ATGATGCTTTCACCTCCAATGCCCTGGAGCAGCTGCTGGATGACCCACTGGTGTCCATTC
TTCAGGTAACTGCCGAAGAGAGATGTAGCCATCCAAGTACAAAGGGAGCACGAGACACCT
AGTCTGAAGCCACTGCAGGTTTCTGAATTCAGACATGTTGCC
>RMYB22
CTTCAGCTCATTTACAGCACTTGAGAAAGCCATATCAACCAAATTAGCATCTCTGCATTC
AACCTTTTTACTGTTCATGTTTTCTTCAGGTAAATTATGTCCTTCCTGCTGATTGTCTCC
CTGCACATGGCCACCAGAAACCTTCACTGAAGCCTTGTTATAAGCTTTGCTCAATCCATC
AGGCAGGCCACAATCAAACCCCAGTTCAACTTGTGGTGAATTGCTATTGGAGACAAACAT
GGTTCTAACATCCAGCTCAGATCCATCTGCACCTATTTCCTCTTTGCTGCCTGAATTTAA
GTCTAAAGCAGATTTATCATCCACTAGAGAGTTGCTCACTGACGAGTTTCTTAAAACAGC
ACCAGCAGCCATAGAATCAAGAGGGCATGCTCTGCTACCCTCACCATAGTCGCTAGCATC

AGGCTTCAATTTGTGCGATTCTGCAGTATCAGATGCCTGATCCATTTTCATCTCATGAAG
GGTAAGATTCTCTTCAATCTTATTTGCGGAATCATCACTGCAAATAACAGAGCCAATCTG
CACAGCACAACCATCTTCAATATCACTGCCACCTGCATTAACATGACCAGTGCCAGGATG
AATTCGATCCAACCCAAGACACTTGCGGGCCTTGCTAAAGAATATACTGCATTGCTCCTT
AGTTTTTGTTCCAACACATTGCGAGATCCTTGCAAAATCTTTGCCATAAGATGACATGCT
CTTAATGAAGATGGACTTCTCCTCATCAGTCCAATCAACAGTCTCTATCTCCCCACAGCT
CTCGTCTGAGCATTCGTCATCAACATTCTGTGTAACATCAGGGGTCAAAGGCCACCTAAT
GCTAGAACTGGCTCTAGGATCAAAAGAGCTTGTGATACAAGAACTCATAGCTTCTGATGA
CAGCGAACCACAAATTCCAGCCAACACATCAGCTGCAGCAGTTTCATTACTGTCCAAATC
CAGACTGTTTGATCTCTGCAATGGACCATCATCGCCTCTGGGAATTTTATAGGAACTAGC
TGCACCAAAAAGGATCCTAGATGTACATTTTGGCTGCTGCTGAGTTCCTAATCCATCATT
GACAGCAGCCACAATTACCGAAGCATCACCAAGCATATCCAGATTCACACTGTTTGCCTC
ACGATTCAACCTTTTCCTACTAGCCACCAAGTATGTTGAAGCTTTCGACTTGCTTTGTTT
AGCAAACTCTGGCTTCTTTCTAGCTCTTTCAAAGCATTCAGATTTGTGATTCTTGTAGTA
AAACTCAATGCAATCAGCTATTGTTTTATGTTCCAGAAAAGCTGCTATCTTACTGAAACT
TTTCCCAAAGATAGCAAGTTTATCAATGAAGATTTCCCTCTCTTCAGCCATCCAAGGGTT
TATGATGGATCTTTCTTTCTCTGAAGCACAAGGATCGTCCACCAAACCATTATTTGAGAT
AAACCTTGACATCCTTTTCTCCTTCAAAATAAAGCCTGGCATCCTCAAAAAGTTCCTACA
GGGTTTGATTGCCGATATTGAAAGCAACCCATTAATGAACTCCACTATTTCTTCTGCAGG
CACAATCTGATGACTTCCAGCAAAATAAGAAATCCGAGAGCGAATAAAGGAGCATTTCCT
TTTATACCCAGTCCGACTTAGATCAAACATCTTGTGAGACTTCCCCCCAAGTTTCCTTGC
AGAAACTACACGACCTTCCTTCCAGAAATGCTGAAATACCCTAAACTTGAGTGCAATGAT
CTTCTCCTTAAATTTGACGGACAGCATTTTTGCTAAAAACCTGTCCTTAATTTTCAAGAA
ATCCCTCTGAGAAGAGGAAACACTGGTCACAGTTGAAATATCAAATATACACTTTTTATC
AGGCAATAACTTATTCAACTCCTCTACAGCCCTATTCGCTGACTCCTTATTGGAAGCAAT
TATAGAGTCATAAATATGGTCACATTGGATATCGCCCACACCATCAAGATGCTGCCAGTT
ACTCATTGTAGAATTAGGAATATCGACAGGGCACACATTATCATCATCACACAAACCATT
CTTTAGCAAAGTCTCATTCAAGTAACTGGCATTGTCCAACTCCAACTTAACAAAACCCTC
AGCAAATTCTGTAGTCTTAGAAGTACAAGTGTCTTCCCCATGTGACAGTGCCTCAACTAG
TTTAGAAGTTGCGGTACCCGGACTATCAATCTCCTCATCTTTCTGCTCCAGATTTTGGTC
TTTCAGCGCACCAGGTGTGTCCCCACTGTTCATGTTTCCAGACAAAGCAATGTGCAATAG
AGCAGGTACAACAGTAATACCAGAAGCAGCAGCATGGTCCTCAGAAGGATGACATCCATC
AGATTCAGCTGTCAGAGACTTGAGTTCAGTTTCAAGAGAATCTATCTCAGATTCAGTCAC
TTCAAGTGCTTTTAATATGTCAAGTTTACATAGCAACAACTTGTTCATCGAGGTGGTCCG
TATATAACCAGTTTTCGCAGAACTTGGATCATTAGAGTTTTGGAATTCATTTATCAACGA
GCTCAAATTAGCAACAGATGTGAGCTGCAAGTTTTCTAAAATAAAAGCTGGTCCCCCATG
GTAAATCTGGGACGTAACATCAGGCGAACAGCTCATATTTGCTGTATTCTGATCTACATT
TGCTGCCTTTATGGATTCTTTATCATCAATACTTGTAATTCCTGGGGAAGAACTACAACC
AACAGAAGCTGGAGTAGCGGGTGAGACACATTCCATAAGATTTGCAAATCTAGGGCTCTT
ATCCGCTAGGCTGACAGAGGGTGACTGCATCGCTTCGGTACTAGTAACACTAACAACCAG
TCTATCCTTTGCTGCACCATCTTCACGGCCATCAACCTTCTTCATTTCATACTTCGCAAG
GCCCTCACCCCAGCCAAGGCGCGGCTTCTTCCTGGAACTGGTCTCATCAGATGGATCAGG
GGCATTGGGCATGGCACAAGCAGCACCTGGAGACTGAACGCGCATCACATCCTTCTGCTG
CACATCAGCCACAATCTCAGTTGAGTCAACTCCCAGACTTTTGGAGCTACTTGAATGACT
GAAACCAGAGCCTCTTGACACCAAACTCCCAGACCGAGACCATTTCAGGGACTTCCAATC
AATCGAGCCTAGGCCATTTTCTTTGTCAGAGTTCTGAGTCAAGCTCGTCAATGTGTCAGC
AGCTCCGACCTTTTCATGCTTTTCTTTCGGGCACGGTTGGGATGGAGATGATAAGGATTT
AGGAAGAGGAGAAGCAGAGCTCACAGTGTTGTTCTCATGCCATGTTTGAGTATTCCCTAC
GGCCTTCTGATTACTCACCTTAGAGCACGTCCCGGTATTCTCTATGACTTGATTATTCAC
CTCGCCCCTTGACTCCACACTACCAGACCGAGAACAATTCAGGGGCTTCAAATCAATCGA
CCCTAGGCCATCTTCTTTGTCAGAGTTCTGACCCAAGGCCGTCAATGTATCTGCAGCTGC

AACCTTTTCATGCTTCTCTTTCAGGCGAGGCTGGGACGGAGATGATAAAGATTCGGAAGG
ATGAGCAGAACCCACACTGTTGTTCTCATGCAATGTTTGAGTAGTCCCCATAGCCTTGTG
ATTATTCACCTTAGAGTATGTTTGGGTATTCTCCACC
>RMYB23
GATCTCCCCTGGCAAAACTAAATAAGTTCATCCAAACTGACCTTATAATAAATGAAAACT
GAAAACTCATCGTTTCAAGCAAACTCAAACAATCAATAGCTACTAGTAACTAAGAGAAAG
GACCGAACCGATATCCAAAGACGAAAGAGTAGTATCATATATTTTCTTGGGCGTGTAAGT
ATTTCCTCGGATACCTTCCCGTTATATATGTATGTCATTAGTAGCTTCAGTGTCACTTGG
AGTGCTCTGCTTTCATGTATCGTGCAAAATGCTCATGTCACATGTAGATTACATCCATTT
CTTAAGAAAGCTCAAGTATGGGCTTCCAAAATTGGCAAAAATCTACTCACCCCAAGGTAG
TCACCGAAGACAGGTTATTAGCAACAACCTGATAACCGTCTCCACTTTCACGATCCTCTT
CGTCATCCATGCCCTCTCCTTGGTTTTGATGATCATTGTCGCCGTATTCCCCAATTGTCT
GTTCAGGTTGTTGCTGGTGTTGCTCTTGGCCCATCATCTGCATCAAGATTTGTTCGGGCT
TCAAGCTGCTTGTGCCATTCTCTGAGTTGTGGTCAGATTTCTTGGCCCTCTTAGCATAGA
GAGATTCGAGCATATTAAAATATGGGCATGTTTTGGAATCATCTGGCCTCTTCCTGTTGC
TGTCTTTTACTCGCTTGTAGTATTTATTTATGTTCTCCCATTTCTCTTTGCACCTCTTGG
CGCTTCTGTCGTAGCCAATCTTCTTCATACGAGCTGAGATCTCCTCCCACAACGGCCCTT
TCGGCCCACTGTCCTGGTATTGCATATCAAGATCTGTTTTAAGCATAATCAGAGCTTCAA
CTTCAGCTTTAGGCCATCGTGAAGAACTGTTCTGGACAACATTCTCGCCGGCACTATTAT
CCTGCTTCTCGTTGTGTGCAGAAGCTTCACCAATGCCATTGTCCTGCACAAAAGCACGTT
TCTCTGCAACACTCTCTTGATTCTCGGAACGCTTGTCAAACAACGAAGCAAGAACTTCAG
GCATCTGCAGCGGAAGCGCCTGCTGTGTGATTTTTTGCAGGAAAGCAAGCACTGCCGCGT
CCTTTTCTGCTGCTATTGCCCGCTCTTGGGCCAAATACTCTTGCTCCCTCTTAATCCTAG
CCATTTCCTGAGCCTTCCACGCCTCTTCCCGAGCCATCCTTTCCTTCTCGCACTTTTCCA
AAGCTTCCAGGAACTTGTTCTGCAAATCCTCCTGCTTCACCAACACATCCTTCATCAACC
TCTCCAGATAATCCACAAGCTTCCTCTTTTTCTTAATGCTCCCCTCCGAATCCCGTCCAG
AGGAGGAGGCAGTCGATGTGGATGCAGATGTGAACTCAGCAATTGGATAGTTTGAACAAG
GAATGGGAAAATCCTGACTACAACCACCCTGCTTCGCCATGGCTGCTTCTGCTGCTGCAG
GGTCCGATGCAGGCCTGGTGTAGGAGGGAAGTGGATTCGAGGGGGTCGATGGGACCGAGA
ATTGGGCATCCAAAAGCTCTAACTGCTCAAAGAACCTATAAGCTTTCCCATTCTGCCGAC
TGGATCTCCCATCTTTGGTTCTCTTGTGATATTTATAGATGTTTTCGAATTTCTCTTTGC
ATTTCTTGGCACTTCGATTGTAACCAAGCTCGCCTAATTTCCTGGAGACTTTATCCCAA
>RMYB24
ATTGCTATTCAGTGATTCAATCATTTCAAATTTTCAATTACCGAATAGTAAAAGCGTTAT
TTACTTACTCATTAAAGTCGCTAATCGCAATCCGCTTGCCGCTTAAAATTCCATCCCCGC
CGTTCATCTCCCGTGAAAAGTAATTTCTCACTTCCGATCTGATCATCTCTTGAATAATCT
GCAGTAGCTTCGGATCAAACGCCGGACCCGGCGGCGGAAACGCGCCGTTTCCGCCCCGCC
GCGGTGGAGTATCCGACGACGGCGAATCCTTTTCGCTAATCTCCTTCGCCGACGACGAAA
CGCTCCCTGGCAAACTTAGAGTCAACAGAGTCAATTCAATATTGGAATTATCCTCATTCT
CAGCCGCCCCATACTGCGGCGATTCCACCCCCGGCGAGCTCCTCGCCACCGGAAAATAGA
AATTTACGTTGTCTTCGCTGGAATCATTGGTTTCAGAATTTGATAGATTCATGCTCTCTG
GACTTACTCTCAACGCCGCAGACACATCTCCATTCTCCGCTCGCCTCCAAACCTTCGCCG
ACCGCTCATCGCCACCGGAGGCGGCGGCGGCGGCAGCCTTCCGCTTCAGCTTTGAGTTCC
AGTGATTTTTGACAGCATTATCCGTGCGTCCATTGAGCAGCCTCGCAATCTTCGCCCACT
TGTTCCCAAAATTCCTATGCGCCTGCAGGATTCTCTCATCCTCCTCCGCCGTAAACGGAC
GGTGCTCCACCTCCGGCGATAGCTGATTACACCACCGCAGCCGGCACGACTTCCCCGACC
TCCCTGGAATCGACCTGCTCACCAGCGTCCAATTCCGTCCGCCGTGCCGCTCCACGAGCT
TTCGCAGCAGCTCGTCCTCCTCCTGACTCCACGGCCCCTTAATACGGTCCAAACCTCCGC
TCGCCTCCGCCGCAGCCATAACTACAGGTACCGAGTTCCCAACCCAATCAACCAAAATCC
CTCCCTAATTTCCCAACCTAATCCGTAACAATCATCGATGGGAAAATCACTATATATAAA
GGAGTTAAATTTGGGGATTAGGTTAGGGGTTAGCGATCGAGTCAAA

>RMYB25
GCCTAGAATCAGTCATGAACTCAAGTTGTATGTGACATCTCTTAGAATTATCGGACAATC
CTTTTATCTAAAAGGTTAAGTTGTTATTGGCCTATGTTCACTTTTGTCTAATTCCTCCGA
CATTAAGAGTTTTGTCCTGCATGTAGATCAAAAACTTAGAATTCTTACACTCTCTGAAAA
TTTGGGGCTAGGAATTGCGGCTGCCAATGTCCTGAATAATGGAGTCACATGAACAGGCGA
ACCAAATTCTGTATTTGAATAATCACACACTTCTTCACAGTTCTCTTCTATGTGGCCACA
TGTAAGCATGTTCACTTCGTTGGTGCTTGCAGTAGTATTGCAGATTCGACTTTCTTCAAC
AAATTCATTTCCAAGCTCTTGATGCAGCACACTGATATCAGTTTTGTGCTCTTCCATTTT
ATCAGCTGGAATGTTTGGCAGGAGAGTCGATCCTGTACTGTACTCAGAGCTTGCTGGGGA
TGATGCATACACATCAGGTTCCCTCCAAGACAGTCGGCTCCCTTCGTTGCTGCTTCTTAA
ATCTTCCACCAATTCTCTCAACTTCTCAAGATGAAAATCGTGATCAGGATTTTCAAAGGC
TGGCAGATTGTTTTCTCTGTTTTGCTTTAGAAATTCTTGGATTGCAATCCAGGCGCTTTC
AAGGCTCTGGTTTGTCTTCTCCGAGTTAACCTTGATCGCAAGCGAACTAAGCAACTCAGC
TTGTTGCATCAACGCAAGTACCTTCGGATCATCCTTCCTAAGGAAGGTTCCTTCTATTTT
GCAATCTTCAAGCCCATCCATCAATGCTTCCTTTATTTGGTTCGAGTGCAGCTCACTGGG
GATGCTAACCGCAGACGAATGAGCATTTTGTGCTAATACTTGAAATGGATGTCTCAGCAG
ATGATCTACGGCATCACATTCGCTGGATGGTCTCCCTTCTTGGCTTTCTACTGCATTCGT
AACATCGTTCCTCATCTTTTTAAGGGGAGCGCCACTTTTGTACCCTCCTGAATTGAGCCC
ATTTGGGAATATAACCCTTTTGTTGTTTAAGTTGATGTAAGAAGTAAAGTTGTTCTCCTT
TGCCAGCGCTTCGTGCTTTGCTTTCCTCTTGCATAGAGTGGCAAACCGGTTCTTCACAGC
ATTATCAGTTCTCCCCGAGACAACCTTTGCAATCTCGGTCCATCTGTTCCCAAATATCTT
TTGTGCCTCACATAGAAGCATGTCTTCCTCGGGCGACCATCCCCCTTTCTTGAAATCAGA
ATTCAAGTAAGTAAACCATCTCCTACGACATTGCCTCGCGGTTTTATCCTTGAATTTTGA
TGCAATAACAGCCCAATTTTCAATCCCATGTAACCTGATTTGCTCCCTCAAAATATCATC
CTCCTCTTGTGACCAAGAAACAATATGCCATTCCTTCCTCTTTGGAAAATCACCACTATT
TGCACCACCACTCTTATTATTCCCCTTCTTCATATTTTCCTCAACTTGCAACAAATTCCT
TTCATGACCCTTCTTTTCCAATAGATGTGTATTATATAACCAAAAAAACCAAAGGGCAAA
AACAAAAAAAAGAAATGCAAAAAGAAAAGATCAAGTCTTTTACCCCCTTTCCTCTTTACA
CAAAAGAACACTCACTCAGCTTAGCTCCCAAATCCAAAAATCAAAGTGTTGGTCTTTTAC
CCATCCCCTCAAGCTCAACACAAAATTTTTTGCCTAGGATTCCCACTTTTTTTCCCCCAA
GGCTAAATCCCTTTCTGAAACCAAACAAGTAAAAAAAGAACACTTGAAAACAAGGGGTCA
CTGCAGGCTTCTTAATTTGGATTCCACTTAGAGAAATTGGATGACAAAACCAGCCTCTCA
GCCCTCCTCAACCTCGAAA
>RMYB26
TGGGGGCGGGGGCAAGACCCAATGACCAGTTTGAGTTTGGAGTGAAGGGAGCTCAAGCTT
CGTTGCCTGGGCAGGCTCCGAAGCCGGGTTGCCATATAAAACAGCATGGTTGACGCTGCC
ATCGAGTAAACTTGAGGACATTATGCGATTGCAGGTTGGGTAACAATCATGTCCCGATGA
GAATACCAAAGGTTGACCAATCTGCAGGGGATCATCAAATGGACTCCCACTGGCTAATGC
ATTGCCTGTGGCAGTATTGAAACCTTGTAGCGGTGGCTCGGACGGTCGGAGGCGTTTGGA
AGGATAAACTGTTGATTGATGAGGCTTGTTTATGTTGGAAGAGTTCAAGTTCCTACCTTG
TGCAACTAGGCTACGCGCTGAAATATCGAGCAAGCTACCAGGAAGAATATTGGGCATTGG
TGCCGGATGAGTAGAATATATCGGACTGTTGAGCTCCAAAGCCTTGAAGTTGGGAATTTC
AGGGTTGCTGGCAAAATTGAAGCCCTGTTGGGGCACCGTGTTGAACAAAAAGGTATTCTT
GCTGCTGTTGTTTAGTTGGTTCTCCTTGGCCATTCGCAAACGGACATCAGGTGGATACGG
CGGCAAGCCAGAGCGTTCTAGTCTTTTCGTCCTAGTGTTCCAGAAGTTCTTGATTTCATT
ATCCGTTCGGCCAGGTAACTCAGCAGCCATTCGAGCCCATTTGTTTCCAAGCCTGGAATG
AAGTTCAATAACCTTGCGCTCCTCTTCTTGAGTGAATGAGCCTTTCCTTAGATCTGGTCT
GAGGTGATTAACCCACCTTAAACGGCAACTTTTTCCACAGCGAGCCAGCCCTGAGTGTTT
CTGCACTGCATTCCAGTTTCCCTCTCCGTGCCTGGTCACATAGTCGATTAATATTGCATC
TTCTGCAGAAGTCCAGGGACCCTTCTTTAGCAGAATGTTAGCTTCTCCTGTAGTAGCACC
TCCTCCACTGCTAGCATTATCCCTAGATGGCGGAAGAACTTCACTGCTAACCATCCTTTT

GTTGCTTCCGCTTGGCATACTCATACCCTGATTCGTCAAACTTGAATTTTGAACCAATCT
TCCGCTGAAAACAAAATCAATCACTCCCCCAAAAGCTCCAGATATCTCAAAGCCCGGAGT
TTTTCTTTATCTTTATTTACTTATCTGGGAAAAAAGTAACACAAAAAGCAAAAACAAGAA
ACCAAAAAGAACTCCAAAATCCGGCCTAAACTAAAAAGGGGAAGGAAGGACAACCAATGG
GCAATCAAAAGAAGCTTTGGTAAATCATGTTCCTCCTTTTCAATTTAGAAAACTGAAAAG
GAAATAGCAAAGAAAATCCAGGGAACCCTTTTGTGTGATCACAGGACACCACGATCCATT
TTTTTCCACATCCCATTAATCAATGCAACGAAAAAAGGGGAAAAAACATAAATAGATTTA
GATGGATAAACCACGGAATTGGAAATTTCTCAGATAAAATTAAATCATTTTCTTTTTTCA
AAACAAAAAAGGGGGAAAAGGAAAAACCTAATGTGGAGAATTAGATAGCAGGAAAAGAGG
AGAAATTCAGGGATGAACAGCACCCCACTTCTGAATTCATTTACAGATTAGCAGCTCAGA
AAACACACGCAGAAGCCGCAATTCACCCCAAATTCATTCACCGCCATTACACACACCAAC
ACCCACCCACACGCACACATATATACCTATGTATACATAGAGAGAATTTGATAAAAAAAA
ATGAGTAGATTGAAGTGAAATCTGCAAGGGTCTTGACCTCCACTGAGTTTTGTCTCTCTG
TGAAGAAGAGTGTGTGCGGTGTGTGAGATAGAGAAAGAACACTGAAAACCAGCTAGCAGA
GTGCCTAAATTTCAGGTGTTTATATAAACATATAGGGAGAGAGGAGAGAGACACGCATTA
T
>RMYB27
TCTTGCTGCTCCTCGTTGAATATATCATCATTAATCAATGAATTCAAGAAAGAATCGTCA
ATGCAGCAATTGAGCAAATCATCATCATCATCTTCATCCTCGTCTCCACGGATAATGATC
CGTTTGTTGCAGTTTCTCAGTTCCTCGTCATTGCTAATTGGATAATCCATATTGTGCCCA
CCCTCAAGTTGGTCACTGTTTGTGATCACATTTTCAAAGGGATTAAATGTTGTGCTATTG
TTTGGGATAGGGCTGGAAACTGATGATTTGTGTTCAAGTGCTTCTGATGATGAATCTCTG
TTGACGTTGACCGACGATTTATTTGGGTTGAATGGTTTGTGGGTTTTTGGATCTAAGCCT
TGCCTGATCAGCTTCTTGCACAGGTGAGTGTTCCAGTAGTTCTTTATCTCGTTGTCTGTC
CGCCCTGGAATTCTTCCTGCTATTAAAGACCATCTGTTTCCAAGCAGACGGTGGAGGCGG
AGAATGAGATCTTCTTCATCAGGGGTGATGTGTCCACGCTTCACAGAGGGGCGGAGGTAG
TTCATCCAGCGGAGGCGGCAGCTTTTCCCACAGCGGAGCAGCCCTGCCTTCTTCGGCAGT
GTTCTCCACCTTCCCTCACCTTCCCTCTTTATGTACTGCGCTAGAAGCTCATCCTCCTCA
GCCGTCCATGGACCCCTCTTCAGCCCTACATTGCTGCAATATGGAGTCACTGCCGCCTGT
TTCCCCGAGCTGCAACCATCACCATTGGCTGATGAGGTTTTCTTGGAGGAAGATGATGAT
GGGCTTCTCATTTGTTGAACTGTCACTCTCAATTATGCCTATCCTCTCTTTATTCCTTGT
AAATTTTAGTTCTCACCACCCTATTAATTAGGATGGGGCTTAATCTTCTGCCTTTGGTAT
AGGTTTAATTAAGATGAAATGATAGTGTAGAGAATTTCTTGATGACTTCATTTGAATTAA
TTGTGGATTTGGAAAGCTTACTACAACCAAAGAAAGCAAAAAGTGGAGATTATTTGAATG
AATTTTACATGAGAAATATGTTTCCTAAATTTGGCCTTGCATGGGGTTAAGAGAAGTGTG
GTTAAGAGAAAAGGCATGACTGTGAAAGTTGTTCTCTACCACAATCAGTTACTTGACGTT
CTTATTTCATTCACTAATAAGTAACAAACATGTGTAGTAAGACTTTTTTTCAATTTTTGT
GGCTAATTTATATAGCTAGAGCCTAGAGATTAAAGTTCAGAGGAGAGATG
>RMYB28
GCATGGGTAGAGAGCAAGACAACTTTACAATTTCTACCATAAAGTAGTCTCACCAATAAA
TACTTCCCTAATTCTGCAACACAACAGCTCTATACAAATGGATTAGATTATTAAAGTTTT
ACTCCTGCGGATTGATTATTCCCAAATTTCATACCAAAAATTACAAACAATTTAATTTCT
ACCAACATGTTATTGCCAACATTAATTCCTGTGAAGGTCTAATCACCTGAGCTAGAACCA
AGACCAGGACCAGGAACAAAACCAGAACCGGCGTCTTGTGCTTCCTTGGATCACCGATGG
GCGGAGGGGTGTGGTCCCGGGTAAGTCATAGGAGGCATGCTCAACCCGACCCCGGGAACC
ACGGATCCATGGACTGGAGCCCCAACTCCATACGCCATGTGCATGGGTGACGGGAGATTT
ACTGGTGTCCCCACTGCTGAGACAAGTGGTCCTCCTATGGGTTGCCCAATGGTTGGTGGT
GGCATGTACATGCTCACCCCGGCGGTAGCTACGGGTGCTTGAGGGAGGGGTTTGCTTGAT
TTCCCGGATGGACCGCCTGCTGATGAGCCGTTTGCTTGACCGGTGATGGGGCCTTGGGGC
AATGAGATATCTCCACTCGCGACACTGGTGATGTCATGGATGCTTGACCGTCTCCTGTCT
TTGTTCATCGAATTGAGACGGATGAAGTACTTTTGGGCATGGCTGGCCACTTGGGTTGGA

GTCCGTGTTACCACATAGTTCCGGGAAATACTCCTCCAGTCACCTTTTCCGTACTTGTCC
AAACCGAGAAGGAACAGCCTGTGTTCATCCTCTGTCCAAGCAATTCCCTTGCGTCGTTCT
TGATCTGATTTCGAAGACTTGCCCCCGTGGTTGGATTCCCCATTGGACTGGCCCGAATTG
GCCCCTTTCCTGCTGGGTGTTCCATCACCAGCATGACTAGTTGAGCCATCTGAGGCAGAA
TTGTAACATGGAACCTCTACAAAACCGGATTCTATCCGTTGGACATCGTCGACTAGCACC
TCATAGTGTTGCTTGACCTCCTCCACTGATTTTCCCGGGACGTCAGCTGCAATTTTTTCC
CATCTATCCAAAGTGTCTTCGGGATAAGTAGCAATCGCCTTCTCGAACTGCTTGTCCTGC
TCCCTGCTCCAGACAGAGCTGCTCAATTCACTGGCAGACGGCATCTAGTCCTGTCAGAAA
TTCGACAAATTGTCCGAAATACAAACTGAATCCTAATGAGGTTAAAAATTCAGGGAGGAT
CTAGAAAGGAAACCAAACAAGAGCAGCTCAGATAGATATGTATAATGCTGTCCTGATTGA
ATTCGCCGGCGAATGGGCACACAATGTCAAGACTTTGAACAGTTGAAAAAATGTCGAAAA
CACACAAACAAGTATGGTTAATAATTAGAGGATGAGTAACATCTGAAAAGCACAGCACAG
CACAGAAAAACACCCCTCTCGGGACCTCAAGAAATTCCACAAATTCTCAACAGTGTCGCC
GGAGAAAGGGAATCTAAGGACAGAGATGGACAACGGCGATTGGCAAAGGCGAACGGGCAC
GAATCGGGACAGCAATAAGGGGCAGTACTGTGTGGGAATGAATGTGAAATGTGAAAAGGA
CCACCATGATAACAAACAAAGAAGAAAGCACCAGAAGTCCAAAATGGGGCAATAATACCA
ATTCAAATTGCCCACACATCCATCACTACTCCTATCCCCCACACAAAAAAAACTTCCCCT
TTTTTCACCAGCCAAAA
>RMYB29
CTGCTCCTCGTCTTCATCAATTCTGCCGGCAAAATGAGGATTATGATCAAGGGTGGAGTA
TGGAAGAACACGGAGGACGAGATCTTGAAGGCGGCGGTTATGAAGTATGGGAAAAATCAG
TGGGCCAGAATTTCTTCGCTCTTGGTTCGAAAATCTGCGAAGCAATGCAAGGCGCGGTGG
TACGAGTGGCTTGACCCCTCCATTAAGAAGACTGAGTGGACTCGAGAAGAAGATGAGAAA
TTATTGCATCTTGCAAAACTTATGCCTACTCAATGGAGAACTATTGCCCCAATTGTTGGG
CGAACTCCATCTCAATGCCTGGAGAGGTATGAGAAACTCCTAGATGCAGCATGAACTAA
>RMYB30
CAACCAGTAAGTGTTTTGATTCAGTCGTATCATTTAGTCTGAGTGCTTTCAGATTATGTT
TTCAAAGCCCACTTAAATGTCTTTGCTTTGACAAAGGATCAGATAAGGAAGAGAGCTTAT
ATGCACACCAAATAATATTTTTTATATCTCCACTCCAATAAGTTTAATCAAAAAAGTATT
CCAAACACCATGGAAAACTTATTGCACATTACACTGCACACTGGAACACAGCAAAATATA
TATTGCTCGGATCCGCTTTTGCTCTCTAATCACTTTCATCTATTCTATTTCTAGTTATAA
TATCTGTAAGGGGTAATCATGGCGAGGTGCCACTGTATAGTTACTCATAATGACTAAAGG
GTTTGTTTTGTTGGGTTGTCTGTACATAGGCTTTTCTAGCCAAAAGGTTTGGGAACACTG
GATAGCCGTATCCAAGTTCCATGATGAAACAAACTAAAAGAATGGCACACCAAGGAAACC
ACATAGGGAAAAAGGGGACTAATACGATGAGAAGACCACAGCTAAGATCGCAGCAGTTGA
TCTTCAGAAGAAACATTTGGGTGCTCCTTACCACCATAGATTCCAAAGCAGATCATAGCA
AGCGAATTCAGAAAAACTCAGGGCGAGAAAGCTGCAATCCAAGGGTAACAAACTCCAAGA
AGAATCGAAAATGGTCTATACTGATGAGAACACCAGATATATATTTGTAATTATTCGAAG
GACTGCAGGAGATTGGTCATAGACAATGGAGGAGTGACCTCTTGCAGGGTGGAGGTTGGG
AAGGATTGGTCATTGTGCTAGGAGCAGCTGATTCCATTCCTAGTGTCTTCATCAAGAAGT
GCTTTTCACTTTCTGAAAATTTTGGGCTTGGAATTGCTGCTGCCAATGCTCTGAACAAGG
GAGTCACATGGAGTGGGGAACTGAATTCTGTGTTAGAATATCCACATGCGGCTTCATTAT
TTTCTTTAGTATCATTGCCTGATACCATGTTAACTTCATTTGCAGTTGCAGTATGGCACA
TTTCACTTTCCACGGAAAGACAACACTGATCATCTACTGGATTTGATTGCAATTCTTTTC
CAGTTTCCCAGTGTAGCACACTGATTTCAGATTGACATGCTTCTATCTTATCGCCAGGCA
CACTAGACAATAGAGTGGAACTTGTGCTATAGTCAGAGCTGCCCGGGGATGACTCACACA
TATGAGGTTCCCTCCAGGAGGACTCGCTGCCTTCATTACTGCTCCTTAAATCCTCCACCA
ATTCATTGCAGTTTTCAAATTGAAGATCAATATCAGGATGAGTGAACATGATCAAGTCAC
TTTCTTTGTTTTGCTTCTGAAAGTTTTGAAGGACCATCCACGCACTTTCAAGACTCTGAT
CTGTCTTCTGTGAGTTAACTTTGATTGCAAGTGAACTGAGCAATTCTGCCTGTTGCATCA
ATGCAATCACCTTTGGATCATCTTTCTTTAGAAATGTTCCGGCACTTTTGTTTTTTTCTT

CAGATCCATCTGTTGATGCCCCCTTTATGTGATTAACATTCATCTGAGCCGGAAAGTCAA
ACTCATGCACACGGGCAATTTGGCGGACTGTTGCAAATGGATGTCTATGTAAATCATCTG
TAGCATCACACTCACCAGCTACTTTCTCTTCTTGATTTTCAGCTGCTGTATTTGAGATGT
ATGTCCTAATTTTTTTAACAGGCACAGCGGTTTCAGATCCTCCTGAGAATATAACCCTTT
TGTTGTTCGAATTGATGTAGGAAGCAGTGTTGTTTTCTTCTGCTAATGCTTCGTGTTTTG
CTCTCCTCTTGCACAGTGTTGTGAATCGATTCTTCACAGCATTATCGGTTCTGCCCGAGA
CCACCTTTGCAATTTCAGTCCATCTGTTCCCGAATATCTTCTGTGCCTCACATAAGAGCA
TGTCCTCCTCAGGTGACCATCCTCCTTTTTTGAAATCAGAATTCAAATAAGTAAACCATC
TTCTTCGGCATTGTCTTGTCGTTTTATCATTGAAGTTTGATGCAATAATTGCCCAATTTT
CAATCCCATGTACCCTGATTTGCTCCCTCAATATATCATCCTCCTCCTGAGACCAAGAAA
CAATATGCCTTTCCTTCTGCTTTGGCACTCCACTATTACTAGCTTTAGTGCTGTTATTCA
CCTTCTTCTTCATACTCTCTGTAGCCTATAAATGTCTTCCCCTTTGGCATGCTCAAGGAA
AACACTTTTGTCTTTTGAGAGTGCTTATTATATAACAAAGAAAATTTAAAAATAAAAGAA
AAGAAAAGAAACTATGCCCTAGAAAAAAAGATGAATCCTTTTCCCTTTACAGAAAATGCG
TAGCTCCAAAACGGAACAGAACTAAAGAAGGGCATCAAGAATAACACCAAACTGCTGTTG
GCCTTTTACCCTCCCCTCAACCCAAAAACTTGCCTAGGATTCTGACCTTTCCTCCCACTT
AGACAAAACATCGAACACTTTGCAAGCAAGAATCAACCCTCAAAATTGCCTCGAGATTTG
AGAACAAAGGCACAGAACTTGCGATCCAAGAATATAATGGGCTATTATCAAACAAAAATA
AAATAGTAATCTCTCATTCACGGGATTCAAAAGATGGGGGAGATGCTTTCTTGGTGTCGA
AAAGTGGGAGGCTTTCAGAAAGAAAAGATGCAAATGTGAAATGTGGGGGGTGGTGGCGTT
GATTTAAACATATTTTAATGTGGTATATGTAGACTAATAAATTCCCACAAATTCAAGAAG
CAGCAAACGGCAACGGATTCTTGCTTTTGGGCGCGGGTAAGAAGGGAAAGTTTGAAAAAT
TATAAACAAATGGTAAGAAGAATAATGATGAAAAAGGAAAATTGAAAAAAAAAAAAAATG
AAAGGGTAAAAATTGCTGAAACCCATGGGAGCATAATTAATGAATGCAGCGCTGAGTGCA
GGAGAAGGGAGCATAGAGTAACCCCACACACCCACGACGCGCAGTGTCACACACCCACT
>RMYB31
CCCTAGTTAAAAATTGGGATTTTTATCCCTTTTGTACTCTTGATTGTTAGATGGAAGGAG
GAAACTTGACAAATCACGCCCAAGTGCCCCAAAATACTAATAGTATCTTTGAATTTGCAC
TAGTTACGTTGCAACATACTTTGGTTTAAAAGTATAGAGTTGATTTGTATTAGTATATCA
CTGTATTGGAAATATTCATAAGGACTAAAGTGCAAATGTGCAGTGAAGAAGATCCTCTTC
CGTAGTAAGATGAAACCTGCACTGGCCGACTACATATATATATATATATATCTATATATC
TACATATATGAAGCACTAATTAAGACGTGTTTACCTATTAGTAATGCTCATTTTGCAATA
TGAGGTGCAATGCAAGGTCAGAGGATTACCATACTAATTCATGAGTGATATGGATGAATT
AAGATTTAATTGGGAAGAGGCTTCAATTCCCTGCACGCCCCACTGATAAGAAATCTATAA
ATGGCAATGCCACAACTCCTCCTGCTGCTGCTGTGTTTTTAGTTTCCCCAATTGACCCCT
TCTGATCCACTTCTTCTTCTTCTTCTTGATGCTCCTCCTCTTCAATATTTCTCTTTGCAT
GCTTATGATTATTGATCACTTCACTTCTGTTTGAGGAGTCGGTGTTCACTTGCAGAAAGT
TGTAGAACTCTACTTCCCTGCTTCCATTTTCTTGAGCTATCTTGACATGCTGACGATCAT
TCCACAGTGGCAGCATTAACGCCGATGGTAAATTATACATGGAAAGAGGATTGCTGATCT
GCTGATGAGCAAATCCATCCAGAAACGTGTGCCCCAGGCCAAGGGTAGTGTAATTATTAT
TAATGCCTAAATCCTTCCTTGCAGTACTTGTGCAATTAGTTGGTGCTGCTAAGACCCTGC
TATTGTTATCACATTTGGATGAAGACGATGATGATGATAATGTTAATGAGAGTGCTTCCT
TCTCATGAGCATTGATTCCTGATCTCCCAATGATGGGCTGATTCTTTTGATGACGACGAT
GGTAATGATGAGATAAAGTTCTCTTGGCATAGAATTTCGACCTCTCTCTGCATTTTCTAG
CCATGATAACATGCCAGTGGTTCTTCACAGCATTATCAGTGCGCCCCGGGAAGAATCTTG
CAATTACAGCCCACCTGTTACCATGAATGCGGTGGCAGGCGAGCAGACGCTCTTCTTCCT
CCTCGCTGAAAGGGCTGCGGTTGATTCTCGGGTCTAATTGATTAAACCATCTCAGCCTAC
AGCTCTTCCCTGATCGGCCCTGGAGCTTTTCAGCTATGGCGTTCCAGTTATGAGGTCCAT
ATCTTT
>RMYB32
GTGGATGATTGATCGTTTTCCGTGTAAGGTTTATGCTGATGCGTTATATCTGTAAAATAT

TTTTTGTTCAGTCTTCATCGGTTTTAAATCCATGTGAAAGAAAACTCATGTATACAAATA
AGCTGAGGATTTATTACTACAAATTTCCATGTCTTCCCCTATTCACCCCTTAATCCTTCC
ATTCAAACACAGCTTTTTATTTAAGAGATGCTAAGCAAGTTCAATCCAAAACCTCGTAGA
TTGCAAACGTTGAACTGCACATTTGACTCCTTGTTGGCTTGTCCTTAGGTAAACAGATTT
CACTATTTCACCAAAATAAAATAGAATAAAATAAAAATAAGATCCTGTGCGCATTGGTAT
CTCGCCCGGAAATACCAGTGAATATCCCATGACCAAGTGACACAGAATGCTCTAAACCAC
CATAGTCCTATACACATGGATGCGGTAGTGTTTCCTGTTTTGTTTATTTAAATTTAATAA
GAATTATCATTCCAACACTGCAAAGGACAATGCATTGCTACAAAAGCACAGCACCATCCA
TGTAGTTTTCCATCTTTGTAGCATCTTATTCAGAATCACCTGTGAGCAGATGTATGTGGC
ATTGGATAAGTTGTTGGTGCACCGGGAACGACGGGAGCTCCGACACCATAGGTGGCCAAG
TGAGGGGGCGGCGGAAGACTCACCGGTGTTCCAAGTGCAGAAACAAGGGGTCCACCGATA
GGCTGTCCAATAGTTGGTGTCCCGTACATGTTGACACCAACGGGGACTTGAAGAGACTGC
TTGTTCGACTTTGGAGATGGGCCTCCTCCAACAGCCATACTATTTGTTTGGCCAGTGATT
GGGCCTTGGGGCACGGAAACATCTCCATTGTTGACACTTGTGATGTCATGAATGCTCGAC
CGCCTTCTGTCTTTGTTCATCGAGTTCAATCGAATAAAGTACTTTTGAGCATGACTTGCT
ACTTGCGTAGGAGTCCTTGTCACGACAAAATTCCGAGAAATGCTTCTCCAATCACCTTTT
CCATATTTCTCCAAACCAAGAAGAAATAACCTGTGTTCATCTTCTGTCCAAGCAATCCCC
TTGCGACGCTCCTGTTCTGACCTGGAAGATTTGCCTCCATGGTTAGAGTCATTGTTCAAG
AGCCCGGAATTGCCACTTTTCTTACCAGCTCCTTCATCATCAGCATGGCTTGTCGAACCA
TCTGAAGAAGAATTATAACAAGGAACCTGTATAAATCCAGATTCAATCTGGTTGACATCA
TCAACCAAAAGTTGGTAATGATATTTAATATCTTCCACAGACTTCCCGGGGACTTGAGCC
GCAATTTTCTCCCATCTATCAGAAGAATCCTCAGGATAAGTAGCTACTGCTTTCTCAAAT
TTCTTATCCTGCTCCCTACTCCACACAGAGCTGCTTGATTCATCAGCCGGCATCTAGAGT
CACTTTCAACCAAATACGGATTCAATACTATCCTCAACCAAAGAACCAAACAAAGAACAC
TCCACCACTTCAAAAGCTATTAATCAATTGTTCAAAATCCTAAAATTCGTTTGGGATCTG
GTTTATTTACTTTGAAAGACACCATAAAAGCTCAGTTTAGCACACCAATTGCAATAAATA
TCCCAAGCTTCACAAATTCAAGAATTGGAATGGGGTGAACTGCTTGCTTACCCTATTGAT
GATGAAAGGGAAATATGCCTGGTTAGAGCATGAGAAATATCTGAAAATCATAAAAATCCA
GGCTTTACAATAAATTTAAAAAGGGGTGAAACAAATGGCCATAAATGAGAGTGAGGAGCA
GAAAAAGATGGCCGGGGGTGGGGGACGAGAATAGACAGATTTTAATTGGTGACGGTGAAT
TCAATATGAATATGATGAGAGCAGTAAGGGCAGGCAGCGTGAAGGCGAGGAGGATGTGAG
ATGTGAAAAGGACGGCCTTGGTACCAAACAAGGAAGATAATCCCAGAGAGCTGAAGCAAG
AATGGATAAAAACAAGATTGAAATCGCCATCACTCATCCCAACAATTTTTACCCATTGGG
CAGAGGCATAGAGAGTAGAGACCCATGTTTTGATGGCAGCCGCAGTTTTGACGACAGCAC
AGACAGACAGGCCCACCGCCCACAAGAAAGATGAGAGTATATGAATTTCGCCCTCTAGAG
TCCAGCCT
>RMYB33
ACAGGGTTGTCGAGATGCAGCAAGAGCTGCCGGCTCCGGTGGACCAACTATCTCCGGCCA
GGCATCAAGCGCGGCAACTTCTCCGAACACGAGGAGAAGATGATCATCCATCTCCAAGCC
CTTCTAGGCAATCGATGGGCTGCAATAGCTTCCTATCTTCCCCACAGGACCGACAACGAC
GTCAAGAACTATTGGAACACTCACTTGAGGAAGAAGCTAGGGAAGCAGCAGCAGCAACCC
CAAGGCCATGTTTATGTGGATGGAAATAATAATTCTTCCCGTTCAAAACCGGTTTCCGTT
ACCAAAGGACAGTGGGAGAGGAGGCTGCAAACCGATATTCAAACGGCCAAACAGGCCCTC
TCCGAGGCTCTCTCCTTGGATAAATCCGATTCTGCAGCCGTTGTTGATCAGCACCCAAGT
CCAACGACCCAAACTAGGCCAATTCAGACATCTATGTATGCAGTAAACGCGGACAACATC
GCCCGGTTGCTGGAAAACTGGAAGAGCAATGACTCCCCAAAACTGCACCAGGGCGCC
>RMYB34
CGGACGGTCGGAGACGTTTGGAAGGATAAACTGTTGATTGATGAGGCTTGTTTATGTTGG
AAGAGTTCAAGTTCCTACCTTGTGCAACTAGGCTACGCGCTGAAATATCAAGCAAGCTAC
CAGGATGAATATTGGGCATTGGTGCTGGATGAGTAGAATATATCGGACTGTTGAGCTCCA
AAGCCTTGAAGTTGGGAATTTCAGGGTTGCAAGCAAAATTAAAGCCCTGTTGGGCCACCG

TGTTGAACAAAAAGGTATTCTTGCTGCTGCTGTTTAGTTGGTTCTCCTTGGCCATTCGCA
AACGGACATCAGGTGGATACGGCGGTAAGCCAGAGCGTTCTAGTCTTTTCGTCCTAGTGT
TCCAGAAGTTCTTGATTTCATTATCCGTTCGGCCAGGTAACACAGCAGCCATTCGAGCCC
ATTTGTTTCCAAGCCTGGAATGAAGTTCAATAACCTTGCGCTCCTCTTCTTGAGTGAATG
AGCCTTTCTTTAGATCCGGTCTGAGGTGATTAACCCACCTTAAACGGCAACTTTTTCCAC
AGCGAGCCAGCCCTGAGTGTTTCTGAACTGCATTCCAGTTTCCCTCTCCGTGCCTGGTCA
CATAGTCGATTAATATTGCATCTTCTGCAGAAGTCCAGGGACCTTTCTTCAGCAGAATGT
TAGCTTCTCCTGTAGTAGCAACTCCTCCGCTGCTAGCAATATCCCTAGATGGCGGAAGAA
CTCCACTGTTAACCATTCTTTTGTTGCTTCCGCTTGGCATACTCATACCCTTGAATTTTG
AACCAATCTTCCGCTGAAAACAAAACCAATCACTCCCACAAAAGCTCCAGATATCTCACA
GCCCAGAGTTTTTCTTTATCTTTATTTACTCATATGGGAAAAAAGTAACACAAAAAGCAA
AAACAAGAAACCAAAAAGAACTCCAAAATCCGGCCTAAACTAAAAAGGGGAAGGAAGGGC
AGACCTATGGGCAATCAAAAGAAGCTTTGGTAAATCATGTTCCTCCTTTTCAAGATTTAG
AAAACTGAAAAGGAAATAGCAAAGAAAATCCAGGGAACCCTTTTGTGTGATCACAGGACA
CTTGGATCCATTTTTTTCCAAATCCCATTAATCAATGCAACAAAAAAAGGGGAAAAACAT
AAATAGAGTTAGATGGAGAAACCACGGAATTGGAAATTTCTCAAATAAAATTAATCATTT
TTTTCCAAAACAAAAAAGGGGGC
>RMYB35
TTTCCCGAACTTATTTGACCGTATCATCCATAATCAGCTTCAGGATATAGCATCAGCGTT
GGGTAAGATACTAATAGTCTCGGTAGTTGATTAATGAGCACAACATGCTTTTATACAGTT
AGTCTCCCTGCATTCGGGTTGTGCTTCTTGACACTTTTGTGCATAAATTGGCATGCGGTG
TCTTTAAAGTATCCGTCTAGTGTTTCTCTATGTTTGTACATCTTGTGTGTGGGGGGAAAA
AACTAGATACGGGAAATGTTACAGAATGAACTTATATAAACCTCCTTTTCATAAATATTG
AATAGATTGTTGTATAGATTTGGGCGCTACTCAAGTTAATTTTCGTTAATTAAATATTAA
TAGCTTTAATATATCAAACACACATTGATATAATCCCCACAATAACACTCCCTCAAAATA
CAATTTCAATCACAACACCAATCTCTCAATATAACCAAACCAAACCATCACTCTTAGAAA
ACCAATCAAACTATTGAAAACCACATAAACATGATGAAGACTTGGCCTAACCTAACCAAG
ACAATACCAAATCATCAATCAAGGTTAATCTATGCATCCTCCTCAAGCTCAAGAAAGCTC
TTCACCCATTCCAATCCACACGTCCCCTGTCTCGAGAAATCGAAGAGCTCATCTCCCTCA
ACATCGGAAGAACCCATTCCATCCCCCACACTCATCCGCTTCGTCACCATCTCACTCCCA
CTTGCAATCTCCATTGATGAGGATGCTTTCTCACGTTCTTGATCATCGTCATCGTCATCG
TCATCATCGTCATTGTCCTCATCCCATTTCTTGATCAGTGCCACCGTTGATGTGGACGGC
AGCTTCTCCATCAGCTTGGCCTTCTTCCTCAGATGCGCATTCCAGTAGTTCTTTATCTCG
TTGTCGGTTCTCCCCGGAAGCCTCCGGGCTATCAACGACCACCTGTTTCCCAGAAGCCTG
TGAAGCCTGAGTATCAAATCATCCTCCGCTTCGGAGATGTTGCCTCTTTTTATGTCTGGC
CTCAGATAATTCAACCATCTCAGTCTACAACTCTTCCCACACCTATTCAAGCCTGACTTC
ATGGCAACGGATTTCCACCTCCGGGGGCCGTGAATCTCTATGAATTTGGCGAGTGTGGCG
TCTTCTTCGGGAGTCCAAGCTCCCCTGTTCAGTGAAGATGATGATGATGGTGATGATCTC
TTCCTGGGAAGAGCCCCATCATCGTCGTCGTCGTCATTATCATCATCATCGCCGCCATTA
CCATCGTGATCATGAGGAAGATGGTGGCGATTCTTGTCAGCCATGGGATTGGGATTGGGT
GGGCCTA
>RMYB36
TGGGTCGATGCCCATCCGTAGAAGGCGTTTCCTGATGTGTGTGTTCCAGTAGTTCTTGAT
CTCGTTGTCCGTCCTCCCGGGCAGCCTAGCTGCTATGGCTGACCACTTGTTACCAAGGAT
GCTGTGGAGTTGAATAATTGTTTCTTCTTCCTCGAACGAAAATCTGCCCCTTTTGATGTC
CGGCCGGAGATAATTCGTCCACCGTAGACGGCAGCTCTTCCCGCACCGCTGCAGCCCAGC
ATTCTTGGGAAGGGTTCTCCAGTTTCCATATCCATGTTTCTGAATATAATCAATGAGCTT
CTGGTCTTCTTCCTGTGTCCATGGCCCTTTCTTGAGTCCATTTTTGGCACAGCAAGGTGT
TCTTCCCATGGCATCCAAATTCAATAATTTTACCAATTAAGCTGCTAATGATAGATGTTC
TCTTCTACTTACAATTACTTTTGAGGCACATGCAAATGGCAAAGTGACAGGCTAGAAATT
GGTCCGTATATATATAGGTGC

>RMYB37
TCCACTTTGGCCTGATTTCTCATGACCCTTTTCTAGCTTCTTCTTCAAGTGCGTGTTCCA
ATAGTTCTTGATATCGTTGTCGGTCCTCTGAGGCAGGTACGACGCTATGGCAGCCCATCG
GTTGCCGAGAAGCGCTTGAAGGTGGATGATCATCTTCTCCTCATGGTCAGTGAAGTTTCC
CCGCTTAATTCCCGGCCGAAGATAATTAGTCCACCGGAGCCTGCAACTCTTGCTGCATCG
AAGCAACCCTGTATTGGCCGGAACAGCTCTCCAGTTCCCAGGACCATGTTCCTGAATGTA
AGAAACCAAGACGATGTCTTCTTCCGGCGTCCACGGCCCTTTCTTCACCCCTATTTTGTC
ACAGCAAGGTGGCCTTCCCATGTCCCAAAATCTCTCAGTTTTTCTTTAAGAGAAAGGAAA
GGGCTTCAAGATTGATTGTTTTTCCCTTGGCTTGTAGTATTGTTGATATATATGATGAGA
AAAATGAAGGGAGACAAGAGTCTAAGTGAGAAGAGAAGGGAATGGTGTAAGGGAATTATA
TAGAGAAGAGACCAAAAAGGAGGTTAT
>RMYB38
TTTCTCCTCTTGAGCCTTTTTTCTCATGTGAGTCTTCCAGTAGTTCTTAATCTCGTTGTC
CGTACGACCAGGTAATTTCTGAGCAATCTTTGACCACCTATTTCCCCATTTGGCGTGGAG
TTGAAGAACAAGGCGTTCTTCGTGAGGGGTCATCTTGCCCCTCCTGAGGCCCGGGTGAAG
GTAATTCACCCAACGCAGCCTGCAACTCTTCCCTGATCTTCTCAAACCTGAAACTTTGGC
GATGAAATCCCATCGCCGGTCGCCAAACAAGTTCACGTACAACACCAGCTGAGCATCTTC
TTGTTCTGTCCAGGGACCTTTTCTCAACTCATCCTTCTCCATATCCATGTCCTTGGGTTA
ATTCTCTCTGCTTCCTTCCTTCTGTGGCCTTAGCTACTAGCTTCTTGTCGTTTCTTGAAT
GATCACTTTATGGTTGCCCTGGCCTTTTTATATATATGTGTGCATAAAGATTCCTTTAAA
CATTTGGCAATATGTTTGTGGGGGAATTTGTGTAATAATGTAATGAGCATATGATTGGGG
AATCGTGGCCGTACATTTGTTATTTGCGCGTGGATTCATCTTCTTTCTTG
>RMYB39
CTATGGCACCTGTATGAAAAATATAAGTGTGTTCAACTAAATAGTATATGCATACCCAAA
TATAAAGAGTGCTAACAAATTAAAGGATACAGTACACCATACTCGAGCAAAGTAGTTCAA
CCAAATACATAGAGATTAGAGAACCATCCAGCCACAAGGGCTTCTGCAGATTTGAAGGGT
TCAGTTTCTACATAATCAAAGCCATTATTGAATCCTCCCAGAAAGCATAACAGTTTTCAG
TATATAGTCGAGGATACAAGGCAGCACGACCACCACGTTCCGATGGGGTACCTAGACTAG
ACAATGCAGGATCGCCTTTACCGGCCATTAGGTTCAGCTGCAGCAACCGATCTGCATCCC
ATCTGCTCGAGAAACTTGGTCAGTCGGATGTGCTTGGCTGCCCATTGCTCAGCCAGTTTC
TGCTCTTTTGCCTCCGCTTCCCTCCTCAAGCTTGCCAATTGTTCTCTGTACTCAGTTTCA
ATCCGTTCCAGTGTAGCCTTCTCCTCTTCCCTTAAAGCTTTCACTTTTGCTTCAATGTCT
TCCATTTTCTCCCTCCTACGGTTCGCTTTCTCTGATTCCAGCTGCAGTTCGACCCTTTTC
AATCTCCACGCAGCTTCTTTCTTTTGTGCGGCCAGTGCGCGGTGCCCTTCTTCCAATTCT
TTACAGCACTCAACTAGTTCAGAAGCGACTGTGCTCTCCCCAACCATAGGGGTGATCCCA
AGTGAATGTATATTGTTCATCATCCCGTGTGTTGCGCTATCAGCTAATCGATCGGGAGGT
TGTAGCCACGGGATTGGTGGTGTAGGTGGCACAGTCAGGGGAGAAAGGCTTAATGTCACC
GATGGGGAAGGCGGCCTGACAGTCGCGGTCGTGCTGGAACTCGCTAGCCATGGAGGCAGC
ACGGAGGGTGCCGAGGGGGCAGTTTGTTCTCCGTGGAGATATCCACCATTGGAAGCCGGC
ATTGTGATCCCCGGCACAATATGCTCCCTGACAAGCTTCTCGGCAAACGTCTCCAAAATA
TGATCGTACTTCCCTTCTTCAATCGGCTCAATTGTCTTGTTATTCTCCTTCTGCTCGCGC
TGCTGCTTTTCTTTGAACACTTCCCACCACTTCCCGAGCCTTTTGGCGGTTCGACCAGGG
ACTTCAGCCGCAATTTTTTTCCATTTGTTGCCGTGCTTGGCCTGCAGACGGATGACAAGT
CGCTGCTCCTCTTCAGTTAGTGATCCTTTCTTGATTCCGGGTTTGAGATAGTTCTTCCAC
CTCTCTAAACAGGATTTCGCATCCCGGTTGAGGGGTTGGTTCATGCGCTCTGACACAAGG
TGCCAATCTCTCGGTCCATACTGCTTCACATACGCACGCAACAAAGTGTCCTCTTCAGGT
CGCCAACGCTGCCTTTCCTTCATTTGTCGACGCGAGCTCTGCCCATTGCCTCGAACTCTT
CATAGCTATCTGATAACATAGTTAGAACAACAAAATTGCAGAACATAAACTACATTGCAA
GATCAGTTATTCACAGGATCCATTGAATACTTATGACCATTGCTAAACAGGACATATTCC
CAAAAGCATTTCAACTTGACGGAACCGGACTGTATAAACTGATGATTAATTATTTCATTA
CAGGACATTCAATTGACTGAATAAAAATATCCATAAAAGAAAGTAATGGATAAGGAGAAG

AGTAGAGATCTGGGAAATCTTTAAGAAAAGATAAATTCATATCAATTTTTCATAATTCAA
GCAAACCCTAATGCTATATCAAAATAGGAATATGTATATATAATAAAACAATACGGTATG
ATTGTCACATCTCTGGTGATGATGAAACCTCTTCATTCTTTTCAATTATTTAATTATCAT
TCATATAAGCCAACATGCCAACAAAGTTAACATTTGCATTTAATATTCTTCTCAGAAACC
AAGGTTACAAACATCCACACAATAAACTAATCACCTCAAGGAGACAAGACAGCAGTATAT
TAGCTTCACATTTGTCTCCATATATGTTTGCAATAAATTATGCAAGAAAGAAGGGGGGAT
GAAACAGGGGCAGCTATCTGGAAACAAGGCAAGAATGAAAGGGGGTGTCACTTTATCTTC
TTAATAGAAAAACCAAAAAAAAAAGAACACAAAGGCACACCAAACAAAAGCTCAAATTGAT
TTACCAGAGAGAGATATCCCACAAAGA
>RMYB40
TTCCTTCTTTTGCCTCCTCTCTCTCTTGTGCGGGCATTTTGTTTCTTTCGTTTTAGACCC
TTGTTTGCCAGATCTTTCATATGCTGAGGGGGGAAAAGGAAAAGAAAAAGGAAAAGGGGT
TTCTTTTTTATTTGTGAAGACAGCTCTTGTTCGAAAATCAAGAAATGGGCTGTGGAGAGA
CCAGGATTTCTTGAACATTACTATAGCTATGAAGAGTTCGAGGCAATGGGCAGAGCTCGC
GTCGACAAATGAAGGAAAGGCAGCGTTGGCGACCTGAAGAGGACACTTTGTTGCGTGCGT
ATGTGAAGCAGTATGGACCGAGAGATTGGCACCCTGTGTCAGAGCGCATGAACCAACCCC
TCAACCGGGATGCGAAATCCTGTTTAGAGAGGTGGAAGAACTATCTCAAACCCGGAATCA
AGAAAGGATCACTAACTGAAGAGGAGCAGCGACTTGTCATCCGTCTGCAGGCCAAGCACG
GCAACAAATGGAAAAAAATTGCGGCTGAAGTCCCTGGTCGAACCGCCAAAAGGCTCGGGA
AGTGGTGGGAAGTGTTCAAAGAAAAGCAGCAGCGCGAGCAGAAGGAGAATAACAAGACAA
TTGAGCCGATTGAAGAAGGGAAGTACGATCATATATTGGAGACGTTTGCCGAGGAGCTG
>RMYB41
ATTTTCTTCATTTTTTAGTTTATTACGGCCGAAAAATAAACAATTACCATAAAATGAATG
ACTCAGATCACCTAATCCTTGTCGAAACTGAGAACCACATTGTCATCAAATATGTCTTGT
AATGGTGTCTGGTCTCCAATTTGGTGCCCCATTTCGGTTTGGGATACTTACAGTGTAAGT
TATTTTAGTGGACTGTTTCTATTCCGGACCATTCTCAATGTGTTCACACAAACAAGTAAA
ATCAAGAAATTGGAATTAAGCTGAGAGCCCCATAACGATTATATTGCTGCAAACAATGAC
AATAGTTGATAGCCCGAGTGATCTATCCATAGACAAGAGTGGAGTGATAGAAAATGTCTT
ACCATTTGAACTCCTTTGCAATTTTGAAACAGATTTGGTTTTGCCAATTAATGTAGTTAA
AAAATGTGAAAGGAAGAAGTGAACACTGAAAAGCGAAGAAACAAATTATACTTAGGGGAC
TGACCTCAGTCCAAAATTCAGATTTGTAAAACCATTGACCATATTTAGATATTCCATTGT
GTAGATTGACTGCTGCTTTAGTTTTCAGAAGATGAGAAAAATGATTGTCGAGTAAATCAG
GCAAGCAGTTAATGAGAAAGTGCAAAATGGTGATCTCTATATTTAATTTTTATTTGGCAT
CTCCTAAGTCGTTTGTTTTGAAATGAAAATTTTTCAAGTGCAACATCGATTCCCCACTGG
ATTGGTGGTTGGGCTGAGTGGTCAGTGATCACCACTTTCCTCTCATCTATTTACAGTAGC
CAAACTTATTCCTCTCTGGGACTCTTCCATGGCCATCTACCATTCAATTTTCCTAAATTG
GATCATCAGCTCACACACAGACTTCACTCTACACTCCATTTCACTTCACCACCGCATCAT
TCCGAATCGCGTCATTCTGCACTCCGAATCCATTCTGCTCAATGCCCTCCATGTAATTCC
TCACCTCCTTCCTCACCATCTCCATCATCACCGCCAGGAAATCCGGGCTGAAGAACTGAT
TGCTCACCGCCGAATCGAACTGCATCATCGCCGGCGACGTCATCCCAACAGCCACAGCAG
GATCCACCGCGTTCGGATTCGGGTTGAACCTTGGCGGCGACTGATTTAGCTGCTCCGTCA
CCGTATTCGTATTCGGGTTCGGATTCAGATCCGACCCGGGTGGCGAAAGGCTGAGCGACG
TAACCGGGTCGGGTAATTGCAAATCGGGTGGAGAAAACCCGCCCGCTCTCGCAATAGGCC
GGAAAACCAGGTGACCCGAATGGCTCACCCCGCTCACATCCGAACCGGACGACGGCGGGC
TACCCGGATTGAAGCAGAAAACCTCCGTCCCCGGGCCGACGCTGGCGGATCGCTTCAGCG
GCGGCTGGGCGTCGGGATCAAACGCCGCGTTCAGATCCTCCTCCGACATCGACACGCACT
TCCGCTTCAGCGTCGAATTCCAGTGATTCTTAATCGCGTTGTCGGTCCGACCCGACAGCA
GCCGCGCTATGGTCGCCCACTTGTTCCCAAACTTGGCGTGCGCCCGGATGATGGTCTCAT
CCTCCTCCGGCGTAAAGGCCCGGTGCTCCACCTGAGGCGAGAGCTGGTTGCACCAGCGCA
GGCGGCAGGACTTGCCGGAGCGGCCGGGGATGGATTTGCAGATAAGCGACCAATTCCTGG
GGCCGTGCTTCTCCACCAATTTCTGGAGCAGTTGATCCTCCTCAGGGCTCCAGGGACCTT

TGATCCGCTCCATATTGGACATGGCAAAGGCCAAAGGGGACTGAATAATAACAAGAATAA
GGGTTTGGGGGAATTTATACAAAAGGGGGAGAAGGGATAGAGACGAAGCAGGGGTGGGAG
GCAGTTGGGAATAGCTGGCAAGGGACACGCGTCAGCCGGTGAGGGGGAAGGACGGACACT
AGG
>RMYB42
CATCACTTTTTAGAACAAGAATGCCATTTGGCATTGGTCACAATCCAAGTCAAAACCTAG
TCACAGTTTCTAAAGCTAATTTTGATTTTAAAACATACAATTGTCACATAACAAAAACAA
TGGAGATTACATAAGTGGGAGAAAAAATTATGAAGCAAAGAAATTGGTGTAAAAGAAAAC
AAGAGCATAAAGATGGTTGTTATTTATTTTGAATGAAAGAAAAAGGTCTATAATGCCAAA
GAGTGCATTTTTAGTGAATGCTGGGACATATAATTTCGCACTTCCTCCCTAATTATTTGT
TGTACCATGGTAGCTAACTCCGGGCTAAACAAGGAAGTTTTGGACGCCCTACCTTGCTCG
AAATTGGGAATGTTCGACAAATTTATTTCAAGGGGAGCTGAATTTCTAGCAAATCCCGGC
AAACTAAGACTTAATTCAGTCAAATCAACATTATCCTTAACCGGCGGAGTCGGAGGATCC
GCCGGTATATTCTTCTTATGATCACTATCTCTGGAATCAGAATCCATCAAACATGAACTC
TCCGATTCATAAATTATGGCAGCAGAGACATCTCTGTTATCCAATCGCCTCAAGGCTTGA
GGCAATCGGGCACTGCCGCCGTCGCTCCTCCCCGAGTACGACTTCCGTTTCAGCGTCGAA
TTCCAATGATTTTTTACGGCGTTATCCGTACGTCCGGTGAGCATCTTCGCAATCGTCGCC
CACTTATTCCCGTGCACCGCGTGCGCCTGAACAATGATCTCATCCTCCTCCTCCGTGAAC
GGCCGGTGCTCCACCTCCGGCGCCAGCTGATTGCACCACCGGAGCCTGCACGATTTGCCC
GATCTCCCCGGAATCGACTGGCTTATCAGAGTCCAATTCCTCGCGCCGTGCTGCTGCACA
AGTTTCGTCAGCAATTCATCCTCCTCCGGACTCCACGGCCCCTTCACCCGCTCGGGAACC
GCCTCCGGAGCCACCTTCTTGGCCTTCGCCGCCATGAAATACAATAACAAATTTGAAAAA
CCCTAATTATAACTGAATTTGTGTATATATATATATATATATATTGAATTTTGTTAAAAT
TGGTTAT
>RMYB43
GCAGCTATCAGTAGTCATAGGATTGCCTGTGTTGGAATTGGGGGAAGAATTAACAGCAGC
ATGTTGTTGTTGTTGATGTTGTTGCTCATGACTGGCGTCATTACATGAGTAAACATGATG
TCCACCTGGTCCTTGATTGGATACTTGTTCATGGTAATAAGCAGTGGTGCAGCCTTTTGA
ATTCTCCTTTCTTTTCTTGTTGCACTTGAGTAGATAATTATTGACACTATCCCACTTGTC
TTTGCACAGCACACCAGTCCTGTCATGTCCATAACAAGCCATTTTGGTTGCTATTTCCTC
CCACAGAACTTCCTCTGATGATACCCCTCTTTGCTGAAACTTTGCTTCCATTCCTGACCG
CAGCTGAATCAGCCTACTAATCTCACTCTCCGGCCAGATCATATCGCCTCCCTTTGCTGA
ATTAGTCATCGTTTCGCTCCCAATATCCTCATTGTTGTTCTGGATTTGATTCTCCGCCCC
TAGGAGATGATTATCCTTTCCAGTGAGCTTGTGAAGTGCTTCCATTAGGGCAGCGTCGCG
TGCTTCGATCCAGGCTCGTTCACTAGCCCAGAATTTTTGTTCCTTCTCCATTCTAACGAT
GTCTTGTCGCCTCCATTCCTCCTCTCTTACCATTCTTTCGTGCTCCTTTTGCTCCATTGT
CTTCATCATTTTCTCCATCCAGACCTCCTGTTTGTCCATCAATTTCTTCATTTGTAAATC
GATGAAATCCCGGATTTTAACTTTCCACCCCCTCTTCCCCCTCTTCTTGTTGCTCGGATC
ATCAGCCTTATCGTTGCCTTCGTTGAGATCAGTGTCGTCTGACGAACTACTATCCACTTC
GTCAGAATAATTCGAGAGACTAAGACTAGTGTCCGAGAGCTTCTGCCCTTGTAATGATTC
CTGATTGGCTCCTAAAGAAGTGCTCTTATTACCATAATTGTAGTGAAAATTACCTCCCAC
AAGATTGGCCTGATCCGAAGCCGAAGCTGCACCGTTGGAGTCACCATAAAGGGCTTCAAG
CTGCCTAAAGAACCTGTAATGTTTCCCATCTTGCCTCCCAGCTTTGCCCTCTTTAGTCTT
CTTGTAATATTTGTACAAATTTTCAAACTTCTCTCTGCACTTCTTACCGCTTCTCTGATA
ACCGTGCTCCTCAGACATTATCCTTGAGACTTCATCCCACAAGGGGCCCTTTTGGTTCGC
CTCTTTGAATTTGTGGTCAAGCCTCGATCTGATCTCAAGGAGAGTGAGCGTTTCCTGCCG
CGGCCATCTCCCGGCGCCGCCATCTCCACCTCCGATGTTTAAGCCGC
>RMYB44
TCTATGAGCTGAAATAGATGATGTTCCAAAATCATATTTATAATTTTAAAAAATTAATTA
CAACAAAAAAGAAATAGTAATTAACATCTAATTAATCCTAAAAATTCTAAGAATAACAAT
ACTGAAAATAATACTCCTATATAACCGATCCTTGAAAGCTCAACGGCCACCGGAAGCACA

TGGGCTCTGCCGCCGTCGATGGTTTACCCGCCCCCCGCAAGAACTCGGAGAAAGGCTTGG
ACTCAACAGACGAGGAGGAATTGAAAGAGCCACTTTTTGCATACTTCGTTTTCGACGGAG
AAGGCAGGCCCAACGACAGCTCCAAATCCACTGCGTACGGATCGTTTTCCACCTCCATCT
TCACCGGCGGCGGCGGTTGGGACTCCTCCGTTGTGGTGCTGCTGCATTTAGTATTATCCC
CAGAGGATGAATTATCAATTGCTGGACTGCTGCGAATATTGTTTGTGGCCTCATTCATTG
ACGTGGCTGCTTGTGGTGGAGTTGAGCGCCGAAAGTCCAGGCAGGCCTTAGCCGGAAGAG
CGGGGGCGCCGCCTGTGGAAGCGGCGGGGGCGGTGGAGTTGAAGGGGCGGTGAGTTTGAG
GGTCAATGCCACGGCTAATAAGCTTGCGTTTGATGTGAGTATTCCAATAATTTTTGATCT
CGTTATCTGTTCTTCCAGGCAATCTTCCAGCAATCAGGGACCATTTGTTTCCAAGAAGGC
TATGAAGCTTGATGATGATTTCATCTTCTTCTTCAGTGAAATTGCCCCTCTTGAGATCAG
GCCTGAGGTAATTTATCCACCTCAACCTGCAGCTCTTTCCACACCTAAGCAAACCAGCAG
CTTTAGGAAGGGAACGCCAGCAGCCTTCACCATGGGCGCGGATGTAATTGATGAGGCGCT
GGTCTTCTTCCTTGGTCCAAGCACCTTTGTTGGTGTGGGCTTTTTCACAGCAAGGGGACC
TTCCCATGTTCAAATCAAAATATGGACTTTGAACAAATCAAAAAGTTGCTCTTTTTTCCG
TCTATCTGTAGCTGTATCTGATGA
>RMYB45
ACGGCACAAACTAACAAGGTCTCCTAAAGAACATTGTATGTAAGTTTGTAAGAAACTAGA
GGTAACAATTGGCGAATGGACATTTGGAACAGAAGGGGGAAAATCAACAATCCATCATAC
AGCTAAGCCGGGAAACTTTGCAAGTCATCTGATGCAGTTACAGCTAATTAATACACATTT
TCGATATCATAAGCTTTTTTGGCAAAACCACACATATCATTATACTATTTGACGAACTTA
ACTAATGCAGATACACAGATGGGTTCAGAAAATCGTTTCAATCAAGCAATTTAAAGGATC
CAACACAAAGTACATCACATTACATATTGACTCCTCAAGAGGCGATTAAATTCGGAAATG
TGGATGAGACGCACCGGTCTTGACATTATGCCAAGTTTGTGCAGCAACATTGCAGGGATA
TGCAACGAGCAAGTCACCATGAGCTGAGTCAAGATCCATTAAGGTTTTGTCGTTGATGTG
ACTCCGGTCCACGAATACTTTTGGCGCGTATGTGGGCTTTTGCACAGATGTATGCTGAAC
ACTAGGTTTCTCGAGAAGGGGGAGGTTTGTTGTTTTCTGAGGAGGAAGTGTTGGTGAGATG
TATGGTCGTTATGTCATGGATGCTTGGCCTCCGTTTGTCCTTGGCTCCGGAAAGCTGCCT
TAGATAGTACTTTTGCGCATGGCTCGCTACTTGTGTGGGCGTTTTGGATATTACAAAGTT
TCGTGAGATATTCCTCCAGTCCCCTTTTCCGTGCTTTTGAAGCCCCAGGAGAAATCGCCT
ATGCTCATCCTCAGTCCACGGCACGCCTTTCTTGCGTTCTTGATCGCAGGTTCGGACCCT
CTTTCGACACACATCAAAACTGCGAAGATCCGCCAATTCATAGGTAAAGGAAGAAGCCAA
GTATGCAGGAATGGGGAATAAACCTGCCTCTATGTCAGAAACATCTGCCACTAATTCCCT
ATACTGATTTATTACATCATACACAGATTTCCCTGGGATCATTGCTGCAACCTTAAACCA
TCTGTTGGGTGTTTTCTCGTCGAAAATCGCCAGGGCACTCTCGAACTGCTTGTTTTCCTC
TTTTGTCCAAACTGTGGTCATCCCCTGGTGTATGAACCAGTTTGAATCTGATGCCAGGGA
TGTTTGGCACAAGGTCTCCATTTCATCCAAAATCAATCCTTTTTTGAGGGGTGAAACTGA
AGTGCACCTCAAATTTTGCTCGGCCCAGTTGAGAATTGGTGAATAAGGAAAAACCCTGGT
GGCAAAATTCCTGAAAAAGATTATGAGGCAAAGGAAAGGGGTGAAAAAAGGAGTTTTATA
GAAGCCATGAAAAAGGCAGTAGCAATAATCTGAGTGTGAGTTTATGATCAAGACTGTGGA
GAT
>RMYB46
TGTTCGAATGACAACTGTCCCATAAAAAATTATGCTTTTGCGGCATCCACATAAAGATTT
TACTGCATTGTATAGATTATAGATAATAAAGGCTAGAACAATGGTAAAGAAAGCTATGGC
CATTAGCCACATAATTAGTTATTTTACAACTATCTAGTTTCTCCATAAGGGTCTATCCTT
GAGACTCAGTAGCCTCACTTTACCACAAATTCTAGAAACGCTTCTACAACACCACATATG
CTGTCTTCACAGATTAAGGATGCAGTTTTATCCTATCCTATAGCTTATTTCATATTTATT
ACGAAAAAACAAGAACATGTGCGCATTCATCCGAAGCCATTTCATGAACATTCGACGGCA
CTATTGCCACATGGGTTCATCAACATCTGACATCATTATCACAACTTAGGCAGCAGAAAC
ATACCTGTCAAGGTCAAGTAAGCACAAATCTTTATTGTTTACTTGTGTGGCACTGGGTAA
GTAATGGGAGCCATGGTCAATGGGGTGCCGGGAACCACGGGAGGTCCAGGAATTGGGGCT
CCAACGCTATAGGCCAAGTGGGGAGGTGGAAGGTTCACCGGTGTCCCAACTGCTGAAACG

AGTGGGCCTCCTATTGGTTGTCCAATGGTCGGTGGACCGTACATGCCAATGCCTGGGGTG
CCAACAGCAGTTTGAGGGAGTGGTTTGTTGGATTTCATGGGTGGGCCTCCTGCAGCAGCA
CTATTTGTCTGGCCAGTGATGGGAGTTTGGGGCACCGAAACATCTCCATTGTTGACGCTG
GTGATGTCGTGAATGCTCGATCGTCTCCTGTCCTTGTTGATAGAGTTCAACCGAATGAAG
TACTTCTGGGCGTGGCTGGCAACTTGGGTAGGAGTCCTTGTCACCACGTAGTTTCGGGAT
ATGCTTCTCCAGTCGCCTTTCCCGTACTTGTCCAAGCCAAGAAGAAACAATCTGTGCTCA
TCTTCTGTCCAAGCAATGCCTTTGCGACGCTCTTGATCTGACTTAGAAGACTTGCCTCCA
TGGCTGGAATCACCGTTCAGGCGTCCAGAATTGCTACTTTTCTTGCCACTGGCTTCATCA
CCACCGTTACTAGTTGAACCATCTGAGGAAGAACTGTAACAGGGCACCTCTACCACACCA
GATTCTATTCGGTTGACGTCATCCACTAAAAGTTCATAGTGGTGTTTAACCTCTACAACA
GATTTGCCAGGAACATCAGCTGCAATTTTTTCCCATCTGTCTAATCCATCCTCTGGATAA
GTGGCTATTGCTTTCTCAAACTGTTTATCCTGCTCCCTACTCCACACAGAGCTGCTCGAT
TCATCCGATGGCATCTATACTTGTTTAATTTAATACCAGGTGTAC
>RMYB47
GGACGGCAGCTTCTCCATCAGCCTGGCCTTCTTCCTCAGATGCGCATTCCAGTAGTTCTT
GATCTCGTTGTCGGTTCTCCCCGGAAGCCTCCGGGCTATCAACGACCACCTGTTTCCCAG
AAGCCTGTGAAGCCTGAGTATCAAATCATCCTCCGCTTCGGAGATGTTGCCTCTTTTTAT
GTCTGGCCTCAGATAATTCAACCATCTCAGTCTACAACTCTTCCCACACCTATTCAACCC
TGACTTCATGGCAACGGATTTCCACCTCCGGGGGCCGTGAATCTCTATGAATTTGGCGAG
TGTGGCGTCTTCTTCGGGAGTCCAAGCTCCCTTGTTAAGTGAAGATGATGATGATGGTGA
TGATCTCTTTCTGGAAAGAGCCCCATCGCCGTCGTCGTCGTCGTCGTTATCATCATCATC
GCCGCCATTACCATCGTGATCATGAGGAAGATGGTGGCGATTCTTGTCAGCCATGGGATT
GGGATTGGGTGGGCATGATGGAAATGGATAAGTTTAAGTTAAAAGGGGAAGGGATTGGAT
CACTGGATGGTAGTAGCCGTGGGGGTTGAAACTTTGAAAGGAATATAACCGCCGGGTTAG
TGAAGCCCGACTGCCCCAGCTGGCCAAATCTCCCAGAACACGACAGGTAATTGTAGCG
>RMYB48
GATGCACATTTATAATTTAATAAGTAAAACCTAGTGAAATAAAACAAAACCCATGTTTTT
TACATATACACTCCGATTCATACGTAAACAAAACCAAGCAGAAGTAAAGAACCAAGAAAA
GAAGAACAAATAAAGTTCTCATTACAATCACTATACCCCCAAATTAATCCCAATTTATTC
CCTTGGATCTAACGCCCCTTAACATTATTCACCCCAATCCCTCATTTTATTCCCACCCAA
ATCAAATAAATAAATCAAAAATTTCAAACCACATATAAAAACTTCCCCTATGACCTTACA
AAATCTTTCAAATTTTCACTTGCTTCCCAGTTGCTTATTTTGTCCTGCCCTATTCTCATT
CCATCCTTCAAATATTCGAGTATGCTTTAACACGCTCTTTCCTCTATCCGCTCAAACAAG
TTTTACCTGTTCAATTCGGACCACAACAAGCCAACAGCAATGTGCAAACACCTCACCCCA
TCGTGGATGATTGTTTGTTTGCAATTAGCTCGTACTCCCCTCCATTCTCGTCCATCTCAT
CCTCCTCCTCATCAAATTCATCTTCCTCATGCTCGTTGTTCTCGCTCTCATTCTCATTGT
GATTCTGATCGCGAAATGTAGAATCCTTTTGTTGTTGTTGTTGTTGATTGTTGTTAGGAA
GAGGCCATTGCTGCTCGGGCCGGACTAGGATGGGTTCCATATTCGGGTTGTTTGGATATG
AGGGGTTCATCGATGAGACAATGCCATGCTTTGCCCTCTCTTTGTATATGGCTTCCAACT
GGTAGAAGTATGGGCATGTCTTGGAATCTTCGGGCCTCTTCTTATTACTCTCCTTTACTT
TCTTATAGTACTTATTGATGTTCTCCCATTTCTCCTTGCACCTCTTGGCGTTCCGGTTGT
ACCCGAGCTTCGCCATGGCAGCTGATACCTCCTCCCATAATGGACCCTTGGGCGCGTTAT
CTTGGTACTTGAAATCCAAGCTTGTGCGGATGTCAATCAGGGCCTGGATTTCGGCTTTCG
GCCACCTGGAGGAACTCCCCGGCAAGACACTCTCCCCACCGCCGCCATTGTCCGTTTTTG
AAGTCTCTACAGCTTTTGTTGTAGGAGGATTCGATTGCTGAGGTTGTTGCGGTGGCGGTG
GAGGAGGAGGTTTTGCAGGCGCTGGCGTAACCGGCGGGATTTGGGGATTATTTGGTCGG
TGACCTTCTGCAAGAATGCTATCAATGCAGCATCCTTAGCAGCGGCCATGGATCTCTCCC
GAACTAGGATTTCGTGCTCCCGATTCATCCTGGCCGTCTCCTGCACCCGCCAGGCTTCCT
CCCTGGCCGTCCGATCCTTCTCTCTATTCTCCAGAGTTTCTAGAAACTTGATCTGCAGCG
CCTCCTGCTTCTCCACAACCTCCTTCATTAACCTCTCGAAATAATCCTTCCATTTCCTTT
TCTTCCCCCGCCGCCGCTGGATATCCTCATCGGAAGACGTCGACGACGACGAAGTCGTCG

ATTCCGATCGGAAGATGCGAGCTTGCGGCGTAATCTGAGAATTTGTTGCCGCAATTGTGT
GCTGGTGCTGGATGAATTGCGGCGGCTGAGATTGCTGAAATTGAGGAGAATTCAATTGGG
GATTTGTGATTGGGTGAATTACACTCAAAGGGGTTGGACTGATTGATGAAACAGTAACAT
GTGATGGCATTTGTAGATTAGGAGGAGGGGCCGGAGCCGCTGGAGCCGCCGAGCGCGGCG
GCGCCGGGGAGTTTTCCAGGGCTTCCAATTGCTCAAAAAACCGGTAGCTTTTGCCGTCGG
ATTTGGCGGCCCGGCCGTCCTTTGTACGCTTGTGGTATTTGTACACGTTCTCGAACTTCT
CCTTGCATTTTTTGGAGCTTCTTTGGAATCCAAGACTCGCCATTTTCCTGGAAACTTCTT
CCCAGAATGGGCCTTTA
>RMYB49
AATATGGCTAAAGACATCAGCAAATCGCCCTCAGAATAAACGTTATTCAAACCATCAACT
AAGTTGAAAAAATCGCGAGTTTTCTTGCAAACCAAAACCTAAGTAAAAAGCATCAATGAG
AAGACAGGGCACCATCCAAGTTAATCTGATCGACAGATAGGTAGAACAGAATTACACTGT
CGATGCTCAAACTATTGTTTTTGTCATCGATACAATATGCTCAAACTATACGTGTAAATA
GCAAAATGCTACTAAAAACTATACCCTTATTGATACATATGTATGAATTACAAGGATATT
TCAGACAATGGCAGTTCATTTTTTTGGGTTTTTTTGGCAATTGGTGACTAATGATAATGA
TATCTACATACTATGGATGAAGCATTCTTATGAAATGCCTTCCTTGGAGTGGAAGTACGT
TCGATTCTTCTACCTGGGAAGCATAATGTTCTTGGATGATGCTACTTCTGATAGCACGCG
ATTGATTTCCGTTGTTTCCAGAAACCTGTCATGAAAAAAAATGGTATTGAAGTACACAAT
CTTCATCTGCAAAACGTTATCGATGATCAATCAAAAAGGCCCCAACAAACTGTAGAAGTT
TTAAGGATTTTGTGATTCCAATTACAGAATAACTAATAGTTTCTGCAGCTAACTTTACAG
CTCTTTTCTTAACCATAGCCACAGAATGGAGGGGTTGGCGGATTCAACTATGCCGAGTGG
GATGCATCTGGAAAATCGTACCAGGATGTCCTAACCAGAGGCCACGCTGGTCGAACCCCT
GCATATGATGCTCATTCAGGTTAACTCCAAAAGAAGAAGTTCCTTGAAGGCCAGTGATCA
TTAAGCCGCCGTCCTGTGGGGTGAAGCCCATCATTGCACCCGGGTTGGTCAGGTTGGAAT
CATACGTTGCCCTGACCACAACATTACCACCCAAATTGGGGTGAGACTGCAGAATCATAT
TCGATTTCTGTGGCGAATGCGAGGCGTCCTTATCCTCCGGTGAAGGAGACTTGTTAGTTT
CATCAAGATTGACAGTGGTGATATCATGTATGCTGGACCTCCTCTTATCCTTCCCGCCCG
AAAGCTGCCGGATAAAATATTTCTGAGCATGACTGGCCACCTGAGTCGGCGTCCTCGTGG
TCACGAAATTGCGGGAGATGTTTCGCCAGTCCCCTTTTCCGTACTTCTTCAGTCCCAACA
GAAATTGTCTGTGTTCTTCCTCAGTCCAGGGCACTCCCTTCTTCCTCTCATGGTCCGAAC
ACCGCGTCGACGAGCCGCGTTTGCCGCCTGGACAGTAAATTTGTTTCAAACCATCATAGC
CATGATTGGTCACCCACTCCAAAGTGAAGGAATTATTGGAGTAGCCCGGAATTGGGATCA
GCCCTGCTTCGATATCGCTCACATCTTCGACCAATTCCCTGTACTGTTTCATCACATCGT
TGACAGTCTTCCCCGGAATCATCGAAGCCACATTCTGCCACCGGTCGGGAGTGTCCTTAT
CAAATAATGCCAGAGCATTTTCGAACCTCTTATTCTCCTCAGGAGTCCATTTCGCGACAG
TACTCCCTTCGAACAACCAATTCGAATTGGTGAAGTATGAGGCAGGAGAGACCTGCCCGA
TTTCCCTATTCATCGCCCAAAAGTTTGGGGGGCAAAAAATAACACCTGAGGAGGCAGAAA
TATAGGGAGAAAGAAGCAGAGAAGAGATTTGATATATGAATATAATCAGATGAGCATTTT
AAGTCATTTTGGGGGAATTGAAGAATCTGAATACTTTTTGACACATGGTGAGGAATCTCA
GAAAGGCTCCCCAACTTTCCTAACCCCAAGGAAAATTCAATTCTTCAAGTATTCAACCAA
CTGAAGAAACAAGGAAAAAGGGCAGATCTTGCTGCAGAGAAATAACAAACCCCCAAAATT
CTATTACCTCCTCAGTCCTCAACTCACCACCCAACTCAAATAAAGAAAAACCCCTTCAAA
TTAAACATTACAAGAAGCATCGGGAAAGGGCTTAAAAAGACTACAATGATAGAAACCCAG
ACCTCTGTCTCTGTTCGAATAATTCATACCTGAACCAGAAAACAGAGTAAATAACGCAAT
ACAACAAGGCCCTCAATTCTGAATGGCTCCAACAAATCAAAGGTGTTTCTGAATGATTGG
TTGGGAGGTGTCTGTGGGGGCTTCAGCTCAGGGCTT
>RMYB50
GCGGGAAGAGCTGCCGCCTCCGCTGGATCAATTACCTCCGCCCGGACCTCAAACGCGGCA
ACTTCACGGAGGAGGAAGATGAGGTCATTATCAAACTCCACAGCCTCCTTGGAAACAAGT
GGTCATTGATTGCCGGGAGGCTGCCGGGGAGGACGGATAATGAAATCAAGAACTATTGGA
ACACGCACATCAGGAGGAAATTGTTGAGCCGGGGAATCGATCCGACGACTCACCGGCCGA

TGAATGAGGCGGCGGCGGACAGGGAAACCCTAAAAACAACAACGACGACGACTATTTCGT
TCTCCGGTGGATGTTCGAGAGAATCGGAAGATCAGAAAATCATTGTGATCAGAAAAGATG
AGGATCGACGGGG
>RMYB51
GAGGGGAAGGATTAATATTGCTGTTGTTTTCAAGCTTTTTCTTGAGATGGGTATGCCAAA
TGTTTTTGATTTCATTGTCTGTTCTTCCTGGCACTCTTGCTGCAATGGCAGACCACCTGT
TTCCAAGCAATTGATGCAAAGTGAAAATGGTCTCTTCTTCCTCTTTGGTGAAATTTCCCC
TTTTGATATCAGGCCTCAAATAATTAGTCCACCTCAGCCTGCAACTCTTCCCACACCTCA
ACAAACCTGCTTGTTTGGGAAGAGCTCTCCAATTGCCATGGCCATGTTGCTGAATATAAG
TGACCAAAATCTGGTCTTCTTCAGGGCTCCATGGCCCTTTCTTCAGCCCCATTTTTTCAC
AGCAAGGTGCCCTCACCATTCTCCTTACTTAATTATGATAATTTGGTATAATATTTCTTA
TTTCTCTATATGGGTTTTAGAGTGAGAAAAACTGTAAGGAAGAGGAGAGAGAGAGAGGTG
GTAAGGTGTA
>RMYB52
TTCTCGTTTTGAGTTGATGACTCAAGTGGATCGCTCAAGTTGGAGTTGGAGTTGGAGTCG
GAGTTTGAGTTTGAGGGGGTTTCTCCGCCGTGCTGCTGCTTGTATTCCTGTAGCTCTCT
TTCATCAGTCCAACTTTCTTCTTACACTGGCTCACAGTTTTTCCTGGAACTGCAGCTGCA
ACTCGTTCCCAGCGTTGAGGAGTTTCCTTTGGAAAGGTTTTCAGAGCTTGGACTAAACCT
TTTTCTTGAGCAACAGACCATGAGTCTTGATCTGAACACAAAGCAGTTCCATTAACAGCA
TTGCTGCTGTCTGACAAATTGCCAGGAACAGAAGATTGCCCCATAGCTGCACCGGTATTA
GATAATCCCAGCCCATCCTCTCTAGTTGAGAGAGGTGAAGCAATTGCTTGTGCAGGTTTC
CTTTTCTCAAGGAAGGAGTCAAAGGCTTTAGCAGAATCAGGCTTTTGCAAAAGAACTGTT
TTTGTAGCCTTCAAAATCTCCTCTACTGATCTCCCAGAGCCAATATACTCAGATATCACT
TCCCACCGCCGGGAAGTTCCTTTAGGATATTTCTGCATGCCCTTTCTCAAAAGCTCAATT
TCTTCTTTACCCCAAGGCTTTTCAGCTTTGTTAGAAACCTGTCCATTCATATGAATGCTG
GTGGAGCCATTTTGCAGTACATTCTTCTCATCTTCCTTCTTATCCTTTGTTTTGTGATTG
GATTCAAGTGTCTCTTTGAGAAGTTCAGCTCTCTCGAGACCCTCCTTTCTTTCCAATTTT
TCACACAACTTACTAAGCTGGTCTTTATCAAGTGATGAGCACAGACTCTCCACATCGTCA
TCAGTGAGATCAAGCAAACGTTGAGACAAAATAGGCGTTGAAAGTGTTCTTAGACGAGTC
CGTTCCTTCCGGAGTAATTTTTTCTCTTTTTCCTTCAATTTCTTTTGATTTAAAGCAGCT
TCAGCTGCTCTTTTTTCCTCCTCTTCTTTCCGCTTCCTTTCCTCCTCGGCAATCCTAGCA
GTTTCTTCTTCCTGCAATCTTTTGGCCATAAGTTTAGCTTCCTTCTTTCTTTGTTTCTCA
GCTTTCTCCTCCTCTTTCCTTCTTAAAATCCTAGGATCCCTTTTATAGGCATTATCAACA
AGAGTACGTATGCGTGCACTTTCATCTTTCCGTGCCTTCTCTGACAGTTTTAAATTTTGC
CTTTCCATCCATCTCCTGTGATCTCGACCTTCAGCTTGCTCAAGGTCATACTCATCAGCA
TGTGGAAATTCTCTCCAACTTTTGAAGGAATACCAGAAATTATAAAAGCTATCCACTTCT
TTGATGGGCGTTTTATCATCCCCCAAAGTAGGAATAGGCTGGGTTACAGACCACCGCCCG
TTCCTCACAAATGCAGGGCCAAATACCTTGAAGAAATCCTGTGGTGCACAATCACAAGGA
ATTTCATCATCAAACTCATCCGTGGAATCATAGATTCTTCTCTTCAAAGGATCAATCAAC
ACTTCATATGCTTCTTGAATTGCTTTAAAATGGCCCTCAATCTCATCCTTCTTGGCCTGC
TTAGCAGTCTCAGTTTCCTCCGCAAGCAAAAGAGCAGCCTGCTTGTCAGGATGATGCCTC
AGAGCAGCCTCCCGGTAACTCTTTCTTATCTGATCTTCTGTTGCAAGATACCTCAGATGG
CTCAATCCTAGCAGGGCATAATGGTCTTGTTGTTTCGCTTCCCCAGACTTCTTCTTTCCC
TTATTTCCATACGAATCTGATGATTCTACATAAACATGTTCCTTCTCATTGGGAGCACCC
TTTCCATCATCATCACCACATGTATCTGCTTCCACACAGTGGCCTACCAACCTAAGAGCA
GCAGCATTAAACGAATGCCCAGCAGGCTCAAGTTTCAACGCCTTGACCGGAAGGCTATTG
GAGGATACATAGATTGGTTTACCATCCACAATTTCCTCGTGGTAAGTAATCAGCTGTATG
CTTTTCCCAGAGGCCATAGCAACTAACAAGCCAACTAGGACAGGATCCCAGAATCCAGCA
AGCTGCCGATTAATGCGGAGAGTGGAGAATTTGATCGATATTAGTTTGGCATGAATCAAT
CGCGATTCCGCCTCCGCCGACGATGATGGTGAAGGATGGATTAGACGATGATGGTGAAGG
ATGGATTAGAGCTAGGGTTTTGGTTTAGGTTTGGGTGAACGGCTTGTGCAAAGGAAGAGA

GAAAAAAACAGGGATTAAGGAAGGCGTCCGGCAATTTACCC
>RMYB53
TTTTGGGATTCTCATTTTGAGTTGATGACTCAAGTAGATCACTCAAGTTGGAGTTGGAGT
TGGAGTCGGAGTTTGAGTTTGAGTTTGAGTTTGAGGGGGTTTTCTCCACCGTGCTGCTGC
TTGTATTCCTGTAGCTCTCTTTCATCAGTGCAACTTTCTTCTTACACTGGCTCACAGTTT
TTCCTGGAACTGCAGCTGCAACTCGTTCCCAGCGTTGAGGAGTTTCCTTTGGGAAGGTTT
TCAGAGCTTGGACTAAACCTTTTTCTTGAGCAACAGACCATGAGTCTTGATCTGAACACA
AAGCAGTTCCATTAACAGCATTGCTGCTGTCTGACAAATTGCCAGGAACAGAAGATTGCC
CCATAGCTGCACTGGCATTAGATAAGCCCAGCCCATCCTCTCTAGTTGAGAGAGGTGAAG
CAATCGCTTGTGCAGGTTTCCTTTTCTCAAGGAAGGAGTCAAAGGCTTTAGCAGAATCAG
GCTTTTGCAAAAGAACTGTTTTTGTAGCCTTCAAAATCTCCTCTACTGATCTCCCAGAGC
CAATATACTCAGATATCACTTCCCACCGTCGGGAAGTTCCTTTAGGATATTTCTGCATGC
CCTTTCTCAAAAGCTCAATTTCTTCTTTACCCCAAGGCTTTTCAGCTTTGTTAGAAACCT
GTCCATTCATATGAATGCCGGTGGAGCCATTTTGCGGTACATTCTTCTCATCTTCCTTCT
TATCCTTTGTTTTGTGATTGGATTCAAGTGTCTCTTTGAGAAGTTCAGCTCTCTCGAGAC
CCTCCTTTCTTTCCAATTTTTCACACAACTTACTAAGCTGGTCTTTGTCAAGGGATGAGC
ACAGACTCTCCACATCGTCATCAGTGAGATTAAGCAAATGTTGAGACAAAATAGGCGTTG
AAAGTGTTCTTAGACGGGTCCGTTCCTTCCGGAGTAATTTTTTCTCTTTTTCCTTCAATT
TCTTTTGATTTAAAGCAGCTTCAGCTGCTCTTTTTTCCTCCTCTTCTTTCCGCTTCCTTT
CCTCCTCAGCCATCCTAGCAGTAGCTTCTTCCTGCAATCTTTTGGCCATAAGTTTAGCTT
CCTTCTTTCTTTGTTTCTCAGCTTTCTCCTCCTCTTTTCTTCTTAAAATCCTAGGATCCC
TTTTATAGGCATTATCAACAAGAGTACGTATGCGTGCACTTTCATCTTTCCGTGCCTTCT
CTGACAGTTTTAAATTTTGCCTTTCCATCCATCTCCTGTGATCTCGACCTTCAGCTTGCT
CAAGGTCATACTCATCAGCATGTGGAAATTCTCTCCAACTTTTGAAGGAATACCAGAAAT
TATAAAAGCTATCCACTTCTTTGATGGGCGTTTTCTCATCTCCCAAAGTAGGAATAGGCT
GGGTTACAGACCACCGCCCATTCCTCACAAATGCAGGGCCAAATACCTTGAAGAAATCCT
GTGGTGCACAATCATAAGGAATTTCATCATCAAACTCATCCGTGGAATCATAGATTCTTC
TCTTCAAAGGATCAATCAACACTTCATATGCTTCTTGAATTGCTTTAAAATGGCCCTCAA
TCTCATCCTTCTTGGCCTGCTTAGCAGTCTCAGTTTCCTCCGCAAGCAAAAGAGCAGCCT
GCTTGTCAGGATGATGCCTCAGAGCAGCCTCTCGGTAACTCTTTCTTATCTGATCTTCTG
TTGCAAGATACCTCAGATGGCTCAATCCTAGCAGGGCATAATGGTCTTGTTGCTTCGCTT
CCCCAGATTTCTTCTTTCCCTTACTTCCATACGAATCTGATGATTCTACATAAACATGTT
CCTTCTCATTAGGAGCACCCTTTCCATCATCATCATCGCCTGTATCTGCTTCCACACAGT
GGCCTACCAACCTAAGAGCAGCAGCATTAAACGAATGCCCAGCAGGCTCCAGGTTTAACG
CCTTGATCGGAAGGCTATTAGAGGATACATAGATTGGTTCACCATCCACAATTTCCTCGT
GGTAAGTAATCAGCTGTATGCTTTTCCCAGAGGCCATAGCAACTAATAAGCCAACTAGGA
CAGGATCCCAGAATCCAGCAAGCTTCCGATTAATGCGGAGAGTGGAGAATCTGATCGATA
TTAGTTTGGCATGAATCAATCGCGATTCCGCCTCCGCCGACGATGATGGTGATGGAGAAG
GATGGATTAGAGCTAGGGTTTTGGTTTAGGTTTGGCTGAACGGCTTGTGCACAGGGCGAG
AGAAAGACAGGGATTAAGGAAGGCGTCCAGCAATTTACCAAAGGGCC
>RMYB54
CCCGTTGTGAAATGGAATGAACCAGGATGTAGTATTGCATCGTCTTGCCAGAATGGATCA
AGGTATGTTGGCCTTCCTTTTGCTGAAATGCCCTGTTTGGAGATCAACTGTGTTGAGAAT
GGAGTATCAGGGGTAATACCAAGAGGTTTAGGAGACAGTGGATGTTTGCCAGCACTAATT
GGGTTCCCAGGCATGATACCGGTAGGAAAAGATGGCATGCTACCAAGATGCGCAGTTGGT
GTATCTTTGATGTTGGACTGTTTTTGGTTTCTCCTAGCGGTACTCAATCCAGTGAAGTAT
TTCTGAGCATGGCTAGCCACTTGCGTATTTGTTTTAGTCGGTACACAATAGCTTGAAATG
CTCTTCCAGTCGCCTTTTCCAAACATCTCCAGGCCGCATAAAAAAGACCTATGCTCCTCT
TCTGTCCAACGAAATCTCTTCTGCTTATGAATTCCCAGTGGTTTCTCTTTCTTAGCTTTA
TTCTTACTAAAATGATGAGGCAATGGTACCTTTCGAGACTTGACTATTAAGGCTTCATAG
TGAAGTTTTAACTCTTCAATGGATTTTCCAGGAACTGCCAACAAAACCTTATCCCATAGA

TCAGGATCACCATGGAAGAATGCCAAAGCATTGTCAAATGCCTCATCCTCTTCCTCAGTC
CAATTCCAACCACCATGTTCTTCATCCTTCCAATGTATTCCCTGTTTCCCACCAATTCTC
AATGGCATCCCTTTCTTTTCGCAAACCATAATATTGCTTGGATAATGAGGCAAAGGCACC
TTACCTGACCCAATTGCCTCCACATCATATCTTAAGGCTTTATAGTGATGTTTTACATCT
TCAATTGTCTTTCCAGGAACTACAACTGTGAAATTCTTCCATGGATCAGCATCATTGTGG
CATAGTGCCACAGCATTCTCAAATGCCTTATCCCTCCCCCAAGTCTGAGATGAACCACTG
CAGGCGTTCTTAAATGATGCTTCCACCTCCAATGCCCTGGAGCAGCTGCTGGATGACCCA
CTGGTGTCCATTCTTCAGGTAACTGGTTAGGGGAGACTTATTGCTCAGATTAATGGCAAG
AAACTAGTCTGGTTGGCAAATACTTTCAAGAAAAAGGTGCTTGTCTTCACAACCTCATAG
AAAATTCTTCTCTATGCTGATTTTCTTCTTAGTCGGTAATTTATTTAGAAGAACCAAACT
GTGGGTGAGCGTTGTGTGTAACTTTGTCCTTTGTCAGCAACAGATCAGACTTGCATACAA
GTTTGGTGAATCTGGCGCATATAAGTGATTCCAACAGTTCTTTGCTGTGGGCATAGATAA
TGGACAAAGCAACTTTCTACCCGGAACAAGCTGACATATAATATTGCTCAGCAGCCACAT
CGAAGTCTGACTACATTCGCTCTTGTAACTCTGTAAATGAAAGGTAGATGACCCATAATA
TACACAGATATATACATATAATATGTCTACACTTGGGTGTGTGTGCGCGTGTCTCATCAC
ACATTGAGTATAATGAGCACCATAATGTCTTGTAAGTTTGTTAGTGAAGAGGGTGCCCGT
GCAGTTGATTTGGTTCACCAACTAGTATCACCATATGAATTAATTAAAATGAGAATCCCG
AATAAATGCCATGCGCCGTTGTTGGCAAGC
>RMYB55
CATGAAGAAAGGAAGAACCCTAAATTGAAATTGGGAATTTGATAAAACGGGAAGGATGAA
GAAAACGGCCTTTTGAATAATACCGTTAGAGTGAAAACTAGAAAAGTTGCTGAATAAACT
TACAAAGTCGTGTTACACTTATATAATAAAATAGAAGGCACAAATTACAGAAATGCCACT
GACTGAATATTACAAAACAGCCCCTGCATGCTTTAACCAATTCCTGCAAGGTCTTCATTC
TCTTCTCTTGCTGTATAACTGTATCATGACAATTAGATTTTGTCCTTTTCCTACACCGAC
TAGTTATTTAGATTAGATGTAAATAAGAAAGTAAAGCAGGCTTTACCAGCCCCTTTATAA
TCCCATTACATTATTGTCCAAATCTATTGACCCCACACACACACTTAGTATATAAACTGC
AGTGATCAACAGCAAATCAAGGTTCATTTCTCGACCCCCTCCCAGCTGCAGCTTCTTTCC
CAGATGGAGCTGCAAAAGCAATTGAGAGGTCGAGGTTCACCTGAGGAGGCGGCAACGACG
ACGTGGTGGAAGTCTCAGAGACAGCTTTCAAGCAGGTCTTGGACGACGACAACATTTTGT
TGTCCGATGACGGGTTGGCTGTGTCAACGTTGGAACAGGCAGCTGCAGGTCGGGTGGTGG
CAAACATAGTTTGGTGGATGCGGTGGTTATTGGGATCGATCCCCATTGCAATAAGCTCTC
TCTTGAGGTGGGAGTTCCAGTAGTTCTTGATCTCGTTGTCTGTCCGCCCGGGCAACCGCC
TGGCTATTAGAGACCACCGATTGCCAAGGAGGGCATGGAGCTTGATGATGAGGTCGTCTT
CATCGTGGCCAAAGTTGCCGCGTTTGATGTCTGGTCTTAGGTAGTTCACCCATCGCAGCC
TGCAACTCTTCCCGCTTCGCTGCAACCCTGCAGCGTTGGGTAGGGAGGTCCAGCAGCCTT
CACCGTGAAGTTTGATATAATCAATAAGCATCTGATCTTCCTCTTTAGTCCAGGCCTCTC
TGCCTCCGCCTCCTCCTCTTCCTCCTCCGCTGCTTTCTTCCTTCTTCTTACCGCTAGATT
TCCTCATCCTTGACCTCGTACACAATTGCAAGAAGGGAAATCCACCTTATATATACCTAA
AAAACCAAATTCGGACAAGTAGCTTCTAAGCAAGTCTGGTTGATGGGAAGAAGATATATA
TATATATATATATATATATATATA
>RMYB56
GATACGTAAACAAAACCAAGCAGAAGTAAAGAACCAAGAAAAGAAGAACAAATAAAGTTC
TCATTACAATCACTATACCCCCAAATTAATCCCAATTTATTCCCTTGGATCTAACGCCCC
TTAACATTATTCACCCCAATCCCTCATTTTATTCCCACCCAAATCAAATAAATAAATCAA
AAATTTCAAACCACATATAAAAACTTCCCCTATGACCTTACAAAATCTTTCAAATTTTCA
CTTGCTTCCCAGTTGCTTATTTTGTCCTGCCCTATTCTCATTCCATCCTTCAAATATTCG
AGTATGCTTTAACACGCTCTTTCCTCTATCCGCTCAAACAAGTTTTACCTGTTCAATTCG
GACCGCAACAAGCCAACAGCAATGTGCAAACACCTCACCCCATCGTGGATGATTGTTTGT
TTGCAATTAGCTCGTACTCCCCTCCATTCTCGTCCATCTCATCCTCCTCCTCATCAAATT
CATCTTCCTCATGCTCGTTGTTCTCGCTCTCATTCTCATTGTGATTCTGATCGCGAAATG
TAGAATCCTTTTGTTGTTGTTGTTGTTGATTGTTGTTAGGAAGAGGCCATTGCTGCTCGG

GCCGGACTAGGATGGGGCCCATATTCGGGTTGTTTGGATATGAGGGGTTCATCGATGAGA
CAATGCCATGCTTTGCCCTCTCTTTGTATATGGCTTCCAACTGGTAGAAGTATGGGCATG
TCTTGGAATCTTCGGGCCTCTTCTTATTACTCTCCTTTACTTTCTTATAGTACTTATTGA
TGTTCTCCCATTTCTCCTTGCACCTCTTGGCGTTCCGGTTGTACCCGAGCTTCGCCATGG
CAGCTGATACCTCCTCCCATAATGGACCCTTGGGCGCGTTATCTTGGTACTTGAAATCCA
AGCTTGTGCGGATGTCAATCAGGGCCTGGATTTCGGCTTTCGGCCACCTGGAGGAACTCC
CCGGCAAGACACTATCCCCACCGCCGCCATTGTCCGTTTTTGAAGTCTCTACAGCTTTTG
TTGTAGGAGGATTCGATTGCTGAGGTTGTTGCGGTGGCGGTGGAGGAGGAGGTTTTGCAG
GCGCTGGCGTAACCGGCGGGATTTGGGGATTATTTGGTCGGTGACCTTCTGCAAGAATG
CGATCAATGCAGCATCCTTAGCAGCGGCCATGGATCTGTCCCGAACTAGGATTTCGTGCT
CCCGATTCATCCTGGCCGTCTCCTGCACCCGCCAGGCTTCCTCCCTGGCCGTCCGATCCT
TCTCTCGATTCTCCAGAGTTTCTAGAAACTTGATCTGCAGCGCCTCCTGCTTCTCCACAA
CCTCCTTCATTAACCTCTCGAAATAATCCTTCCATTTCCTTTTCTTCCCCCGCCGCCGCT
GGATATCCTCATCGGAAGACGTCGACGACGACGAAGTCGTCGATTCCGATCGGAAGATGC
GAGCTTGCGGCGTAATCTGAGAATTTGTTGCCGCAATTGTGTGCTGGTGCTGGATGAATT
GCGGCGGCTGAGATTGCTGAAATTGAGGAGAATTCAATTGGGGATTTGTGATTGGGTGAA
TTACACTCAAAGGGGTTGGACTGATTGATGAAACAGTAACATGTGATGGCATTTGTAGAT
TAGGAGGAGGGGCCGGAGCCGCTGGAGCCGCCGAGCGCGGCGGCGCCGGGTAGTTTTCCA
GGGCATCCAATTGCTCAAAAAACCGGTAGCTTTTGCCGTCGGATTTGGCGGCCCGGCCGT
CCTTTGTACGCTTGTGGTATTTGTACACGTTCTCGAACTTCTCCTTGCATTTTTTGGAGC
TTCTTTGGAATCCAAGACTCGCCATTTTCCTGATTATTATTATTGTTATTATTGCAACAC
GGGCGGCGGAGTAAATATAGTTCTTAAAATTAAAATTATCTTGCGGAGTTTAAAAGTGTC
TGAATGCATAAAATAAGGGTTGAGGAATTAACTGCAGTGGTGGTAAGAAAACAAAATACA
AAGAGATAAAAACAAAGGGGAATTTCTTCAAAGTGGAGGCGAAATCCAGCTGAAAAAACG
CTTTCCCTATGGTCCCAGAAGGGACAGAACCACGTCCGATTCAAATCGTGAGTGTCAGAA
TCAGAATTCAATTCAGTTAATTATTTCTGATATCCATAATTTCAGTTTTCTGCAATTCTA
AATTTCTAATCAATTGAAAAGAAGAAGAAAGATTGTTAAAACTGAATCCTATATTCTACT
CATCAAATCAAAACTGGTATGCATAATTGGAAACCCTTTTTCTTCAAATTCCCAAACAAA
ACCCCGATCAAGATCAATTTTTTCCCCATGAAATCTCGAATTCAACATCCCAGAGAAAGC
AAAAACCAAGAAATGATTCCTTCCTTGTTTTATTTTTTCTAAAATAAAGATATAAGATCA
ATGATTACACAATTAAATTCATACCTGGAAACTTCTTCCCATAATGGGCCTTTAAGACTG
GAGTCTCTAAAAGCAGCATCCATCTCGGATCGAATTCTTATCAAAGCCAATGTTTCCTCC
CTCGGCCACCTGTTTCCCCCTCCTCCTCCTCCTCCTCCGCCGCCGCCGCCACTTTCC
TCCACCGCCACGGAGCTGGTGCCGCCGACCTCGCTGCTCCCCCCGCCGCTCTCGTGGGTC
TCCGCCG
>RMYB57
TGTGGGTGTTGGGATCGATCCCTTCGTTCATTAGCTTCTTCTTGAGGGATGAGTTCCAGT
AGTTCTTGATCTCATTGTCTGTCCTCCCGGGCAATCGGGTTGCAATTTGGGCCCACCTGT
TGCCAAGAGATTCGTGTAATGAAAGGATAAGATCCTCCTCCTCCTGTGAAAACATCCCTC
TCTTCAAATCAGGCCTCAGGTAATTAATCCATCTCAGCCTACAACTCTTCCCACATCTCT
GCAGACCTGCCAATTTAGGCACAGAGCTCCAGCAGCCGACGCCGTGCCGCGTAATGTGGT
TGAAAAGCTTCTCATCCTCCTCAGGAGACCACAGGCCTTTCCTCAGCTTCTGCTTCACAC
AGCAAGACTGGTGCCCCATTCTTAATATTTTCTCTGTCTATGTGGAGACAGTGGAAGTTC
ACTGAATTATGACAGCAATGTTTTGGTCCTAATCTCCTGTTATTATATTTTTTTTCTTTT
AAACATAAGCCAAAAAAAGATGCTTAAAAAGACATCTGATCAGCCCTTTGTGGGGGCTTT
TAACTAAAAAGGATGCTTAGAAACGAAGTGATATTATATCAGAGAGGGGGAGGGGGCAGT
G
>RMYB58
CTCCATGTGGTAAAAAAAAGTACAAATTAAATTAAATACTGAGTTATTGACCACTCCCTC
AATTCATTTATTTATTCCCAAAACGATTAAATAAATTTTCAACAGTAACTTTACAAAAAT
TGAATAAACTAATTTTACAATTCACAGTTTTACTTTTACTGCTCCACAACGGTTCGATTC

GACCCCTTTCCAGGACCGGGGCCGGGCCGGGCCCCTCCATGTGTTTCCCTGTCTTCCAAA
TTTATACACTGTCTGATTACCAGCAAGCCCGACCCGACCCGCCAGCCCAAATCTCTCATT
TTTACCCTCTTTCCGCCGGCTGCTTGTCTCCGACCATCTCGTATCCCTGATCTTCTTCAT
CATCATCGCCGTCATCGTCGTCGTCGTCGTCATCATCGTCATCGCGATCGAGATCCATGG
CGGCATCATCCTGTGGCGGCGGCGGTTGCACGGCGGGGAGCGGCCATTGCTGCTCCGGCC
GCGTCATCATCGGCGGATTCTCCGGCTTAGTATCCGATTTCCCCCTCACTTTGTAAATGG
CGTCAAGCTGATGGAAATAGGGACAGGTTTTGGAATCCTCCGATCTCTTCTTGCTGCTCT
CCTTCACTTTCTTGAAGTACTTGTTAATATTCTCCCATTTCTCCTTGCACCGCTTCGAGC
TCCGATTGTAGCCTAATTTCGCCATCGCCGCCGAGATTTCCTCCCACAGTGGCCCCTTCG
GCCCCGTCTCTTGGTACTTGAGATCCAGATTCGTTCTCAGCTTAATTAGGGCTTCGATTT
CCGCCTTCGGCCATCGGGATGAGCTTCCCGGCATTGAATTATCCCCAATTTTATCGAAGC
TTTTAATCGGGGGCGGCGGCGCGGGCGGAGGTTGTGGAATTTTCTGAGTTATTGGGAGGC
TGATCGGAATTTGCAGATTCTGCTGATCGGTGACCTTTTGCAGGAAGGCGATGACGGCGG
CGTCCTTGGCGGCGGCCATGGATCTTTCCTGGACGAGGAGTTCGTGCTCCCGATTCAATC
GTGCCCGTTCCTGCATTCGCCAGGCTTCATCTCTGGCCGTCCGATCGCGTTCGCGTTTCT
CGAGAGTTTCTATACACATCTTCTGCAATTCCTCCTGCTTCTGGATCACGTCCTTCATTA
GCCTCTGGAAGAACACCTTCCATTTCCGCTTCCTCCCCGACCCGCTACGCCGCTGCTGGA
TATCTTCGTCGGAGGAGGTCGACGACGACGACGTCATCGTATTGGAGAACAGGTTCGGCG
GCGGCGGCGGACGTCTCAGTGCGTGTGTGTCCGCCGGATTTGAAATATTCACCGGCGCTA
GCGGTGGCGGGGGAGGCTGCAATGCCGAGGGTGGCGCCATACTTACTGGATTGGGGCTAA
TCGACGATGGAACAGTTGATTTAGGCAAAAGTGTCGGCAATGGCGGTGCAGCCATTGATG
CTGGCGGCGGCGGAGGAGGTGGAGGAGTGTTTTCCAGGGCTTTTAATTGATCAAAAAAGC
GGTAGGTTTTGCCGTCGGATTTTGAGGAACGGCCGTCTTTGGTTCGCTTGTGGTATTTAT
AAACGTTCTCGAATTTCTCTTTGCATTTCTTGGCGTTCCTTTGGAAACCAAGCTCCGCCA
TTTTCCTGACAAGGGTCGCTGTGTTATTATTGCATTCTTACATTCTTGAAGAAATTAACC
GATCACTGATCCAGGCAAAACAACTAATATGTACATACCGTACAGATGAGAAATTGATCC
AATTGATCCAAGAAATATTTCTTGATGAATTTGGTTAAATTTAAAAATTCAATCTTTTTG
CTTTTACATTCGAGTCTAGTTTTTTTGCTATAATTAGCAACAACGTGGTGAAGCCAAAAC
TGAACCAAAAAGGGCCAAAGCCTGGAATTTTACATACAATACATGATACATACATAAACA
CCTATACTTATATGACAGAAATGTGTCTCTGTGTGTGTAAAGATTTACTTGAACCTGTGA
AAAAGCTAGTGAATTTATCTTTTGAATGAAAGTGGAAGACAGAAACCCTTTCAATCAAAA
AGTAATAATTCAATGGAATGGGATCAAAGATCAGTCAAGATCCACAAACACCTTTTATGC
CAAATTCAAGAAAACCCTGACTGCAACTTGAATTTCTTTAAATCTGACATACATATACTT
ATATATATACATGTACTGTATGTTTATACCTGGAAACTTGTCGCCATAATGGACCTTTCG
GACTCGAGTCTCTAAAAACCGCA
>RMYB59
ATCTGTCCTTGGTTTTCCTGAAGTATTTGTTTATGTTCTCCCATTTCTCCTTGCACCTCT
TTGCACTTCTCTTGTACCCCAATTCCATCATCCCTTGTGAGATTCTTTCCCAAAGTGGAC
CCTTCATCTGCCCTTCTTTACTGTTGTAGCTCACCTTGCACCTCATGTTTATGAGGGCCA
ACACTTCTTCCCTTGGCCAGCGGCTGGATATCGGGTTTGTCTTTAAACTAGGGATCAATA
ACCTCGACGGTGATGAGCCTGTTTGGATAACCTTGTTTGCATTAATGTGTTGATCTTGGA
ATATTTTGAGGAATTGGATTATGGTGGCCTGTCTTTCCTCAGCATTGGCTTGATCTTTGG
CCCTCATTTGCATCGCCGTTTTGCTTCTTTTAATCTCTTGGATCTTCCATTCTTCTTCTC
TGGCCATCCTCTCTTCGTCCCTCTTGATAATACTTGCGATTAGCTCGTTGTGTAGCTCCT
CCTGTCGCGCCATGACCTTATCCACAATGGCCTCAAAATAGCCCCTCAACGCGCCCACCT
CGCCTTCTTTCCTCTTCCTTTTCGACTTCCCTTCTCTCGTCAATTGCTGGCTAGCCTTTT
TTGCTGCTTCATTATTCTCAGACACTTTAGCCACATCTTTATCTGCCCTCTCCTGATCAT
ATACTACAGGGACATCCTTTGTTTCCGCAGCCGGACCGCATTCGGCTGAAACCTGAATTT
CTTCTGGATGATCATGAAGATAGGATTCATCAAGCTCATTGCAAACCCTGCTGTCCTTGC
AGCTTATTACATTGTTGAAGTTCTTGCCTTCAAATTTTTCCCTACACTCTACTGCGCTTC
TCTTGAACCCAATCTCAGCAAGCCTCCTTGAAACATGTCCCCAGCTGATCAATTCTGGCA

GGGATGTACAGCTCTCCATGCTAGATCTGAACTTAAACAGCTCAACAACTTCTTCATTGG
ACCAAGGCACCACAGATCTCTCCCCTTGGGTGATACTATTATCAAGAGAAGAAGAAATTA
GGGAAGGGTCGAAACAAGGGTTGCAGTGAATGTGATCATCAAGAAGTGATGATGTTCTTG
AAGCATTAATGAATTGGCTCAAGTAGTCATGATGATCACTGCTGCACATTATTCCAATTC
CATCAAACATGGTTCAGATTTTCCCTTTCTCTTAGCATCTTACTAATAATCTCTGCTGCA
CTAAAAAACCTCTGAAACCAAACCCTCGCTAGCTGCCTACCTACCTGTGTTACATCAAGA
TATTATTTTTAGATATGACAGCAAATTAAAAACAAAAACCCCATTTTTATTTCACAAGAA
AATAAAAAATCAGCGCCGAATACATCCCATCCCACCACGAATTAGTGG
>RMYB60
TTTTTGTTGAAATTTAATTTAATTTTTTTTCCTTTTTCTGGGTACTCTTTTCTTTTTTCT
GTTTACAATTTTTCCCATTTTTTCCCTCTGCCTGAGATTGATGGATTCAAATCTCCAAAA
AACAAACAAAAGATGCAACCTTTGAGCTCTTTCTCATCATCCCTTCTTCTCCACCACTGT
ACAAACACCAAAATTATTTCTTCCCTCCATTCTCTTTTTATATTAACTTAATAAAGAAAA
CCCTCTCTGGATTACCCAAGAAACAGGGATCATAGTTTCTTGACACTCTTCCTGTCATCA
TCTTCGTCAACTTCGCTGATTTCTTCCAGTTTTTTCCCAATTAATTCATTAATTCATTCA
ATCCTGTTGATGCCAATTCGCTTCATCGCGGCATTCCTGAATTGCTCGTTGATATTAGCT
TGCTGCATACACATGTACTGTTGTTGCTGCTGCTGCTGATTGTAGTGCTGCTGCTGAATC
TGTAGCTGAATGTATTGTTGCTGATGCTGCTGATTCTGTTGGTGTTGATGCTGGTGCTGA
AACCTTTGCTGTTCTAATCCCATCATGTAATTCCGCACCTCCGTCCGTATCATCTCCTGC
ATCACAGCCATCATTTCCGCGCTGAACGGCGCAAAGACCTTGTCTTGAACCGGCGGCGGA
CTCGGCACCTTGCTAACCGGACTCTGCGGCTGTATCGCGATGGGAATCGCCGCCGGGGGT
GTCACCGGCGGCGGAGGAGGCGGTGGCGGAATATTAGGCTTTGCTTGAACGGCCTCAATT
TTGAGATTGGGAGATTCAATCGTCTCCGCTCCGGGAAGCGAAAGGCTGAGGCTCGTCGGC
GGATCATTCACCACCGGTTCAATCTGAACCGGCAAAAGCGCGCCGCTTCTCGCCACCGGT
TTATACACAAACGAACCGGACATAACCGGGAGGCTCGAATCGCTCGCGTCCGACCCGGAC
GGGCTGCCGGGTGCCAAGTACAGGCCCGACCCGGGCACGCCGGAGCCGGCGCTAACGGAC
CTCTTCAGAGGCCGATCCGGCGGCTGGTCGGCGCTGTCAAAAAAAGCCTCGTCGGAGCTG
AAGGAGGAGCACTTGCGCTTGAGGGTCGAATTCCAATGATTCTTGATAGCGTTGTCGGTC
CGGCCCGAGAGGAGTCGGGCTATGGTAGCCCATTTATTCCCGAACTTGGCGTGGGCCCGC
ATGATGGTTTCATCCTCCTCCGGCGTAAACGCCCGGTGCTCCACCTGCGGCGACAGCTGG
TTGCACCACCGCAGGCGGCAGCTCTTCCCGGATCTCCCCGGGATCGATTTGCTTATGAGG
GACCAATTCCTCGGGCCATGCTTCTGCACCAGTTGTTGTAATAACTCGTCTTCCTCGGGG
CTCCAGGGACCCTTGATCCGATCCAAATCCATTTTCTTACAAAACTCACCACTACCACCA
CCGCCGCCTCCGCTTGTAATCGCCGCTGCCATAAATCTATTCAATCTTGTCCTAGTCTCT
ACTCTAAAAGACCATGAAATTTGGGAGGAAATTTTGAGGTGGTGGAGAGATTTTTACATG
AGGGTGTAAGGAATGGGGGGATTGGTATATATATGGCCATATTGGTTGGGG
>RMYB61
TAATAATAATAATAATAATAATAATAATAATAATAATAATAATAATAATAATAGTAATAT
TTTTTTATATATTCAATAATCGAAATAAATCAATCTCCATGGAGCAAGTGCATGGACCAA
AGGTCTTCCATGCTCCAAAGGTTTTCATTCGAGTCCGGAGTGGGGAAGGCTGGGGCCTGG
AAATGTTCCATGGTCCCAGGAGTGAAAGAACATGCCGGTGAAGAGTAGGACATTTGATCA
ACAACATGATCCACGGGGCCGTAACCCAAAGAAACTTGACTTGTGTTACCGTGATTAGCC
ATATTACTATTGTTTGTTTGTTGTTGCTGCTGCTGATAATCAGATGAACTTTCGTTTGCT
GGTTGTTTTATGTGTTTCTGAATTCGAGTTCTCCAAAAATTCTTGATCTCATTATCTGTT
CTTCCAGGGAGATATTTTGCAATCTTTGACCACCTGTTCCCCCACTTAGCATGAAGATCC
ATGATCAGAAGCTGCTCCTCCGGCGTAATATTCCCTCTCTTCACGTCGGGCCTCAGGTAA
TTCAGCCACCGCAGCCTACAGCTCTTCCCCGTCCGCTTCAGCCCAGCAGATCGAGCAAGT
GTGTTCCAGACGCCTTCGCCATGATTGGCAATGTAGTTCATGAGTATGAGGTCTTCTTCC
ATCGTCCACGGGCCTTTCCTCACTTCCACCTCTTCAGATCTGCGGCATGCCTTCTTATTA
TCCATTGATTGATCTCACAAGCTATGGCTACCTTGGATATCGAAGATATATATGTGTGTG
TGTATATGTATGGATGAATGTGTGGATAGTTGTGTAGAGAAATTGTTGAATTAATGGGTG

TTTTAGTTATTTGTGTATGTGTGGTGTGAA
>RMYB62
CCGAAAATCATGTAATGGGCAAAACAATAAATATGAAGTACTTAAATTCCTCATCCCTTT
TCAACCAGAGTCACCAAATACAGTATGTTCAAGTTGAGTAACACCACTATGGTCCATCGA
GTTTAAAGATTGAAAGTAGAGCCTCGAAATACGTCAAAAGTACAACAGCTCCCACCATCC
ACCCCTTTGCATCATTTAACAAGGCAGCAAAACAACAGAGTAGGAGCGTAGCAGCAGTGA
ATGGCCAAAAAAACAAAAACTCTCAAGATAGCTGCATTTCCCACCTTTTCTCAAATCCTT
GATCTTGCCAACAAAAACTAACCATCCATGGCACTGTCCAATTCGAATGAATCACCTGTG
ACACTTGCTCCGCCATCTCTTCCATGTTCCGTAATTGGCTGGATGTGCACATCAGCAACA
TCCAGTTGTTGGCTTCTCGTTTCATCAATGTTGTCCACTTCCATTGAGGTTCTCTCATCC
GAAATGACAGCAGGCTCAGGGTTTCCAGCATCGGAAGAAACTGATTGATCCAAAGCAGCT
TCAGGCTCGACCGGTACCACATCATTGTTTTCTGCAGCAACTTCATGATTGGAAGTTTCA
AGATCTGGTGCAATAGATTGATTGGTCGCAGCATTAGCAGCTTCGTCCAATACAAGGACT
TCATCATTGGGAGTTCCGTCATTTGGTGCAACAGACTCATCAAGAGTGTTAATCTCATTG
ACTTGAGGGTCATCATCTTTCTTCGCAGCATTTTCTGCTTCCTTTTCTGCTTGCAGTCTG
TATGCACCTACCAAATTCTGAACCCTTTCTAGTTCGGACATTAGATCACCATATCGCTTC
TGTAAAGTGTTCTCGAGGTCCTTCTGCTTCTGAACCTCTTCCCAAAGATTTCCAATCCTG
TGTGTCGCTGCGAGCTGCTCTTGCTTGTGCAGAACTTGGAAGCACTCAAGTTCAGTCCCT
GCAGTGTCCATTTGCTTGAAAGTTGCCTCAATTTGAGTCGAGAGTTTCCCGGCTCGCATT
TGATAGCCGTTGGTAAGAACTTTTATCCTCTGCTCTAGCTTTTGTGCTTTTTTTGTCTCA
TTGTCCATCTCCTTCTTCACTTTATCAAATTCATTCTGCAAAGCAGCAATTTTCTCCATG
TTGTTGGCAACACTTGATAGACCATAGCCGTCACGGGTAGGGAAGTACATCATGTCATTT
AAGCATGTCTCATGTGCTTCAACATACTCATCGAGAGATTCATCCTCATGGCCCATTGCG
ACACGAAGAACATCAGATTCATTCTTAATTAACTCATCAGCCTCTTTCAGTTCATTTTCT
TCAAAGTCGTCAATTGAGGGCACAGGAGCAGATTTTGCTTTGGCACTGCGCTTGCTACCT
TTCTTCTTCTCCTTGGCGACTTTCTCATCCAAGGGATATTTTGTATTGTCATGCTCTAGC
AAGGATAGTAGCTCCTTTCTTATTAATTCATCAGCTTGCTCAATTAATGTCGGAGGGACA
AAGGAGCTCTTGTCTTCATCAGCTCTGATCAAAGAGTTTCTAATAAGGTCCAATGAAGCA
GTAGGTGGTCTTGGTAGCTCCCTCTGCAGAACTTTTGATCTTTTCTTGAGTAATGCTTGT
AGCCTTGCCTCTTCCTCTGTTTTCTCTCTAGCAGTTCTATCAGACATATCTTCCTCAATT
TTCTCTTCTGGTTCTTCTTCGTCTTCAGCAAAGGGCTGGATAACTATTTGGTATTCATTC
TTAGGCTGTGGAAGATTTTTCAATCCAGAAAGCAGTTCTTTTTTTGAATCAGCTTGCCTT
AATTTGTCGCTGTCATGCACATCCATGTCTTCATTAATGTGCAGCTCATCCCTCATTGGA
GTTCCTTTTGGAGTCATACTGAGTGAATATGCATCACGTGAAGGGGTCGTCCCAATTCTA
GGAGTAAGTCCTTGGCCTCCAGGAGTTGCTGAAGGCGTCAACAGAGGATTAGGTGTTGCA
ATATCCTTTTTCTTAGGTGTAACTCCAGAAAAATCTGAAGGGTGCAACATTGGATTATCT
CCACCAAGCAAGGGAGTCTGAGATAGAGTCAGCCTGCGTTGATTTCTGCTTCCATCATA
ATGGCATCCTGCTTGCCGGCAGGAGTTCTCTGAGGGGTTCTCATCGGGGTCATACCCTGC
CTTGGAGTCTGAGCATAGCTAGCAAGAAGAGCACGTGTTGCTGCATTCCCTTCTGTTAGC
TCTTCATTTCCAACAAGATCGCTAGCAATGCCCATCTTGGCAATAGCATCCAGCTCATGG
TCTGGAATCTGCGGAGCAGGGAGATTTAGTTTAGACCGTTTGCGAACTGTTTCAGGATCA
TTAAGTTTGTTAGCTTGCATGATTGCTGCTGGAGTATCCTGCCTCTGAGCTATTTTATTT
TTTGCAATATCCTGCTTTCTTAGGCGTGCTTCTCTATCTACTCTCCTTTCACCTTCAAGT
TCTTCTATTGTGGTCGGAAACTTGACTAATTCAGTAGGTCGGTCTTCTTCAGTAACATCA
TAAAACCCTGGAGGCGGCCTCTTTTCAAAGGGGATTTCAGCATTATAATCAATTCCTCTT
CTCTTTCTCTTCCTGTGCCGAACATCAATGCCAGCTGCTTTTAGTTCTCTACGTTTCTGC
AATGAAGCAAGCCTCCGAGCCTCTTCAAGCTGTTTCTCTCTCGCTTTTCTTTTTGCTTTC
TTTCCTCTAGTATTAGCCAATCTTGCCCGTGCTTCAGAAAGCATTTCTTTCTCATCTTCA
TCCATATCCACAGGATCAGGACGTGCTGGCTTAGATTCTGGGTTGGGGTCAATCTCTCCA
GGACGCAATTTTCGTGGATCATCTCCAGGTTCATAGTTCTCATCTTTAGCACATGCTGCA
TCTAGGAGTTTCTCATACCTCTCCAGGCATTGAGATGGAGTTCGCCCAACAATTGGGGCA

ATAGTTCTCCATTGAGTAGGCATAAGTTTGCAAGATGCAATAATTTCTCATCTTCTTCT
CGAGTCCACTCAGTCTTCTTAATGGAGGGGTCAAGCCACTCGTACCACCGCGCCTTGCAT
TGCTTCGCAGATTTTCGAACCAAGAGCGAAGAAATTCTGGCCCACTGATTTTTCCCATAC
TTCATAACCGCCGCCTTCAAGATCTCGTCCTCCGTGTTCTTCCATACTCCACCCTTGATC
ATAATCCTCATTTTGCCGGCAGAATTGATGAAGACGAGGAGCAGAGAATGGATTGAATTT
GATTCGAAAAAGGGGTTCTTTCAGGTTTCTCCGTTCCCAGTCGCAATGGCTATTGGCTAG
GGCTGCTGCAGGTTTACCATGGTGGATAGGTGGGTCTTTCCGAATTACTACTACATGACT
AGTACTACTGCGTTT
>RMYB63
CACCCGCAGTTTTCATTTTACCAATACTTCAACCAGCTTTTGCAAAATATGTAATGAATG
CATGATGAAATGAATCAAGCATAATTTTAGCTATGGAATAACTTCCAGTACTCTAATACA
TTAAAATCAACAAGGCGACAATACCATCAGTTACATGAGAAGTGCATCTGAGCACTAAAA
TTATTGAAAACAAAGAACCACCAGAAACCAGAGACATTGACACGGACGGATCCATAAACA
ACGACTTGACCATAACAGAAACCAGACCACAACGCAAATCTAAACAACCATGACAACTTG
AATCCAGGCACAAATCTGGAACACAGAAAGCGCTTCACATTACACCTAATTTGAAAATGC
TAAATAAGCATTATACTTCAACAATAACAGAAACAACATTTTTCACTCAGAAGTCCCAAC
GCCCATAAAGTCGAAGAACTGAGGCTCTTTGTGGCCGGCTCCTTGCTGGGGCTGAGCAAC
CGGAAGACAGATCCCATGGGAAACAACAGAGTTGGCATCAGACATTATGGGATTTCCATG
ACTAGTCATGTGACAGTAATAGATGTTCAACCGATTAGCGCCAATAGGACGAGAAAGTTT
GTTCACTGTTCCACCCTTGCCATGGCCTGAACCAGAGCCACCAGATATTAAAGGACTTGC
TTGTGCATTCATGGGTGTGCTAGTGTAGGGCGTGAATCCAGGGTAGTAATTTCCTGTGGG
CCCACCCTTGAGAGGGACATGAGAGTCAGAGCCGGAAGATGTGCCCATGGTTCTCTTGCG
ACTTCGCATCAGCACATTGAATTTATTCTTCAATTGATTATCAGTTCGGCCACGGAAGTG
TTTGACAATTAAGGACCATCTGTTACCAACTTCTTTTTGCAAATGTAAGAGCCTTAGCTG
CTCTTCTTCAGTAAAGGGTGCCTTACAGACATTGGGACTCAAATGATTAACCCAACGCAG
GCGACAGCTTTTTGCTGTTCTTCCTTCGATACGGTGCGCAATGAAATCCCAACCTCCAGC
ACCAAACGCTTGGACAAATGTAGTAAGGAGCGCATCTTCCTCGGCTGTCCAATGGCATTT
GCAGTAAGGCTTCATCCTTTTCCTTCCTTCAGCATATGTTTTATTTCTGGAGGTCATCAC
TCCAGAAACTGACTACCCACAAGATGCTCTGTGGCTTCAATGAAAATTTCCATCCAATATA
TAGAGGACGGAGAAATAACTACATACATAAATGTTTATATGTAAAAAAATAAATTGCCTG
ACAATGAGGCCTGATTACAAAAATCAGCATGAAAATTCAATAAATTCACCCTCAAGTCAA
ATATGAAACGGAAGAATAAAGCCCTCAATGTCCTTGAGAACTCTCTTCCATGTGTCTCCA
TAATTTCAGAATAGATATACCTCACATTTACTCGAAAAAACTCTAGACGTGAAGAATGAA
TCCTTGGTTAAATTATTTTAAAAATAATTATTGCGAGGGTTTAATTAAGGTCATATCTAA
TATACAATCTAGCGCAATTTCCTCCAAATAACACTAGGAATCTAATTATTGTTAACTCGA
AATAATGTTTAGGCGAGCACTGCTAATATTTCCTTCAAATACAATTCAATCAAAGGAATA
AGAGGGAAACCACCAGCCACTTTATGGGGCAGTCTAAAGGCGACTTGGGTGACATGAATA
ATCATTTTTGGGTGTTGCAAGTGCAGGCAAATTTTGATGTGCACTTAAATCTTATTAATA
TAAAAGTTTCATGTGAATGTATTTTTACATCCTAAGAAATTATTAACATAAACTACCACA
GGCAACTTTTTAAGAATAACATTCATCCCATACATGAACATACAAAAATTTAACAATTCA
ATCATTATTCAGATTTAAATTCAAATTATAATTTATTTATACACAAAAAACTTATACGTA
AGACAATTCATAACAACTGATATGATATATATATATATATATAAAATATTTTTATGATGA
AACTCCAAATTTGATTAGTCTCAT
>RMYB64
TGTGGTTTTGTCCTCATTAAGGTTTGGTGACTGATCATCCGCTAATTCTGTTGGCTGCGG
CGGCAACTGGTCGGTGGTAGTAGGTGGCGGAAGAGGCTTGTGGGTGAGAGGGTCGATTCC
CATTTTCCTTAGTTTCTTCTTGATGTGGGTGTTCCAGTGGTTCTTTATCTCGTTGTCTGT
TCTTCCAGGCAGATGTGAAGCAATTTTGGACCATCTGTTTCCAAGCTGGGCATGGAGATC
AATGACCATTTTCTCCTCATACTGGGAAAGAAGCCCCCTCTTCAAGTCTGGTCTCAGGTA
ATTTGTCCATCTCAATCTGCAGCTCTTTCCACACCTCAGAAGCCCTGCAAGTTTAGGGAC
AGCCCTCCAGCAGCACTGGCTACCATTTGTGAGCATGAAGGCGATCAGCTTCTTGTCTTC

ATCACAAGTCCATGGCCCTTTCTTCAGTCCCACTTTGTCACAA
>RMYB65
CCTCTATGATCCCCATGAAATCCCCATCCTGCCCTTGTGCACCACTTTCATCGAACAGCC
ATTTCTCCAGCAAACTCAGGGGCATTACAGTCATCCCCAAATTCGACGAATCGAATCCCA
CCTCACTGGGGCTCTGCAGATTATTATTGCAGCTCTCCGATGAGCCGGCGGCCTGGTGCA
GTTTTGGGGAGTCATTGCTCTTCCAGTTTTCCAGTAGCCGGGCGATGTTGTCCGCGTTTA
CTGCATACATAGATGTCTGAATTGGCCTAGTTTGGGTCGTTGGACTTGGGTGCTGATCAA
CAACGGCGGCAGAATCGGATTTATCGAAGGAGAGAGCCTCAGAGAGGGCCTGTTTGGCCG
TCTGAATATCGGTTTGCAGCCTCCTCTCCCACTGACCTTTGGAAACGGCAACCTGTTTCG
AACGGGAAGAATTATTATTTCCATCCACACAAACATGGCCTTGAGTTTGCTGCTGTTGCT
GCTTCCCTAGCTTCTTCCTCAAGTGAGTATTCCAATAGTTCTTGACGTCGTTGTCGGTCC
TGTGGGGAAGATAGGAAGCTATTGCAGCCCATCGATTGCCTAGAAGGGCTTGGAGATGGA
TGATCATCTTCTCCTCGTGTTCGGAGAAGTTGCCACGCTTGATGCCCGGCCGGAGATAGT
TGGTCCACCGGAGCCGGCAGCTCTTGCTGCATCTCGACAACCCTGTGTTGGAAGGAACAG
ACCTCCAATTGCCAGGGCCATGTTCGTGAATGTAAGAAACGAGGATGATATCTTCTTCAG
GAGTCCATGGCCCTTTCTTCACACCAATTCTCCCACAGCAAGGAGCTACTGGTCTGCCCA
TTTTTCTTTCACTACTGCTACCTACTAGAAGTATATGTATATACACACTACTGTGACTAA
AAACAAGATTAGTTGTGTCTTGTGTTTAATTCCCTGTTTTTGGTGTTGTTGGCTTCCACT
TATAATGGGTAGGGATTTATATACATGGGTTGTGAAACAAATTTCAGAATTGGTTGCTGC
TGTTTTTCTTTTGGGGATTTGTGCGAAGGATGGAATGAATGTGGGGTGCAACTCTGATTG
GCTATCAGATC
>RMYB66
TCCCTATCTTCTTCTGCTGATTCAATTGGGATCTCCGCTTCAGCGTCGAATTCCAATGAT
TCTTAACCGCATTGTCCGTCCTCCCCGGCAGCAGCCGCGCGATCGTCGCCCAGCGGTTGC
CGTACTTGTCGTGCGCCGCCAGGATGGCCTCGTCCTCCGCCGCCGTGAAGGGGCGGTGCT
CCACGGTGGGGCTGAGCTGGTTGCACCAGCGTAGCCTGCACGATTTCCCCGATCGGCCCT
TTATGTATTTGCTAATGAGCGACCAATTCCGGGCCCCGTACTTCTCCACCAATCGCGTCA
GGATGTTGTCCTCCTCCGCGCTCCACGGCCCCTTTATCCTCTCGGACTTCCTCCCCGACA
CCGTCGATGAATCCGACGAGCACGACGAACTCCCCGACGAACATCGATTCAACATTTCCA
TTCCCCAAATTTTCTTACAAGAAGTCAAGAACCGTTTTTTTCTTTCAGACAAAGGTATTG
CAAGTGGGAT
>RMYB67
GTTCAATTAATCTTTCCGTTTTCTCAAGACAAGGGGTAAGAAAACTAACTGCTTTCTTCA
ATGGAGTTGTCGAAAATGCTTCGCCTTTTCTTGCTCTGCACCTCCTTCTCTTGACGCTCG
TAGTACTTTTGAGCATGGCTTGCTACCTGGGCAGGGCTTCTTGTCCTAACAGAATACCTG
GAAATGCTCTTCCAATCCCCTTTCCCATATTTCTTTAATCCAGCCAGAAATGCCTTGTGT
TCGTCGTCGGTCCAGGCTTTGGCCGCCTTCCGCTGGCCGGTGACCGGCTTCTTCTCGCTT
TTAGGGTTCCGGCGATCGGCCTCCGACGACGATCGCAATTCAATTTCATTATTCACCCCG
TATTTGGGCAAATCGACAAGGCCGGCGTCGATCGCCGCCACGTCTTCCAACAACACGGCG
TAGTGGCGCTCGACCTCGGCCGGCGTCTTGGTCTGCATTTTCTCGGCGATCCTCACCCAC
CGGTTGGGGTAACCGTCGGGGAACTCGACCAACCCGTCCTCGAAATCCTTGTCCTCCCTC
CACGTCCACGTCGACGGAGTGGCGTGGGCATCTCGATCGGCGGAATCCATTTTCCCCTGC
GTTGTCGATAATCGATTGGAAAACAAGAGAAGAAATGGAATTGATAATTTGATTGATGGG
ATTTGGGGAATTAAATGGGAAAAATAAGCAGAGAAATTGGTGCAATTACTGCAGAG
>RMYB68
AAACATGAACTCTCCGATTCATAAATTATGGCAGCAGAGACATCTCTGTTATCCAATCGC
CTCAAAGCTTGAGGCAATCGGGCACCGCCGCCGTCGCTCCTCCCCGAGTACGACTTCCGT
TTCAGCGTCGAATTCCAATGATTTTTTACGGCGTTATCCGTACGTCCGGTGAGCATCTTC
GCAATCGTCGCCCACTTATTCCCGTGCACCGCGTGCGCCTGAACAATGATCTCATCCTCC
TCCTCCGTGAACGGCCGGTGCTCCACCTCCGGCGCCAGCTGATTGCACCACCGAAGCCTG
CACGATTTCCCCGATCTCCCCGGAATCGACTGGCTTATCAGAGTCCAATTCCTCGCGCCG

TGCTGCTGCACAAGTTTCGTCAGCAATTCATCCTCCTCCGGACTCCATGGCCCCTTCACC
CGCTCGGGAACCGCCTCCGGAGCCACCTTCTTGGCCATCGCCGCCATTGAAATACAGCAA
CAATTTTGAAAAACCCTAATTATAACTGA
>RMYB69
GCAGTCAAGTGACAAATTAATCTTTCTCGAGGACATGGTTTTTTTCCTCAGATAAGTTC
TCCAGTAATTCTTGATCTCATTATCTGTTCTTCCTGGCAATCTTTGGGCAATCCTCGACC
ATTTGTTACCCCATTTCTCATGAAGTTCAACTATAGTTCGCTCTTCTTCTGCAGTGATGT
TACCATGCTTCAGATTGGGGCGGAGGTAGTTCATCCACCGCAGCCTGCAGCTCTTGCCAC
CCCTCTTCAGACCTGATGCTTTTGCTAATGCATCCCATCTCCTCTCCCCCAAGACAGCTA
CAGCATCAGCCAACTGTTGGTCCTCTTCTTCCAACCAGGGCCCTTTACGCAATTCGGCTT
CCTGCATGACAGAACATCTTGCATTAGCACACTAGTGGAATTTCCACCATGGGGATTGGC
TTATGTGACTGATCTTGAAACTTCCACTATGCCATTAGATGAGTTTTGAAATTATTATAT
ACAGTTGAAATTTCTTATGCTACAAATATATTGAATACATGTATGAGTTTTATCCTCATC
TGGATTACAACATCAATACCTATCTAATCAGAGATAAAGGAAGAATTTGTACCTCAATGG
ATAGCAAAGAAACCCAAAATCTTTCTTTAATCATCTATCGAATAGATTACTTGACCATTA
TCTTGTTACTCTCTCAATGTTTATGCAGTCAAGTGACAAATTAATCTGGCAGACAATAGA
TTTGAACCATGAGAAGGGGTAAAACATAGATGGAATAAAGTATAGCATTGCAATGCAACC
GCAGTTTGATTCTACTCTTCAATGTACAGAGTTCACATTGCTACAGCACAATGTTTGGAA
AGGAAAACAGAACACATATGGATTATCAAAAACAGAATGTCGTCACAGTCTACATTCTAA
GAGTAGGTCCCCCAATAGGATATGTGATAGTCAACTCCAGCAGGAAACAACAGACATCGC
GCTGCTTCTCAAGAATACCTCTAACGGTCATCAATAAATGCTTTATTCATAAGTTCTAGC
GCCGAATCCATGAACCTTCCAATTGCCTTGAGTTCACGGCCCACTGGTTCCATAAACTTC
AGGTCAACTTCAATGGGCCATACCTTAAGACCGAGTAATTTGATAGGCGTAACTTGTCCA
GGCACAATACAGTCAGGACCAGCAGCTTCGACCACAGCCTCTGTTCCTAATCCACTCCCG
ATTGGATCATCATGCTTGATAACCGAAAGGGCATAAGTGGAGTAAGGGGTCTGGGGTAGG
CGAAGAACAGGGGAGTGATGAATTTTGGGAATAGGCTTTGCAGCAGACGATGCTCTCCAT
GGAACATCTTGTGGCAGGGCAGCTCCTTTTTTCGACATTGTTGCAGTGCTCCTAGCACTT
GTCGATGATAACTGCAGGATTGCGCCCAAAATACACAAAATCACGGACTATATTCCGTAA
AAACACCAACTTCAGAATAAGGATTTGGTGAAGATAAGGATTTGTATTTCTTTTTCCCCT
AATGAAGGAAATGCCGAGCGCCCGAGCGGGGAGGTAGGCAGGGCCGGACTTCGG
>RMYB3R-1
CTCTCTTCAAAAATCTCCCAATCAATAGAGAAATCATGCTCAGGAAACTTGTCACCCTGT
TCACAAGGTACTTCTGGTATCAAAAATGCGGGTTCTTGAAATACCGGACGATAATCTTCG
GCGGAAAAGGGAATTAAACTTGTATCTTCAGTTGTTCTGCATTCCCCCAGGAGTGCAATT
GTGTTGTTTATAGACTGAGACATACTAGCAATGGCTGAACCTTGACTGCATTCAGAAGCT
TCTTCAACTTCTACTCCACCTTCAAGAATATCATCTTCACTACCTTGTTTTCCCCTTGAA
GACGAAGAAGCTGTCAGGTGATTGGAATGACTGACAAGGGGAACATCTTGGAGCTGTTGA
AGCACCCCTGAACTCATATACATGTCCAATTTCTTCCTGACAGAACTATTCCAGTGATTT
TTTATTGCATTGTCAGTTCTCCCGGGCAAATACTTGGTTAACTCTGCCCACTTGTTTCCA
TGCATTTGATGAGCACGTATCAAAATCACCTCTTCTTCTTGTGTCCAAGCATCTCTGTTT
ATGTTGGGATTGAGATGGTTGTGCCACCTTTCCCGACATTGCTTTCCAATTCGTCCAGGA
AGATTCTGCGCAATTGTAGACCATTTTTTGGGGCCATATTTCTCCACCAATTCAATTATC
TTTTCATCCTCCTCTTTGGACCATGGTCCTTTGACAAGATCTGGATTAAGAACCTTCTGC
CACCTATGTAAGCACTGTACATCTGTTCGATCCCTGAAACACTCCGCTATCTTTTTCCAG
TTTTTTCCTTTGTAGC
>RMYB3R-2
CCATTTTAGCTTTTGCGCTGATATTGTGCTACACACAAATAAATGAATAATTTCCCCAAA
AGTGAGTGAAAATAAATTTAGGCAGCAATTTTTGCCAAGTATTCGAAACTTGAAATGGGA
AAAAAGTTCCAATAATTTGGGAAGTATGTTAGAGGTTTAGGGTTTCTAAATGTGCACGAT
AAAGATACCTTCCAGGTAGAAGCTTAGCTAATTCAGCCCATTTATTCCCATGAACGCGAT
GAGCATTAAGCAGAGTAAGCTCCTCTTCCAAAGTCCAAGCATCCTTTTTAATATGTGGAT

TCAAATGATTATGCCATCTCTCACGGCATTGTTTCCCTATTCGACCTGGCAATGACTTGG
CTATAATAGACCATTTTGTAGGTCCATATTTAGCTACTAGTTCTGTGATCTTATCATCCT
CCTCTTGAGTCCAGGGACCTTTAACAAGTTCTGGATTCAAAACCTTTTGCCATCTATGCA
AGCACTGCACTTCTGATCTATCCGGAAAGAACTCAGCTATTTTCTTCCAACACTTCCCTC
TGAAAGCTGCAACTGCTTTTTTTAACGTAGCATCCTCCTCTGGGGTCCATCCTCCTTTTG
CTCTTCTAATAGGGCCAGAAGTTCTCTTGTGAGCTGGAGAAGTACTAGAAGGGCTGCTTA
TGCCGGTTGACTTTAGTGCTATGGTGCTACTATTCTCCGAAACAGAAGAGCTCGAATCAA
CTGTGATATCCTTGTTCTCGAGACAACGGTCATCAGATTTCAACCCTTCCATTAATTGCT
TCAGATCATCAAACCTCCAAATTCCCAAACTTCCTACACTCACGAATTGAAGAACCAGCT
CATCGATTCTTGAGCCCTAATAAAACCGCAAAATTGTGTGTGTGTGAGAGAAAGAGGGGA
TTCCGAGTTGCGAGAAACTTTGCCGTTCAT
>RMYB3R-3
TTTTTTTTTAACCCTCCATAATGCAGAGAAATTTTATTCCGTGATTAAAGAGAAATGTA
ATCCCTAGAAAAAATCCTGTACAGTTAAGTTACAGCTTTAGTTAAAACACAATGGAGATG
ATCTTTTTCTTGGGTAGAAAATTCAATGATACCGCCATATTCTTAAACAAAGTCAGGACA
TGCTTGACATGAGGGCTTGGCTTCTCTTCTGAAAGGTTTTTCAGCGCTTTGGCTTCTCTG
TCTTCAATTACATTTAGCAACTGAAGATTTTAAACCCCGTCTTTGACGAGTGTAGACAAA
TTTTGTTACAGGACGGGCTTCCTTGACCTTAGAGTCTCCAGATTTTGTATTGTTGTTGCA
GGCTTCTTGGTCCGTGATAGTTGTAAAATCAAGCTGCTTCTCAACAGATTTATGAACTGA
AGTCCTTTTGAAATTTAATCGATATGGTGGTGAAGAATTAGGTGACTTATTGCTGCTTAC
TCCATCTTCATCATCTGAAACAGGATCAGCACATGAAGTCTTACTTTGCAACCCGGCATT
CTCAGAGCCATTTGTGTGATTATGCTCACCACTGGGTACAACTAGTTCCTTAGGTTGCAT
ATCCAACTTCCTCTTCCTTAGAATGGAAGGAGTATTCCGAAAGCTTTTTGCAGCAATTCT
CAGTATAGATTCAGGGGACTGCGCAGAGAAGTTACCACCTCTTACACTAGGTGGAGTAAA
CAAAGGTGTAGGTGGTGTTGCGGGGCAAGTGTCTGACTGAGATTGCATCAAGGAAACATT
TCTAAGGGTTGAATCCAGTTGTCCATAATTGTCAAATGTTTGAGGCTCATAACATAGTGT
CCCATGTACGGGCATTTCATAACGCAACGATGCTTTAACAATGTTATATCGACCAACTCC
ACCAAATGTGTGTTGTTTCTCAAACTCAGATTCTTTGGTTGGATTAACAGCCCCTGATTC
TGATTGCATTGCCTCATGATGCTTGGTGCTCTCTGGATTGGTGGGCTCCATCTGTGGATG
GCTAGCGACATTATAATCTTTGGACCTCTCCTCTGGCATATCAGTCCTCCTATCATCAGC
TTTACGCAAATCAGTAGTTTCACACGAAGCTAAGTGTGCTGAATTTGACCCTTTATTTGA
ACCAACATGTAGTTCTCCAGCTGAGGCAGTCTTATGGATGTCTGCAGCAGCATCATTTAG
GGACACACATTTAGAAACAGGAGGAAGGTTCCCTGTAGCCAGATAAAAATCCAACTTTTT
CTTTAGAGAGCTATTCCAGTGATTCTTGATGGCATTATCAGTCCTTCCAGGTAGAAGCTT
AGCTAATTCAGCCCATTTATTCCCATGAACTCGATGAGCATTAAGCAGGGTAAGCTCCTC
TTCCAAAGTCCAAGCATCCTTTTTAATATGTGGATTCAAATGATTATGCCATCTCTCACG
GCACTGTTTCCCTATTCGACCTGGCAATGACTTGGCTATAATAGACCATTTTGTAGGTCC
ATATTTAGCCACTAGTTCTGTGATCTTCTCATCCTCCTCTTGAGTCCAGGGACCTTTAAC
AAGTTCTGGATTCAAAACCTTTTGCCATCTATGCAAGCACTGCACTTCTGATCTATCCGG
AAAGAACTCAGCTATTTTCTTCCAACACTTCCCTCTGAAAGCTGCAACTGCTTTTTTTAA
CGTAGCATCCTCCTCCGGGGTCCATCCTCCTTTTGCTCTTCTAATAGGGCCAGAAGTTCT
CTTGTGAGCTGGAGAAGTACTAGAAGGGCTGCTTATGCCGGTTGACTTTAGTGCTATGGT
GCTACTATTCTCCGAAACAGAAGAGCTCGAATCAACTGTGATATCCTTGTTCTCGAGAGA
ACGGTCATCAGATTTCAACCCTTCCATTAATTGCTTCAGATCATCAGACCTCCAAATTCC
CAAACTTCCTAAACTCAAAAATTACCAAATCCAATATATTAAAAGAAGTGCAAACGCCCA
TGAAAATTCTAATTCTAATGACCAAAAAAATTTCAGCACTAATCCTATACCATGTCGAGT
CCAGATCTCAAAAAAAAAAATCGATAATTAACAAAACAACCGCCTAATTCACAAAAGAATC
TTAACACATAAATCGCCCCAAAAAATTAGAGAGGAAAATCTCAGACAATTTTTCGGTTAA
TCACAGCTCGAAAAAAGACACAAAAAAACATACCTAAAACGATCAAATAACGAATTAAAA
AGACACAGTAGATAAAAAATTACAAAAGTCGAAAGTTCAAGGATCTTGAAACAAATTACC
CACCAACT

Part II. the protein sequences of RMYB gene family.

>RMYB1
MEIYACNRWMHMPDSPAPPWNRQEDKIFEKALVEFPDGIDDRWQRIAGFLPGKSPDDVRA
HYEALLHDITEIDSGRVELPSYSDGSTSIECERGSSSSGQISFGSPNAGSRSRDAERKKG
TPWTEDEHRLFLIGLDRYGKGDWRSISRNVVVTRTPTQVASHAQKYFLRHTSGKKERKRS
SIHDITTAMDSLPMPAPTNLPSHDGRGGFESCSFPMPR
>RMYB2
CREKFENVYKYHKRTKDGRAAKSDGKTYRFFDQLEALENAPPPNPFTPPRPAATASLSTM
PAHVTVSSISPTPLSVVPPQAAASIPIDSSPLIAQPPPQPPLMQHLRNSSVPPQGLNFQA
SRPGLMSDSTSSSTSSDEDIQRRRGRKRKWKDCFHRLVRDVIQKQEELQKKFLDSLEKRE
RERMAREEAWRVQEIARVNREHDLLVQERSIAAAKDAAVIAFLQKVTDQHNLQIPPVAAA
AEPPPPPPQQPEPPPAPPAPTTQPAPAPITVPPPAKVFNNSKTDNNGGEVAGYSPAASSS
RWPKAEVQALINLRTSMDLKYQDNGPKGPLWEEISSAMAKLGYNRSSKRCKEKWENINKY
FKKVKESNKKRPDDAKTCPYFHQLEAIYKERAKEVNNNNPMVPIMAQPEQQWPIPNLHRQ
QLPDSAMQDQDNRDNESDDHNINDEDDDDDDDEGLGFEILANKQHSSVANAAQES
>RMYB3
MESYNTPFENDQNVPLPASFPIHNICSSNPLSSNQDILHLDELALSSIIDRQSNPIDPFD
PFLNKYMPYEGNGYSSAVMQHIQGEGNQITDMNMQNFKGDGFLSFPNEVDSLNGIEAALN
YPDFMRLEYVNTDESSFVSADAFGCLKGNSTDTRKSCNADNSDGGSNVSKPVKGLWTLEE
DRLLVNLVKIYGAKRWTFMGQKLKGRTGKQCRERWRNHLQPDIKRDDWTEEEDKKLIEAH
AELGNRWAEIAKRLHGRTENTIKNHWNTTKRRKLTDGRCKNKSPIPTSALQDYIMSLDSK
KGSSSSNSNSNSNKSINSQA
>RMYB4
MGRPPCCDKLGVKKGPWTPEEDIMLVSYVQEHGPGNWRSVPPNTGLKRCSKSCRLRWTNY
LRPGIKRGTFTDHEEKMIIQLQALLGNKWAAIASYLPERTDNDIKNFWNTHLKKKLDRIQ
PGSGNRPTENGSSKPISRGQWERKLQTDIHTAKQALANALSVSTSESIDEDVMTNPNFAP
NPIQTTSYASSTENIARLLKDWANKTPKCEEFSNYNYSSCSTEEVVAGGADLGEGLFGFE
SFESYSSLSSDDFSRSGSPKPDPEGVLALEKWLLDDQGIQGLFDS
>RMYB5
MSITSESDERMAGSGVDSPSVDDANSGGTTTTGPNGPLKKGPWTSAEDAILIEYVNKHGE
GNWNAVQKHSGLARCGKSCRLRWANHLRPDLKKGAFTPEEERRIIELHATLGNKWARMAA
ELPGRTDNEIKNYWNTRIKRRQRQGLPLYP
>RMYB6
MGRAPCCSKVGLNRGAWSAMEDSLLTNFIQQHGEGQWRSLPNKAGLLRCGRSCRLRWMNY
LRPGIIRGNISEDEEDLILRLHRLLGNRWSLIAGRLPGRTDNEIKNYWNTHLHKKGAAAI
TLAIPPRELSKLESIEQKQNQDRKQKQKKILDPGGHNNDALTARKEKENDTPNKTRVYFP
KPIRVSSELSCTGSYNSLASGTSCGSDKAAEDVRREWPALLEIEDATFGVYNGDGEEAHF
LDGGECALIPVLTHSESISDDVEMLEKVYDEYLQLL
>RMYB7
MGRSPCCEKAHTNKGAWTKEEDDRLVAYIRAHGEGCWRSLPKAAGLLRCGKSCRLRWINY
LRPDLKRGNFTEEEDEVIIKLHSLLGNKWSLIAGRLPGRTDNEIKNYWNTHIRRKLLSRG
IDPTTHRPMNEAAADRETLKTTTTTTISFSGGCSRESEDQKIIVIRKDEDRRGATDQRCP
DLNLELRISLPCQDADGFATPGGSDTDSANSCFACRLGIRNSKDCRCGGGDGGGGRGGNS
GYDFMGLKTGLLDYRSLEMK
>RMYB8
MCVCLPKSTIPSCPNSEAKEANLSPIKRQNYWKSPWPLHQILHKMGRAPCCDKANVKKGP
WSPEEDAKMKDYIHKNGTGGNWITFPQKAGLKRCGKSCRLRWLNYLRPDIKHGDFSDDED
RVICSLFARIGSRWSIIAAQLPGRTDNDIKNYWNTKLKKKLMGMVNTHQAAAAACPLLPC
NNNNPQMPSSYECGYSNFYPCSQIRTLSTGVELEAMSPSFHPISNSSSNCNCNSLIYGGD

QPSCSSSEKSCFQGCNFGNFKGDGAEDHKLLISSSGCQVNYSVEEIKELISCNNGVGVDN
GSSFLMNGTNMMIK
>RMYB9
MASGKSIQLITYHEEIVDGEPIYVSSNSLPIKALNLEPAGHSFNAAALRLVGHCVEADTG
DDDDGKGAPNEKEHVYVESSDSYGSKGKKKSGEAKQQDHYALLGLSHLRYLATEDQIRKS
YREAALRHHPDKQAALLLAEETETAKQAKKDEIEGHFKAIQEAYEVLIDPLKRRIYDSTD
EFDDEIPYDCAPQDFFKVFGPAFVRNGRWSVTQPIPTLGDDKMPIKEVDSFYNFWYSFKS
WREFPHADEYDLEQAEGRDHRRWMERQNLKLSEKARKDESARIRTLVDNAYKRDPRILRR
KEEEKAEKQRKKEAKLMAKRLQEEETARIAEEERKRKEEEEKRAAEAALNQKKLKEKEKK
LLRKERTRLRTLSTPILSQRLLDLTDDDVESLCSSLDKDQLSKLCEKLERKEGLERAELL
KETLESNHKTKDKKEDEKNVLQNGSTSIHMNGQVSNKAEKPWGKEEIELLRKGMQKYPKG
TSRRWEVISEYIGSGRSVEEILKATKTVLLQKPDSAKAFDSFLEKRKPAQAIASPLSTRE
DGLGLSNAGAAMGQSSVPGNLSDSSNAVNGTALCSDQDSWSVAQEKGLVQALKTFPKETP
QRWERVAAAVPGKTVSQCKKKVALMKESYRNTSSSTVEKTPSNSNSNSDSNSNSNLSDLL
ESSTQNENPK
>RMYB10
MGRPSRDRKVGLRKGLWTREEDQKLLAYIQEHGHGSWRSLPAKAGLERIGKSCRLRWTNY
LRPDIKRGKFSSHEEKTIIRLHALLGNRWSAMAAYLPKRTDNEIKN
>RMYB11
MGRTPCCEKVGLKRGRWTAEEDEILTKYIEANGEGAWRSLPKNAGLLRCGKSCRLRWINY
LRSDLKRGNFTPQEEDAIVNLHASLGNRWSLIAGYLPGRMDNEIKNYWNSHLSRKIHSYF
RHFVPPPPPPKPNTMHRKRRQSLSSLEHLKIHGIDNNMVKSPAAVIATNVNHDVDVDDDP
CKKRTTNPDLKEQVIASSSSKPISTHDDHRQQGNNDQTLLQSGKLDDKEKTNTNTMTSAT
CLIDEAVGTKIHFMDDTTIKGSNGDLDENRGACNGNATENLVEHCCSTSSGYEYSSSSAP
SSSINIEDDDFWNLRSIESPIPWNFNSPFEEIWDHGKLDDDGWLGLLHDQNMAPLPWLWD
ASAFDDPEAHSVHD
>RMYB12
MMGHSIANDSDDGVLSADSPVSNDGDNFGGTSNGGIILKKGPWTSAEDAILVEYVKKHGE
GNWNAVQKHTGLLRCGKSCRLRWANHLRPNLKKGAFTSEEERLIIELHSRMGNKWARMAA
HLPGRTDNEIKNYWNTQ
>RMYB13
MAAEPDATTEEVSDKIKGPWSEDEDILLRELVKKHGARNWSQISKSITGRSGKSCRLRWC
NQLSPEVERRPFTPEEEEIIVRAHAELGNKWATIARMLNGRTDNAVKNYWNSNLKRKYEE
ARSAARAGVGERPPQVLRQSEIGDVSLAMWSQSEPGDYVNFFFPLIPVVKPTAAVNQAEA
GAAANGNSEEADRAVTALTLRLPGTGLSDSEKAYSSSNIPTEQRKSKDLPGRVSEVKEIT
SGELRTYIQETVREEMEKYFRRPGG
>RMYB14
CRLRWINYLRPDLKRGTFSQEEEDLIIELHSVLGNRWSQIAAQLPGRTDNEIKNLWNSSI
KKKLRQKGIDPNTHKPLQENINNSINDQLETSASAGNSKNNDKASEGSSCDLSFVDPDNN
ISNSVHGPVSGSGSALESPVVPLQVERYHPLLLENSTQELFLNRFVAAGCNHIAKPCVDL
SGFLSFQNPNYGPNIGLSMNNPDISTTTIPHQFTAFNNLSPPIVPQINKPPVNFPADNPS
LPPFTVKFESSTELQSGYSTFESGAGFPWTPADSSGKPDNKTTHGEIGEDMIKWNEYLQA
PFLMGNSIHNPTGQDHMYGETKAQGQFAADGSLSIGNWHTINPAPTFGQFS
>RMYB15
MKERQRWRPEEDTLLRAYVKQYGPRDWHLVSERMNQPLNRDAKSCLERWKNYLKPGIKKG
SLTEEEQRLVIRLQAKHGNKWKKIAAEVPGRTAKRLGKWWEVFKEKQQREQKENNKTIEP
IEEGKYDHILETFAEKLVREHIVPGITMPASNGGYLHGEQAAPSAPSVLPPWLASSSTTA
TVRPPSPSVTLSLSPLTVPPTPPIPWLQPPDRLADSATHGMMNNIHSLGIAPMVGESTVV
SELVECCKELEEGHRALASQKKEAAWRLKRVELQLESEKANRKREKMEDIEAKVKALREE
EKATLERIETEYREQLAGLRREAEAKEQKLAEQWAAKHIRLSKFLEQMGCRSVAAAEPNG
R

>RMYB16
MGRSPCCDKIGLKKGPWTPEEDSKLLAYIEEHGHGSWRALPAKAGKIFFSLFMCSFMSET
PVWGFFPGLQRCGKSCRLRWTNYLRPDIKRGKFSLQEEQTIIQLHALLGNRWSAIATHLP
KRTDNEIKNYWNTHLKKRLAKMGIDPVTHKPKNDALLSCDGQSKNAANLSHMAQWESARL
EAEARLARQSKLRSCSASPSFQDQCVEPAAGPTKPMGAPRCLDVLKS
>RMYB17
MGRSPCCDKIGLKKGPWTPEEDSKLLAYIEEHGHGSWRALPAKAGLQRCGKSCRLRWTNY
LRPDIKRGKFSLQEEQTIIQLHALLGNRWSAIATHLPKRTDNEIKNYWNTHLKKRLAKMG
IDPVTHKPKNDALLSCDGQSKNAANLSHMAQWESARLEAEARLARQSKLRSCSASPSFQV
QINDLNSIS
>RMYB18
MPFGSRYGDRHLEDDNFQPLGSRNDARYFRTNRENRGSFSQKDWRSTSWEPAATSSGPGR
FTTTEVNNQKSVENSETCHNNSKVNGSPNPLPDSTSDQSLSHTKEQEKNDGTADGGATSA
QKSEKENGLDSVDWKPLKWARSGSLPSRGPCFSHLSGTKSTGVDSIEVVAEAHQKSAMPV
QSPCTSNVRSSVPASSDETCCRKKPRLGWGEGLAKYEKKKVEGPEDGVVKDGRVLSVCIT
ENVHSPSVNLSDKSPRIASMSDCASPATPSSVAFSSSPGTEEKESMRPASVDQENNNLSG
LPCIMSQKHCEGPTFNLESIDLASIAKLSNFLNELLQSEDPSSAETDYVQTTSMKKLLVW
KVDVLKALEVTESKIDSLDTELKSLIAEPGSCCPCPDTSSLLQGECRLKPCMELVAASAS
TVDPTFQVASTGSMNVENMPASVEESEALKSEEINSPGCATYQIVEPSSGEDVCASGTAE
CEGFVNLDLKNSSNLNMLCLENGLSNEINSCQLDAAKPISDSCQKGNVGNVHCGGDSNYD
IIVASNKESAGRALEVLNKLLPSRNYFFDLSIASIMPPFRRSSSVKEKFLMRKRSLKFKE
KVITLKFRVFQHFWREGRIISVRKLRGKSNKMLDMTHCGHKKNRSSSRSRIPYFASNHRT
VPAEEVIKFIHGLLSESSFKPCRNTLKMPALILDKDVKMSRFISNNGLVEDPCAVEKERS
IINPWTAEEKEIFIDKLAVFGKDFSKIASFLEHKTIADCIEFYYKNQKSECFARARKKPE
FSEQRKSQATTYLVATGKRWNREVNAASLNVLGEATRGNNGLLQRSNSQEIYSNETVAAD
VLAGICGSLSSEAMNSCITSSFDHADGYQDWKCPRVNSGSKRPLTPDCTQNVDDECSDES
FEEMDSADWTDEEKSIFVQAVSSHGKDFDMIAQIVRTKSKEQCKIFFSKARKCLGLDQIH
HGAGNAVFNNANGGGSDVEDGCVVQAGSAVCNGDSECKMEEDLPPPNTKGKHEPDAVATH
NLKPDVHSLAAEPVLETSSVGDTLLDKKQAADSGAGAAGKGKSEGACGSELEGPTVVVSS
RTESLLVEEGSGSVVAVGTKETDKEEVPPDSAGSFCEENLGKGPLLSDDNFDAKKLEDRD
LNSSEITAISGAISEVKSDLQLAGNVSPVGAFSSMQVESGGQNLDNFHADKSCIISSPEN
GHMVSIESSSMMFSVPIKYQIHSNCNPLAYVGASRISEKHSLEAVGARDHQQNLSGYLLS
EAVDSPQILRGYPVSGPSVNEINGDVNCKRHAVQHLPERDLSLHSNQHPDFSLQKCTRHL
SEAASFQTPFQSHGKSSYDNSRPPSNLDKPARNGDVKLFGKVLISSQQKPNSCAQEACSD
KVEHHKASCQSLSLRCSTDHKSTFDSSVCGNKFIGSENISVRSYGFPDGNNTLMGFPSMP
DSTLVLAKYPAAFSSHSKPSGKLELPSLQGVRSSESPLNGVTAFQSSIDLSSSNADYEVL
KNQDLQFLTKEMKQQPQDIFSEMQRRNGFDPVQEIQHEPRMIGIGIGRGGMVVGGQCSGV
SDPVAAIKMHYAKAQNISVPASNEQREEDGSWKSNGDVGR
>RMYB19
MAHKIKEKQAGRKELNRGAWTANEDRKLVQAIGTHGAKQWPTIAAKAGLNRCAKSCRLRW
MNYLRPNIKRGNISDQEEDLIIRLHKLLGSRWSMIAARLPGRSDNEIKNYWNYHLSKKVL
KQGALGAPGISTGVKGCSGKIGEEKKQNGNGDGNRNRNVSSSNETEGSESLFDVN
>RMYB20
MADLGFHRSAKKCREKFENVYKYHKRTKDGRAAKSDGKTYRFFDQLEALENAPPPNPFTP
PRPAATASLPTMPAHVTVSSISPTPLSVVPPQAAASIPIDSSPLIAQPPPPPLMQHLRNS
SVPTQGLNFQASRPGLMSDSTSSSTSSDEDIQRRRGRKRKWKDCFHRLVRDVIQKQEELQ
KKFLDTLEKRERERMAREEAWRVQEIARVNREHDLLVQERSIAATKDAAVIAFLQKVTDQ
HNLQIPPVAAAEERPPPPPPPPQQPEPPPASPAPTTQPVPAPITVPPPAKVFNNSKTDNN
GGEVAGYSPAASSSRWPKAEVQALINLRTSMDLKYQENGPKGPLWEEISSAMAKLGYNRS
SKRCKEKWENINKYFKKVKESNKKRPEDAKTCPYFHQLEAIYKERAKEINNNNPMVPIMA
QPEQQWPIPNLHRQQLPDSAMQDQDNRDNESDDHNINDEDDDDDDDEGLGFEIIANKQHS

SVANAAQES
>RMYB21
MDTSGSSSSCSRALEVKASFKNAYSGSSRTWGRDKAFENAVALCHNDADPWKQFPVAVPG
KTIVDVKHHYKALRYDVEAIGSGKVPLPHYPSNIMVCEKKEIPLRIGGKQRIHWKDEEHG
GSSWTEEEDKAFDNALAFFRGDPDLWDKVVLAVPGKSIEELKLHYEALIVESRKVPLPHH
FSKNKAKKEKPLGIHEQKRFRWTEEEHRSFLRGLEKFGKGDWKSISSYCVPTKTNTQVAS
HAQKYFTGLSTARRNQKRS
>RMYB22
VENTQTYSKVNNHKAMGTTQTLHENNSVGSAHPSESLSSPSQPRLKEKHEKVAAADTLTA
LGQNSDKEDGLGSIDLKPLNCSRSGSVESRGEVNNQVIENTGTCSKVSNQKAVGNTQTWH
ENNTVSSASPLPKSLSSPSQPCPKEKHEKVGAADTLTSLTQNSDKENGLGSIDWKSLKWS
RSGSLVSRGSGFSHSSSSKSLGVDSTEIVADVQQKDVMRVQSPGAACAMPNAPDPSDETS
SRKKPRLGWGEGLAKYEMKKVDGREDGAAKDRLVVSVTSTEAMQSPSVSLADKSPRFANL
MECVSPATPASVGCSSSPGITSIDDKESIKAANVDQNTANMSCSPDVTSQIYHGGPAFIL
ENLQLTSVANLSSLINEFQNSNDPSSAKTGYIRTTSMNKLLLCKLDILKALEVTESEIDS
LETELKSLTAESDGCHPSEDHAAASGITVVPALLHIALSGNMNSGDTPGALKDQNLEQKD
EEIDSPGTATSKLVEALSHGEDTCTSKTTEFAEGFVKLELDNASYLNETLLKNGLCDDDN
VCPVDIPNSTMSNWQHLDGVGDIQCDHIYDSIIASNKESANRAVEELNKLLPDKKCIFDI
STVTSVSSSQRDFLKIKDRFLAKMLSVKFKEKIIALKFRVFQHFWKEGRVVSARKLGGKS
HKMFDLSRTGYKRKCSFIRSRISYFAGSHQIVPAEEIVEFINGLLSISAIKPCRNFLRMP
GFILKEKRMSRFISNNGLVDDPCASEKERSIINPWMAEEREIFIDKLAIFGKSFSKIAAF
LEHKTIADCIEFYYKNHKSECFERARKKPEFAKQSKSKASTYLVASRKRLNREANSVNLD
MLGDASVIVAAVNDGLGTQQQPKCTSRILFGAASSYKIPRGDDGPLQRSNSLDLDSNETA
AADVLAGICGSLSSEAMSSCITSSFDPRASSSIRWPLTPDVTQNVDDECSDESCGEIETV
DWTDEEKSIFIKSMSSYGKDFARISQCVGTKTKEQCSIFFSKARKCLGLDRIHPGTGHVN
AGGSDIEDGCAVQIGSVICSDDSANKIEENLTLHEMKMDQASDTAESHKLKPDASDYGEG
SRACPLDSMAAGAVLRNSSVSNSLVDDKSALDLNSGSKEEIGADGSELDVRTMFVSNSNS
PQVELGFDCGLPDGLSKAYNKASVKVSGGHVQGDNQQEGHNLPEENMNSKKVECRDANLV
DMAFSSAVNELK
>RMYB23
WDKVSRKLGELGYNRSAKKCKEKFENIYKYHKRTKDGRSSRQNGKAYRFFEQLELLDAQF
SVPSTPSNPLPSYTRPASDPAAAEAAMAKQGGCSQDFPIPCSNYPIAEFTSASTSTASSS
GRDSEGSIKKKRKLVDYLERLMKDVLVKQEDLQNKFLEALEKCEKERMAREEAWKAQEMA
RIKREQEYLAQERAIAAEKDAAVLAFLQKITQQALPLQMPEVLASLFDKRSENQESVAEK
RAFVQDNGIGEASAHNEKQDNSAGENVVQNSSSRWPKAEVEALIMLKTDLDMQYQDSGPK
GPLWEEISARMKKIGYDRSAKRCKEKWENINKYYKRVKDSNRKRPDDSKTCPYFNMLESL
YAKRAKKSDHNSENGTSSLKPEQILMQMMGQEQHQQQPEQTIGEYGDNDHQNQGEGMDDE
EDRESGDGYQVVANNLSSVTTLG
>RMYB24
MAAAEASGGLDRIKGPWSQEEDELLRKLVERHGGRNWTLVSRSIPGRSGKSCRLRWCNQL
SPEVEHRPFTAEEDERILQAHRNFGNKWAKIARLLNGRTDNAVKNHWNSKLKRKAAAAAA
SGGDERSAKVWRRAENGDVSAALRVSPESMNLSNSETNDSSEDNVNFYFPVARSSPGVES
PQYGAAENEDNSNIELTLLTLSLPGSVSSSAKEISEKDSPSSDTPPRRGGNGAFPPPGPA
FDPKLLQIIQEMIRSEVRNYFSREMNGGDGILSGKRIAISDFNE
>RMYB25
MKKGNNKSGGANSGDFPKRKEWHIVSWSQEEDDILREQIRLHGIENWAVIASKFKDKTAR
QCRRRWFTYLNSDFKKGGWSPEEDMLLCEAQKIFGNRWTEIAKVVSGRTDNAVKNRFATL
CKRKAKHEALAKENNFTSYINLNNKRVIFPNGLNSGGYKSGAPLKKMRNDVTNAVESQEG
RPSSECDAVDHLLRHPFQVLAQNAHSSAVSIPSELHSNQIKEALMDGLEDCKIEGTFLRK
DDPKVLALMQQAELLSSLAIKVNSEKTNQSLESAWIAIQEFLKQNRENNLPAFENPDHDF
HLEKLRELVEDLRSSNEGSRLSWREPDVYASSPASSEYSTGSTLLPNIPADKMEEHKTDI

SVLHQELGNEFVEESRICNTTASTNEVNMLTCGHIEENCEEVCDYSNTEFGSPVHVTPLF
RTLAAAIPSPKFSESVRILSF
>RMYB26
MSMPSGSNKRMVSSEVLPPSRDNASSGGGATTGEANILLKKGPWTSAEDAILIDYVTRHG
EGNWNAVQKHSGLARCGKSCRLRWVNHLRPDLRKGSFTQEEERKVIELHSRLGNKWARMA
AELPGRTDNEIKNFWNTRTKRLERSGLPPYPPDVRLRMAKENQLNNSSKNTFLFNTVPQQ
GFNFASNPEIPNFKALELNSPIYSTHPAPMPNILPGSLLDISARSLVAQGRNLNSSNINK
PHQSTVYPSKRLRPSEPPLQGFNTATGNALASGSPFDDPLQIGQPLVFSSGHDCYPTCNR
IMSSSLLDGSVNHAVLYGNPASEPAQATKLELPSLQTQTGHWVLPPPP
>RMYB27
MRSPSSSSSKKTSSANGDGCSSGKQAAVTPYCSNVGLKRGPWTAEEDELLAQYIKREGEG
RWRTLPKKAGLLRCGKSCRLRWMNYLRPSVKRGHITPDEEDLILRLHRLLGNRWSLIAGR
IPGRTDNEIKNYWNTHLCKKLIRQGLDPKTHKPFNPNKSSVNVNRDSSSEALEHKSSVSS
PIPNNSTTFNPFENVITNSDQLEGGHNMDYPISNDEELRNCNKRIIIRGDEDEDDDDDLL
NCCIDDSFLNSLINDDIFNEEQQ
>RMYB28
MPSASELSSSVWSREQDKQFEKAIATYPEDTLDRWEKIAADVPGKSVEEVKQHYEVLVDD
VQRIESGFVEVPCYNSASDGSTSHAGDGTPSRKGANSGQSNGESNHGGKSSKSDQERRKG
IAWTEDEHRLFLLGLDKYGKGDWRSISRNYVVTRTPTQVASHAQKYFIRLNSMNKDRRRS
SIHDITSVASGDISLPQGPITGQANGSSAGGPSGKSSKPLPQAPVATAGVSMYMPPPTIG
QPIGGPLVSAVGTPVNLPSPMHMAYGVGAPVHGSVVPGVGLSMPPMTYPGPHPSAHR
>RMYB29
LLLVFINSAGKMRIMIKGGVWKNTEDEILKAAVMKYGKNQWARISSLLVRKSAKQCKARW
YEWLDPSIKKTEWTREEDEKLLHLAKLMPTQWRTIAPIVGRTPSQCLERYEKLLDAA
>RMYB30
MKKKVNNSTKASNSGVPKQKERHIVSWSQEEDDILREQIRVHGIENWAIIASNFNDKTTR
QCRRRWFTYLNSDFKKGGWSPEEDMLLCEAQKIFGNRWTEIAKVVSGRTDNAVKNRFTTL
CKRRAKHEALAEENNTASYINSNNKRVIFSGGSETAVPVKKIRTYISNTAAENQEEKVAG
ECDATDDLHRHPFATVRQIARVHEFDFPAQMNVNHIKGASTDGSEEKNKSAGTFLKKDDP
KVIALMQQAELLSSLAIKVNSQKTDQSLESAWMVLQNFQKQNKESDLIMFTHPDIDLQFE
NCNELVEDLRSSNEGSESSWREPHMCESSPGSSDYSTSSTLLSSVPGDKIEACQSEISVL
HWETGKELQSNPVDDQCCLSVESEMCHTATANEVNMVSGNDTKENNEAACGYSNTEFSSP
LHVTPLFRALAAAIPSPKFSESEKHFLMKTLGMESAAPSTMTNPSQPPPCKRSLLHCL
>RMYB31
RYGPHNWNAIAEKLQGRSGKSCRLRWFNQLDPRINRSPFSEEEEERLLACHRIHGNRWAV
IARFFPGRTDNAVKNHWHVIMARKCRERSKFYAKRTLSHHYHRRHQKNQPIIGRSGINAH
EKEALSLTLSSSSSSSKCDNNSRVLAAPTNCTSTARKDLGINNNYTTLGLGHTFLDGFAH
QQISNPLSMYNLPSALMLPLWNDRQHVKIAQENGSREVEFYNFLQVNTDSSNRSEVINNH
KHAKRNIEEEEHQEEEEEVDQKGSIGETKNTAAAGGVVALPFIDFLSVGRAGN
>RMYB32
MPADESSSSVWSREQDKKFEKAVATYPEDSSDRWEKIAAQVPGKSVEDIKYHYQLLVDDV
NQIESGFIQVPCYNSSSDGSTSHADDEGAGKKSGNSGLLNNDSNHGGKSSRSEQERRKGI
AWTEDEHRLFLLGLEKYGKGDWRSISRNFVVTRTPTQVASHAQKYFIRLNSMNKDRRRSS
IHDITSVNNGDVSVPQGPITGQTNSMAVGGGPSPKSNKQSLQVPVGVNMYGTPTIGQPIG
GPLVSALGTPVSLPPPPHLATYGVGAPVVPGAPTTYPMPHTSAHR
>RMYB33
TGLSRCSKSCRLRWTNYLRPGIKRGNFSEHEEKMIIHLQALLGNRWAAIASYLPHRTDND
VKNYWNTHLRKKLGKQQQQPQGHVYVDGNNNSSRSKPVSVTKGQWERRLQTDIQTAKQAL
SEALSLDKSDSAAVVDQHPSPTTQTRPIQTSMYAVNADNIARLLENWKSNDSPKLHQGA
>RMYB34
MSMPSGSNKRMVNSGVLPPSRDIASSGGVATTGEANILLKKGPWTSAEDAILIDYVTRHG

EGNWNAVQKHSGLARCGKSCRLRWVNHLRPDLKKGSFTQEEERKVIELHSRLGNKWARMA
AVLPGRTDNEIKNFWNTRTKRLERSGLPPYPPDVRLRMAKENQLNSSSKNTFLFNTVAQQ
GFNFACNPEIPNFKALELNSPIYSTHPAPMPNIHPGSLLDISARSLVAQGRNLNSSNINK
PHQSTVYPSKRLRPS
>RMYB35
GPPNPNPMADKNRHHLPHDHDGNGGDDDDNDDDDDDGALPRKRSSPSSSSSLNRGAWTPE
EDATLAKFIEIHGPRRWKSVAMKSGLNRCGKSCRLRWLNYLRPDIKRGNISEAEDDLILR
LHRLLGNRWSLIARRLPGRTDNEIKNYWNAHLRKKAKLMEKLPSTSTVALIKKWDEDNDD
DDDDDDDDQEREKASSSMEIASGSEMVTKRMSVGDGMGSSDVEGDELFDFSRQGTCGLEW
VKSFLELEEDA
>RMYB36
MGRTPCCAKNGLKKGPWTQEEDQKLIDYIQKHGYGNWRTLPKNAGLQRCGKSCRLRWTNY
LRPDIKRGRFSFEEEETIIQLHSILGNKWSAIAARLPGRTDNEIKNYWNTHIRKRLLRMG
IDP
>RMYB37
MGRPPCCDKIGVKKGPWTPEEDIVLVSYIQEHGPGNWRAVPANTGLLRCSKSCRLRWTNY
LRPGIKRGNFTDHEEKMIIHLQALLGNRWAAIASYLPQRTDNDIKNYWNTHLKKKLEKGH
EKSGQSG
>RMYB38
MDMEKDELRKGPWTEQEDAQLVLYVNLFGDRRWDFIAKVSGLRRSGKSCRLRWVNYLHPG
LRRGKMTPHEERLVLQLHAKWGNRWSKIAQKLPGRTDNEIKNYWKTHMRKKAQEEK
>RMYB39
MKERQRWRPEEDTLLRAYVKQYGPRDWHLVSERMNQPLNRDAKSCLERWKNYLKPGIKKG
SLTEEEQRLVIRLQAKHGNKWKKIAAEVPGRTAKRLGKWWEVFKEKQQREQKENNKTIEP
IEEGKYDHILETFAEKLVREHIVPGITMPASNGGYLHGEQTAPSAPSVLPPWLASSSTTA
TVRPPSPSVTLSLSPLTVPPTPPIPWLQPPDRLADSATHGMMNNIHSLGITPMVGESTVA
SELVECCKELEEGHRALAAQKKEAAWRLKRVELQLESEKANRRREKMEDIEAKVKALREE
EKATLERIETEYREQLASLRREAEAKEQKLAEQWAAKHIRLTKFLEQMGCRSVAAAEPNG
R
>RMYB40
MKERQRWRPEEDTLLRAYVKQYGPRDWHPVSERMNQPLNRDAKSCLERWKNYLKPGIKKG
SLTEEEQRLVIRLQAKHGNKWKKIAAEVPGRTAKRLGKWWEVFKEKQQREQKENNKTIEP
IEEGKYDHILETFAEEL
>RMYB41
MSNMERIKGPWSPEEDQLLQKLVEKHGPRNWSLICKSIPGRSGKSCRLRWCNQLSPQVEH
RAFTPEEDETIIRAHAKFGNKWATIARLLSGRTDNAIKNHWNSTLKRKCVSMSEEDLNAA
FDPDAQPPLKRSASVGPGTEVFCFNPGSPPSSGSDVSGVSHSGHLVFRPIARAGGFSPPD
LQLPDPVTSLSLSPPGSDLNPNPNTNTVTEQLNQSPPRFNPNPNAVDPAVAVGMTSPAMM
QFDSAVSNQFFSPDFLAVMMEMVRKEVRNYMEGIEQNGFGVQNDAIRNDAVVK
>RMYB42
MAAKAKKVAPEAVPERVKGPWSPEEDELLTKLVQQHGARNWTLISQSIPGRSGKSCRLRW
CNQLAPEVEHRPFTEEEDEIIVQAHAVHGNKWATIAKMLTGRTDNAVKNHWNSTLKRKSY
SGRSDGGSARLPQALRRLDNRDVSAAIIYESESSCLMDSDSRDSDHKKNIPADPPTPPVK
DNVDLTELSLSLPGFARNSAPLEINLSNIPNFEQGRASKTSLFSPELATMVQQIIREEVR
NYMSQHSLKMHSLAL
>RMYB43
GLNIGGGDGGAGRWPRQETLTLLEIRSRLDHKFKEANQKGPLWDEVSRIMSEEHGYQRSG
KKCREKFENLYKYYKKTKEGKAGRQDGKHYRFFRQLEALYGDSNGAASASDQANLVGGNF
HYNYGNKSTSLGANQESLQGQKLSDTSLSLSNYSDEVDSSSSDDTDLNEGNDKADDPSNK
KRGKRGWKVKIRDFIDLQMKKLMDKQEVWMEKMMKTMEQKEHERMVREEEWRRQDIVRME
KEQKFWASERAWIEARDAALMEALHKLTGKDNHLLGAENQIQNNNEDIGSETMTNSAKGG

DMIWPESEISRLIQLRSGMEAKFQQRGVSSEEVLWEEIATKMACYGHDRTGVLCKDKWDS
VNNYLLKCNKKRKENSKGCTTAYYHEQVSNQGPGGHHVYSCNDASHEQQHQQQQHAAVNS
SPNSNTGNPMTTDSC
>RMYB44
MGRSPCCEKAHTNKGAWTKEEDQRLINYIRAHGEGCWRSLPKAAGLLRCGKSCRLRWINY
LRPDLKRGNFTEEEDEIIIKLHSLLGNKWSLIAGRLPGRTDNEIKNYWNTHIKRKLISRG
IDPQTHRPFNSTAPAASTGGAPALPAKACLDFRRSTPPQAATSMNEATNNIRSSPAIDNS
SSGDNTKCSSTTTEESQPPPPVKMEVENDPYAVDLELSLGLPSPSKTKYAKSGSFNSSSS
VESKPFSEFLRGAGKPSTAAEPMCFRWPLSFQGSVI
>RMYB45
ISTVLIINSHSDYCYCLFHGFYKTPFFTPFLCLIIFFRNFATRVFPYSPILNWAEQNLRC
TSVSPLKKGLILDEMETLCQTSLASDSNWFIHQGMTTVWTKEENKQFESALAIFDEKTPN
RWFKVAAMIPGKSVYDVINQYRELVADVSDIEAGLFPIPAYLASSFTYELADLRSFDVCR
KRVRTCDQERKKGVPWTEDEHRRFLLGLQKHGKGDWRNISRNFVISKTPTQVASHAQKYY
LRQLSGAKDKRRPSIHDITTIHLTNTSSSENNKPPLLEKPSVQHTSVQKPTYAPKVFVDR
SHINDKTLMDLDSAHGDLLVAYPCNVAAQTWHNVKTGASHPHFRI
>RMYB46
MPSDESSSSVWSREQDKQFEKAIATYPEDGLDRWEKIAADVPGKSVVEVKHHYELLVDDV
NRIESGVVEVPCYSSSSDGSTSNGGDEASGKKSSNSGRLNGDSSHGGKSSKSDQERRKGI
AWTEDEHRLFLLGLDKYGKGDWRSISRNYVVTRTPTQVASHAQKYFIRLNSINKDRRRSS
IHDITSVNNGDVSVPQTPITGQTNSAAAGGPPMKSNKPLPQTAVGTPGIGMYGPPTIGQP
IGGPLVSAVGTPVNLPPPHLAYSVGAPIPGPPVVPGTPLTMAPITYPVPHK
>RMYB47
MADKNRHHLPHDHDGNGGDDDDNDDDDDGDGALSRKRSSPSSSSSLNKGAWTPEEDATLA
KFIEIHGPRRWKSVAMKSGLNRCGKSCRLRWLNYLRPDIKRGNISEAEDDLILRLHRLLG
NRWSLIARRLPGRTDNEIKNYWNAHLRKKARLMEKLPS
>RMYB48
KGPFWEEVSRKMASLGFQRSSKKCKEKFENVYKYHKRTKDGRAAKSDGKSYRFFEQLEAL
ENSPAPPRSAAPAAPAPPPNLQMPSHVTVSSISPTPLSVIHPITNPQLNSPQFQQSQPPQ
FIQHQHTIAATNSQITPQARIFRSESTTSSSSTSSDEDIQRRRGKKRKWKDYFERLMKEV
VEKQEALQIKFLETLENREKDRTAREEAWRVQETARMNREHEILVRERSMAAAKDAALIA
FLQKVTDQNNPQIPPVTPAPAKPPPPPPPQQPQQSNPPTTKAVETSKTDNGGGGESVLPG
SSSRWPKAEIQALIDIRTSLDFKYQDNAPKGPLWEEVSAAMAKLGYNRNAKRCKEKWENI
NKYYKKVKESNKKRPEDSKTCPYFYQLEAIYKERAKHGIVSSMNPSYPNNPNMEPILVRP
EQQWPLPNNNQQQQQKDSTFRDQNHNENESENNEHEEDEFDEEEDEMDENGGEYELIAN
KQSSTMG
>RMYB49
MNREIGQVSPASYFTNSNWLFEGSTVAKWTPEENKRFENALALFDKDTPDRWQNVASMIP
GKTVNDVMKQYRELVEDVSDIEAGLIPIPGYSNNSFTLEWVTNHGYDGLKQIYCPGGKRG
SSTRCSDHERKKGVPWTEEEHRQFLLGLKKYGKGDWRNISRNFVTTRTPTQVASHAQKYF
IRQLSGGKDKRRSSIHDITTVNLDETNKSPSPEDKDASHSPQKSNMILQSHPNLGGNVVV
RATYDSNLTNPGAMMGFTPQDGGLMITGLQGTSSFGVNLNEHHMQGFDQRGLWLGHPGTI
FQMHPTRHS
>RMYB50
GKSCRLRWINYLRPDLKRGNFTEEEDEVIIKLHSLLGNKWSLIAGRLPGRTDNEIKNYWN
THIRRKLLSRGIDPTTHRPMNEAAADRETLKTTTTTTISFSGGCSRESEDQKIIVIRKDE
>RMYB51
MVRAPCCEKMGLKKGPWSPEEDQILVTYIQQHGHGNWRALPKQAGLLRCGKSCRLRWTNY
LRPDIKRGNFTKEEEETIFTLHQLLGNRWSAIAARVPGRTDNEIKNIWHTHLKKKLENNS
NINPSP
>RMYB52

MASGKSIQLITYHEEIVDGKPIYVSSNSLPVKALKLEPAGHSFNAAALRLVGHCVEADTC
GDDDGKGAPNEKEHVYVESSDSYGNKGKKKSGEAKQQDHYALLGLSHLRYLATEDQIRKS
YREAALRHHPDKQAALLLAEETETAKQAKKDEIEGHFKAIQEAYEVLIDPLKRRIYDSTD
EFDDEIPCDCAPQDFFKVFGPAFVRNGRWSVTQPIPTLGDDKTPIKEVDSFYNFWYSFKS
WREFPHADEYDLEQAEGRDHRRWMERQNLKLSEKARKDESARIRTLVDNAYKRDPRILRR
KEEEKAEKQRKKEAKLMAKRLQEEETARIAEEERKRKEEEEKRAAEAALNQKKLKEKEKK
LLRKERTRLRTLSTPILSQRLLDLTDDDVESLCSSLDKDQLSKLCEKLERKEGLERAELL
KETLESNHKTKDKKEDEKNVLQNGSTSIHMNGQVSNKAEKPWGKEEIELLRKGMQKYPKG
TSRRWEVISEYIGSGRSVEEILKATKTVLLQKPDSAKAFDSFLEKRKPAQAIASPLSTRE
DGLGLSNTGAAMGQSSVPGNLSDSSNAVNGTALCSDQDSWSVAQEKGLVQALKTFPKETP
QRWERVAAAVPGKTVSQCKKKVGLMKESYRNTSSSTAEKTPSNSNSDSNSNSNSNLSDPLES
STQNE
>RMYB53
MASGKSIQLITYHEEIVDGEPIYVSSNSLPIKALNLEPAGHSFNAAALRLVGHCVEADTG
DDDDGKGAPNEKEHVYVESSDSYGSKGKKKSGEAKQQDHYALLGLSHLRYLATEDQIRKS
YREAALRHHPDKQAALLLAEETETAKQAKKDEIEGHFKAIQEAYEVLIDPLKRRIYDSTD
EFDDEIPYDCAPQDFFKVFGPAFVRNGRWSVTQPIPTLGDEKTPIKEVDSFYNFWYSFKS
WREFPHADEYDLEQAEGRDHRRWMERQNLKLSEKARKDESARIRTLVDNAYKRDPRILRR
KEEEKAEKQRKKEAKLMAKRLQEEATARMAEEERKRKEEEEKRAAEAALNQKKLKEKEKK
LLRKERTRLRTLSTPILSQHLLNLTDDDVESLCSSLDKDQLSKLCEKLERKEGLERAELL
KETLESNHKTKDKKEDEKNVPQNGSTGIHMNGQVSNKAEKPWGKEEIELLRKGMQKYPKG
TSRRWEVISEYIGSGRSVEEILKATKTVLLQKPDSAKAFDSFLEKRKPAQAIASPLSTRE
DGLGLSNASAAMGQSSVPGNLSDSSNAVNGTALCSDQDSWSVAQEKGLVQALKTFPKETP
QRWERVAAAVPGKTVSQCKKKVALMKESYRNTSSSTVEKTPSNSNSNSNSDSNSNSNLSD
LLESSTQNENPK
>RMYB54
MDTSGSSSSCSRALEVEASFKNACSGSSQTWGRDKAFENAVALCHNDADPWKNFTVVVPG
KTIEDVKHHYKALRYDVEAIGSGKVPLPHYPSNIMVCEKKGMPLRIGGKQGIHWKDEEHG
GWNWTEEEDEAFDNALAFFHGDPDLWDKVLLAVPGKSIEELKLHYEALIVKSRKVPLPHH
FSKNKAKKEKPLGIHKQKRFRWTEEEHRSFLCGLEMFGKGDWKSISSYCVPTKTNTQVAS
HAQKYFTGLSTARRNQKQSNIKDTPTAHLGSMPSFPTGIMPGNPISAGKHPLSPKPLGIT
PDTPFSTQLISKQGISAKGRPTYLDPFWQDDAILHPGSFHFTT
>RMYB55
MRKSSGKKKEESSGGGRGGGGGREAWTKEEDQMLIDYIKLHGEGCWTSLPNAAGLQRSGK
SCRLRWVNYLRPDIKRGNFGHDEDDLIIKLHALLGNRWSLIARRLPGRTDNEIKNYWNSH
LKRELIAMGIDPNNHRIHQTMFATTRPAAACSNVDTANPSSDNKMLSSSKTCLKAVSETS
TTSSLPPPQVNLDLSIAFAAPSGKEAAAGRGSRNEP
>RMYB56
MASLGFQRSSKKCKEKFENVYKYHKRTKDGRAAKSDGKSYRFFEQLDALENYPAPPRSAA
PAAPAPPPNLQMPSHVTVSSISPTPLSVIHPITNPQLNSPQFQQSQPPQFIQHQHTIAAT
NSQITPQARIFRSESTTSSSSTSSDEDIQRRRGKKRKWKDYFERLMKEVVEKQEALQIKF
LETLENREKDRTAREEAWRVQETARMNREHEILVRDRSMAAAKDAALIAFLQKVTDQNNP
QIPPVTPAPAKPPPPPPPQQPQQSNPPTTKAVETSKTDNGGGGDSVLPGSSSRWPKAEIQ
ALIDIRTSLDFKYQDNAPKGPLWEEVSAAMAKLGYNRNAKRCKEKWENINKYYKKVKESN
KKRPEDSKTCPYFYQLEAIYKERAKHGIVSSMNPSYPNNPNMGPILVRPEQQWPLPNNNQ
QQQQQKDSTFRDQNHNENESENNEHEEDEFDEEEDEMDENGGEYELIANKQSSTMG
>RMYB57
MGHQSCCVKQKLRKGLWSPEEDEKLFNHITRHGVGCWSSVPKLAGLQRCGKSCRLRWINY
LRPDLKRGMFSQEEEDLILSLHESLGNRWAQIATRLPGRTDNEIKNYWNSSLKKKLMNEG
IDPNTH
>RMYB58

MAELGFQRNAKKCKEKFENVYKYHKRTKDGRSSKSDGKTYRFFDQLKALENTPPPPPPPP
ASMAAPPLPTLLPKSTVPSSISPNPVSMAPPSALQPPPPPLAPVNISNPADTHALRRPPP
PPNLFSNTMTSSSSTSSDEDIQQRRSGSGRKRKWKVFFQRLMKDVIQKQEELQKMCIETL
EKRERDRTARDEAWRMQERARLNREHELLVQERSMAAAKDAAVIAFLQKVTDQQNLQIPI
SLPITQKIPQPPPAPPPPIKSFDKIGDNSMPGSSSRWPKAEIEALIKLRTNLDLKYQETG
PKGPLWEEISAAMAKLGYNRSSKRCKEKWENINKYFKKVKESSKKRSEDSKTCPYFHQLD
AIYKVRGKSDTKPENPPMMTRPEQQWPLPAVQPPPPQDDAAMDLDRDDDDDDDDDDGDD
DEEDQGYEMVGDKQPAERG
>RMYB59
MFDGIGIMCSSDHHDYLSQFINASRTSSLLDDHIHCNPCFDPSLISSSLDNSITQGERSV
VPWSNEEVVELFKFRSSMESCTSLPELISWGHVSRRLAEIGFKRSAVECREKFEGKNFNN
VISCKDSRVCNELDESYLHDHPEEIQVSAECGPAAETKDVPVVYDQERADKDVAKVSENN
EAAKKASQQLTREGKSKRKRKEGEVGALRGYFEAIVDKVMARQEELHNELIASIIKRDEE
RMAREEEWKIQEIKRSKTAMQMRAKDQANAEERQATIIQFLKIFQDQHINANKVIQTGSS
PSRLLIPSLKTNPISSRWPREEVLALINMRCKVSYNSKEGQMKGPLWERISQGMMELGYK
RSAKRCKEKWENINKYFRKTKDR
>RMYB60
MAAAITSGGGGGSGEFCKKMDLDRIKGPWSPEEDELLQQLVQKHGPRNWSLISKSIPGR
SGKSCRLRWCNQLSPQVEHRAFTPEEDETIMRAHAKFGNKWATIARLLSGRTDNAIKNHW
NSTLKRKCSSFSSDEAFFDSADQPPDRPLKRSVSAGSGVPGSGLYLAPGSPSGSDASDSS
LPVMSGSFVYKPVARSGALLPVQIEPVVNDPPTSLSLSLPGAETIESPNLKIEAVQAKPN
IPPPPPPPPVTPPAAIPIAIQPQSPVSKVPSPPPVQDKVFAPFSAEMMAVMQEMIRTEVR
NYMMGLEQQRFQHQHQHQQNQQHQQQYIQLQIQQQHYNQQQQQQQYMCMQQANINEQFRN
AAMKRIGINRIE
>RMYB61
MDNKKACRRSEEVEVRKGPWTMEEDLILMNYIANHGEGVWNTLARSAGLKRTGKSCRLRW
LNYLRPDVKRGNITPEEQLLIMDLHAKWGNRWSKIAKYLPGRTDNEIKNFWRTRIQKHIK
QPANESSSDYQQQQQQTNNSNMANHGNTSQVSLGYGPVDHVVDQMSYSSPACSFTPGTME
HFQAPAFPTPDSNENLWSMEDLWSMHLLHGD
>RMYB62
MRIMIKGGVWKNTEDEILKAAVMKYGKNQWARISSLLVRKSAKQCKARWYEWLDPSIKKT
EWTREEDEKLLHLAKLMPTQWRTIAPIVGRTPSQCLERYEKLLDAACAKDENYEPGDDPR
KLRPGEIDPNPESKPARPDPVDMDEDEKEMLSEARARLANTRGKKAKRKAREKQLEEARR
LASLQKRRELKAAGIDVRHRKRKRRGIDYNAEIPFEKRPPPGFYDVTEEDRPTELVKFPT
TIEELEGERRVDREARLRKQDIAKNKIAQRQDTPAAIMQANKLNDPETVRKRSKLNLPAP
QIPDHELDAIAKMGIASDLVGNEELTEGNAATRALLASYAQTPRQGMTPMRTPQRTPAGK
QDAIMMEAENQRRLTLSQTPLLGGDNPMLHPSDFSGVTPKKKDIATPNPLLTPSATPGGQ
GLTPRIGTTPSRDAYSLSMTPKGTPMRDELHINEDMDVHDSDKLRQADSKKELLSGLKNL
PQPKNEYQIVIQPFAEDEEEPEEKIEEDMSDRTAREKTEEEARLQALLKKRSKVLQRELP
RPPTASLDLIRNSLIRADEDKSSFVPPTLIEQADELIRKELLSLLEHDNTKYPLDEKVAK
EKKKGSKRSAKAKSAPVPSIDDFEENELKEADELIKNESDVLRVAMGHEDESLDEYVEAH
ETCLNDMMYFPTRDGYGLSSVANNMEKIAALQNEFDKVKKEMDNETKKAQKLEQRIKVLT
NGYQMRAGKLSTQIEATFKQMDTAGTELECFQVLHKQEQLAATHRIGNLWEEVQKQKDLE
NTLQKRYGDLMSELERVQNLVGAYRLQAEKEAENAAKKDDDPQVNEINTLDESVAPNDGT
PNDEVLVLDEAANAATNQSIAPDLETSNHEVAAENNDVVPVEPEAALDQSVSSDAGNPEP
AVISDERTSMEVDNIDETRSQQLDVADVHIQPITEHGRDGGASVTGDSFELDSAMDG
>RMYB63
MTSRNKTYAEGRKRMKPYCKCHWTAEEDALLTTFVQAFGAGGWDFIAHRIEGRTAKSCRL
RWVNHLSPNVCKAPFTEEEQLRLLHLQKEVGNRWSLIVKHFRGRTDNQLKNKFNVLMRSR
KRTMGTSSGSDSHVPLKGGPTGNYYPGFTPYTSTPMNAQASPLISGGSGSGHGKGGTVNK
LSRPIGANRLNIYYCHMTSHGNPIMSDANSVVSHGICLPVAQPQQGAGHKEPQFFDFMGV

GTSE
>RMYB64
CDKVGLKKGPWTCDEDKKLIAFMLTNGSQCCWRAVPKLAGLLRCGKSCRLRWTNYLRPDL
KRGLLSQYEEKMVIDLHAQLGNRWSKIASHLPGRTDNEIKNHWNTHIKKKLRKMGIDPLT
HKPLPPPTTTDQLPPQPTELADDQSPNLNEDKTT
>RMYB65
MGRPVAPCCGRIGVKKGPWTPEEDIILVSYIHEHGPGNWRSVPSNTGLSRCSKSCRLRWT
NYLRPGIKRGNFSEHEEKMIIHLQALLGNRWAAIASYLPHRTDNDVKNYWNTHLRKKLGK
QQQQQTQGHVCVDGNNNSSRSKQVAVSKGQWERRLQTDIQTAKQALSEALSFDKSDSAAV
VDQHPSPTTQTRPIQTSMYAVNADNIARLLENWKSNDSPKLHQAAGSSESCNNNLQSPSE
VGFDSSNLGMTVMPLSLLEKWLFDESGAQGQDGDFMGIIE
>RMYB66
IPLAIPLSERKKRFLTSCKKIWGMEMLNRCSSGSSSCSSDSSTVSGRKSERIKGPWSAEE
DNILTRLVEKYGARNWSLISKYIKGRSGKSCRLRWCNQLSPTVEHRPFTAAEDEAILAAH
DKYGNRWATIARLLPGRTDNAVKNHWNSTLKRRSQLNQQKKIG
>RMYB67
LCSNCTNFSAYFSHLIPQIPSIKLSIPFLLLFSNRLSTTQGKMDSADRDAHATPSTWTWR
EDKDFEDGLVEFPDGYPNRWVRIAEKMQTKTPAEVERHYAVLLEDVAAIDAGLVDLPKYG
VNNEIELRSSSEADRRNPKSEKKPVTGQRKAAKAWTDDEHKAFLAGLKKYGKGDWKSISR
YSVRTRSPAQVASHAQKYYERQEKEVQSKKRRSIFDNSIEESS
>RMYB68
SVIIRVFQNCCCISMAAMAKKVAPEAVPERVKGPWSPEEDELLTKLVQQHGARNWTLISQ
SIPGRSGKSCRLRWCNQLAPEVEHRPFTEEEDEIIVQAHAVHGNKWATIAKMLTGRTDNA
VKNHWNSTLKRKSYSGRSDGGGARLPQALRRLDNRDVSAAIIYESESSC
>RMYB69
MQEAELRKGPWLEEEDQQLADAVAVLGERRWDALAKASGLKRGGKSCRLRWMNYLRPNLK
HGNITAEEERTIVELHEKWGNKWSRIAQRLPGRTDNEIKNYWRTYLRKKNHVLEKD
>RMYB3R-1
YKGKNWKKIAECFRDRTDVQCLHRWQKVLNPDLVKGPWSKEEDEKIIELVEKYGPKKWST
IAQNLPGRIGKQCRERWHNHLNPNINRDAWTQEEEVILIRAHQMHGNKWAELTKYLPGRT
DNAIKNHWNSSVRKKLDMYMSSGVLQQLQDVPLVSHSNHLTASSSSRGKQGSEDDILEGG
VEVEEASECSQGSAIASMSQSINNTIALLGECRTTEDTSLIPFSAEDYRPVFQEPAFLIP
EVPCEQGDKFPEHDFSIDWEIFEE
>RMYB3R-2
MEGLKSDDRCLENKDITVDSSSSVSENSSTIALKSTGISSPSSTSPAHKRTSGPIRRAKG
GWTPEEDATLKKAVAAFRGKCWKKIAEFFPDRSEVQCLHRWQKVLNPELVKGPWTQEEDD
KITELVAKYGPTKWSIIAKSLPGRIGKQCRERWHNHLNPHIKKDAWTLEEELTLLNAHRV
HGNKWAELAKLLPGRYLYRAHLETLNL
>RMYB3R-3
MEGLKSDDRSLENKDITVDSSSSVSENSSTIALKSTGISSPSSTSPAHKRTSGPIRRAKG
GWTPEEDATLKKAVAAFRGKCWKKIAEFFPDRSEVQCLHRWQKVLNPELVKGPWTQEEDE
KITELVAKYGPTKWSIIAKSLPGRIGKQCRERWHNHLNPHIKKDAWTLEEELTLLNAHRV
HGNKWAELAKLLPGRTDNAIKNHWNSSLKKKLDFYLATGNLPPVSKCVSLNDAAADIHKT
ASAGELHVGSNKGSNSAHLASCETTDLRKADDRRTDMPEERSKDYNVASHPQMEPTNPES
TKHHEAMQSESGAVNPTKESEFEKQHTFGGVGRYNIVKASLRYEMPVHGTLCYEPQTFDN
YGQLDSTLRNVSLMQSQSDTCPATPPTPLFTPPSVRGGNFSAQSPESILRIAAKSFRNTP
SILRKRKLDMQPKELVVPSGEHNHTNGSENAGLQSKTSCADPVSDDEDGVSSNKSPNSSP
PYRLNFKRTSVHKSVEKQLDFTTITDQEACNNNTKSGDSKVKEARPVTKFVYTRQRRGLK
SSVAKCN
